# Mitotic gene conversion can be as important as meiotic conversion in driving genetic variability in plants and other species without early germline segregation

**DOI:** 10.1371/journal.pbio.3001164

**Published:** 2021-03-22

**Authors:** Xianqing Jia, Qijun Zhang, Mengmeng Jiang, Ju Huang, Luyao Yu, Milton Brian Traw, Dacheng Tian, Laurence D. Hurst, Sihai Yang

**Affiliations:** 1 State Key Laboratory for Crop Genetics and Germplasm Enhancement, Nanjing Agricultural University, Nanjing, China; 2 State Key Laboratory of Pharmaceutical Biotechnology, School of Life Sciences, Nanjing University, Nanjing, China; 3 Institute of Food Crops, Jiangsu Academy of Agricultural Sciences, Nanjing, China; 4 The Milner Centre for Evolution, Department of Biology and Biochemistry, University of Bath, Bath, United Kingdom; Stowers Institute for Medical Research, UNITED STATES

## Abstract

In contrast to common meiotic gene conversion, mitotic gene conversion, because it is so rare, is often ignored as a process influencing allelic diversity. We show that if there is a large enough number of premeiotic cell divisions, as seen in many organisms without early germline sequestration, such as plants, this is an unsafe position. From examination of 1.1 million rice plants, we determined that the rate of mitotic gene conversion events, per mitosis, is 2 orders of magnitude lower than the meiotic rate. However, owing to the large number of mitoses between zygote and gamete and because of long mitotic tract lengths, meiotic and mitotic gene conversion can be of approximately equivalent importance in terms of numbers of markers converted from zygote to gamete. This holds even if we assume a low number of premeiotic cell divisions (approximately 40) as witnessed in *Arabidopsis*. A low mitotic rate associated with long tracts is also seen in yeast, suggesting generality of results. For species with many mitoses between each meiotic event, mitotic gene conversion should not be overlooked.

## Abstract 2

Gene conversion associated with meiosis has long been a focus of attention in population genomics, but mitotic conversion has been relatively overlooked as it was thought to be rare. Analysis in plants suggests that this could be a mistake; long tract lengths and multiple mitoses in species lacking germline sequestration suggest that mitotic conversion, although rare per mitosis, should not be ignored.

## Introduction

Core population genetical parameters such as the mutation rate [1–4], the meiotic crossover (CO) rate and the rate of and biases in meiotic gene conversion (GC) [5–8] are increasingly being well defined and their influence better understood. For example, owing to a GC:AT bias in heteroduplex mismatch repair [9], meiotic GC has emerged as a dominant mechanism to explain G+C content variation within and between genomes, not least because it can potentially explain the intragenomic G+C–recombination correlation [10–12] seen across the eukaryotes in outbred species [13], of which mammalian isochores are one probable manifestation [14–16]. Whether biased or not, GC is also an important process modulating diversity levels [17].

While the role of meiotic recombination and associated GC is increasingly a focus of interest within evolutionary genomics, mitotic recombination and its associated GC is relatively understudied (for compendium, see S1 Table). Mitotic recombination maintains genome stability through the repair of DNA double-strand break (DSB) [18–20]. In principle, it can occur via any 1 of 3 mechanisms of homologous recombination (HR): canonical DSB repair (DSBR), synthesis-dependent strand annealing (SDSA), and break-induced replication (BIR) (reviewed in [21,22]). We are here not concerned with dissecting the exact mechanism, but rather wish to estimate the relative importance of meiotic and mitotic recombination and associated GCs, using rice as a model plant.

Mitotic recombination involves both somatic crossing over events and allelic conversion events. A priori we expect the former to be less relatively influential compared with meiotic processes. The effects of crossing over are on allelic reassortment. Through the meiotic process chromosomal segregation is the major determinant of reassortment, meiotic crossing over tending to be a second order influence [23], this being especially true when much crossing over is telomeric. As there is no mitotic equivalent of chromosomal segregation, mitotic recombination exerts all of its reassortment effects through crossing over events, thus rendering mitotic crossing over almost certainly a minor order effect on net allelic reassortment in any regularly sexual species, this being compounded by the rarity of such events (S1 Table). By contrast, the net effect of GC in meiosis and mitosis are directly, and hence fairly, comparable in their influence. For this reason, we concentrate on the relative importance of GC effects between mitosis and meiosis.

To determine the relative importance of processes, we need to define a metric of their influence. While at any given chromosomal position the relative impact of crossing over on allelic reassortment is dependent on the event rate alone, the relative impact of GC events is dependent on the event rates and the number of markers converted per event. The latter depends on the size of the conversion tract and the number of polymorphic markers within the span converted. The relative importance of meiotic to mitotic conversion we thus consider in terms of the ratio of alleles converted per generation from zygote to gamete via the 2 processes (with tract length as a maker density-independent proxy). The relative importance of meiotic to mitotic crossing over we measure in terms of the ratio of the number of events per generation (from zygote to gamete) via the 2 processes at a given locus.

Prior evidence suggests that mitotic recombination events, while important as a break repair mechanism [24], are rare. In both tobacco and *Arabidopsis*, the mitotic recombination rate is around 3 × 10^−5^ − 10^−6^ events per reporter site per cell generation (details in S1 Table). Low rates are also reported in yeast [25–28] (see S1 Table) and in some model animals, such as fly, mouse, and human [29–32]. Given the low event rates, it seems reasonable to suppose that as a modulator of heritable variation, GC associated with mitotic recombination is likely to be of negligible importance when compared against rates of meiotic conversion. We suggest that, especially in species with unsequestered germlines, such as plants, any such assumption may need to be questioned.

The absence of early germline segregation in plants has several interrelated consequences [33]. First, as plants do not sequester germline so early, restricting the number of mitoses between zygote and any given gamete is not as easy as in animals. For example, while the female human germline is estimated to have possibly as few as 24 mitoses from zygote to gamete [34], even in small and short-lived *Arabidopsis*, a comparable number is 40 divisions [35]. For longer-lived plants, the number of mitotic events—and, hence, opportunities for mitotic recombination—is potentially considerably higher. In addition, for those plants with long life spans, somatic recombination may be particularly important as it is necessary to protect genomes from DNA damage [36,37].

Second, events in many cells of plants have a potentially long “future shadow” as the resultant genomic changes could transmit to the next generation. By contrast, in animal somatic tissue, the same is not true. This introduces in turn a source of between-gamete variation owing to differences between branches of the same plant, e.g., bud sports [38,39]. Each year, a plant, especially a perennial, has a large number of buds, each of which can be different (via mutation or mitotic GC or recombination) [40–42]. This can be visually obvious, such as a pinot noir vine with red grapes generating, via mitotic recombination, white grapes [38]. Bud sporting has been successfully used to breed new varieties of commercially relevant flowers and fruits [39,43–45].

The effect of this on the relative importance of mitotic recombination is not, we suggest, trivial. While late sequestering species will have a relatively high proportion of body cells within which mitotic events (mutation, GC, CO) will be transmissible to progeny, the proportion of progeny receiving any given event may be relatively low compared to a germline event in an early sequestering species. Assuming, however, that the length of the future shadow predicts the strength of selection on mutation and repair processes [4], late germline sequestration may be expected to reduce absolute rates of mutation and increase investment into repair processes [4].

A further reason to question the relative influence of mitotic and meiotic GCs, unconnected to germline timing, is owing to a probable difference in tract length between meiotic and mitotic GC events. Prior evidence suggests that this difference might be substantial. From analysis of tetrads in 4 different species, 92% of meiotic GC tracts were less than 5 kb and 99% were less than 10 kb [8]. By contrast, mitotic conversion tracts >100 kb are reported [27] (S1 Table). In assessments of mitotic recombination in the best-studied species, yeast, several GC events with extremely long tract length have been observed, there being a median size of >40 kb, some over 120 kb [27,46], as opposed to 2 kb in meiosis [8].

For the above reasons, we consider it worthwhile to estimate the relative influence of meiotic and mitotic GC. We are unaware of reports attempting the same, although mitotic and meiotic recombination rates have been compared in *Arabidopsis* with the conclusion that they are more similar than observed in yeast [47]. To emphasise that these mitotic events are potentially transmissible, we choose not to employ the term somatic recombination as, by definition, the events need not be somatic (sensu strictu).

While underestimation of the net impact of mitotic conversion may be possible, detection of mitotic recombination events remains difficult as it is dependent on a low event rate. Indeed, because event rates are commonly so low, some approaches to their study include induction of events by DNA damage [48], assessment of DNA repair-defective mutants [28,49,50], and use of site-specific meganucleases such as HO or Cas9 [51–53]. These do not address the rate and effects of the process in vivo, let alone in the more evolutionary relevant field environment (as we attempt).

Alternative approaches use nutrient- or color-based selective systems of auxotrophic heteroalleles in yeast [27,54,55], a fluorescent reporter system in fruit fly [56,57] and a β‐glucuronidase (GUS) or luciferase (LUC) reporter system in *Arabidopsis* [37,58–61]. Although a reporter system reduces the ability to infer a genome-wide absolute rate of mitotic GC, we can consider the relative rates of meiotic and mitotic recombination [47]. This we do by consideration of the observed rates of the 2 processes at the same reporter locus employing the same markers and the same reporter methodology.

**Fig 1:**
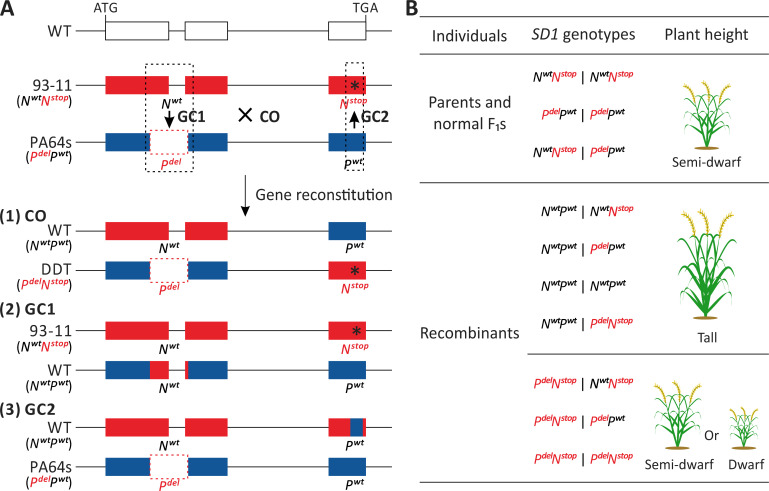
Plant height-related system used to detect recombination events at the *SD1* locus. **(A)** Two semidwarf modern rice cultivars, 93–11 and PA64s, each homozygous for defective *sd1* gene caused either by a single-base substitution (C->G, premature termination, marked as *N*^*stop*^, black asterisk) or a 383-bp deletion (frameshift, marked as *P*^*del*^, red dotted frame), respectively. Then, the genotypes of the *SD1* alleles in WT, 93–11, and PA64s were denoted as *N*^*wt*^*P*^*wt*^, *N*^*wt*^*N*^*stop*^, and *P*^*del*^*P*^*wt*^, respectively. For progeny of a cross between these 2 varieties, if a CO event or a gene conversion (GC1 or GC2) event occurred between the PA64s and 93–11 haplotypes in mitosis of F_1_ individual at the *SD1* locus, a recombination haplotype with a wild-type *SD1* gene (i.e., *N*^*wt*^*P*^*wt*^) would be generated. Black dotted frames show a possible schematic of conversion tracts. **(B)** *SD1* genotypes of F_1_ individuals and their corresponding expected plant heights. Two parents and nonrecombinant F_1_s would present semidwarf statures, around 90–120 cm; while recombinant individuals with the wild-type *SD1* gene would show tall statures (>130 cm), contrarily, recombinant individuals with the DDT *SD1* gene are semidwarf or dwarf. Defective alleles are shown in red. CO, crossover; DDT, double-defective type; GC, gene conversion.

To this end, we employ our method [62] for detecting both mitotic and meiotic CO and GC events at 1 specific locus (*SD1*). Hybrid plants (termed LYP9) have 2 different defective alleles (a frameshifting indel and a premature stop codon), thus enabling the possibility of recombinant rescue of the wild-type tall growth phenotype (>130 cm) by mitotic recombination (see Materials and methods, Fig 1 and S1 Fig). From visual inspection, it is trivial to identify the tall plants (see S2A Fig). After selecting these, we then employ genotype changes at the targeted sites and their flanking regions to exclude false positives and classify events (see S1 Text and S3 Fig).

We then model plant ontogeny to determine expected ratios of the 2 processes summed over all the time from zygote to gamete for an arbitrary number of cell divisions. To contribute to plant height, any such events are most likely to have occurred at a very early developmental stage. Indeed, as in all cases, the same recombination event is seen in root, and as root and shoot are determined after just a few cell divisions, we can use the relative abundance in root (where *SD1* is not expressed [63]) to estimate the per mitosis event rate. With knowledge of the sizes of tract, we can then estimate the impact on allele conversions from zygote to gamete compared with meiotic events at the same locus, also from zygote to gamete (although the meiotic events only feature in the very last step from diploid to haploid gamete). Early developmental events are also those most relevant to onwards heritable transmission as they are expected to dominate the plant. We demonstrate such transfer to the F_2_. Even assuming a conservatively low number of mitotic divisions, the models suggest that mitotic and meiotic GC can be of approximately the same order of magnitude as regards the number of alleles converted.

## Results

### Mitotic recombination is uncommon

To characterize rates of mitotic recombination, we assessed approximately 1,100,000 LYP9 individuals (F_1_s of PA64s × 93–11) planted under common garden conditions in a 1.53-ha farmland in 2017. A total of 26 tall individuals with plant height >130 cm were found. While heterosis is a priori unlikely, there are, however, other imaginable reasons for recovery of growth other than recombination within our defective growth controlling locus. In our experience, there is very little variation in plant height of LYP9s (see also below), so that those >130 cm are unlikely to be simply unusually tall plants without some other underlying cause. We nonetheless expect false positives (in terms of phenotypic screening). Subsequent genotyping of the *SD1* gene in these individuals revealed 24 samples harboring a wild-type *SD1* allele (*N*^*wt*^*P*^*wt*^) (S2 Table) while 2 samples lacked recombination at the *SD1* locus. These 2 false positives were also not especially tall while the true revertants had a mean height of 149.04 cm, s.e.m. = 2.1 (*sd* = 10.4, *n* = 24). The false positives were excluded from further study.

From the distribution of heights of wild-type *SD1* individuals, we surmise that the 130-cm cutoff might cause us to miss 3% of true recombinants ([130 − 149.04]/10.4 → Z = −1.83 → 3% lower tail), this approximating to less than 1 plant in our sample (if 24 observed = 97%, 100% = 24.7). As a negative control, we previously examined 98 random average sized individuals (in the range of 100 to 120 cm) and found no CO or GC events at the locus [62].

### Nonsense mutations are unlikely to explain observations

Genotyping all 24 true *SD1* revertant samples using Sanger sequencing shows that all harbored the same genotype as PA64s at the premature stop codon site, i.e., all had the same G → C reversion. This supports the hypothesis of GC rather than mutation at the stop site, as the stop codon could have been destroyed by either G → C or G → T mutations or created by a G → A event (which we do not see), while GC requires these to be exclusively G → C. We can estimate the probabilities with knowledge of the rate of point mutation per site, this being 1 × 10^−8^ per bp per generation [2]. This means that we expect a total of 0.003663 G → C revertants in 1.1 million individuals at this site. If so, then the probability of having a G → C not by mutation is (24 − 0.003663)/1.1 × 10^6^ = 2.18 × 10^−5^. Then, from Bayes theorum, at any given site in a given individual, the probability of mutation having occurred given that a G → C has been observed is: *P*(mutation|G → C) = 1/3.10^−8^/[1/3.10^−8^ + 1.(24−0.003663)/1.1 × 10^6^] = 0.000152, and hence, *P*(not mutation|G → C) = 1−0.0015 = 0.99984. The probability that none of the 24 have a mutation is thus 0.9998^24^ = 0.996. Thus 99.6% of the time with our observations, all the 24 G → C events are not owing to mutation.

### Estimating the rate of gene conversion

**Fig 2:**
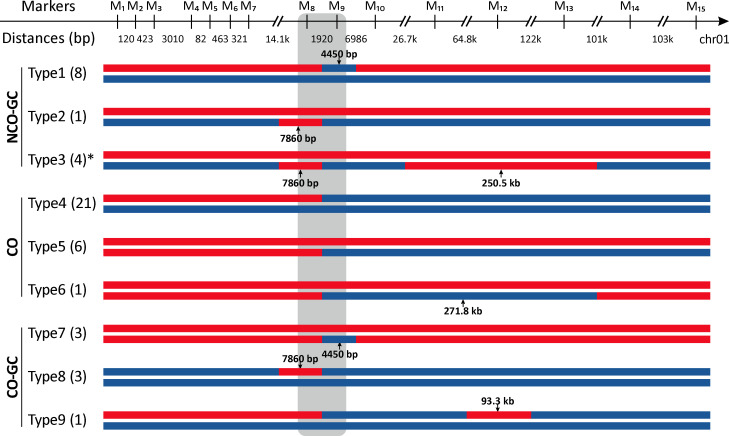
Outcomes of mitotic recombination at *SD1* locus in 48 F_1_ individuals. For 48 F_1_s (24 LYP9 F_1_s and 24 F_1_s from other crosses), genotypes the same as 93–11 and PA64s are marked in red and blue, respectively. In summary of 2 datasets, we found 29 CO (in LYP9 lines, 18 CO; in other 4 crosses, 11 CO), 17 NCO-GC (in LYP9 lines, 3 NCO-GC; in other 4 crosses, 14 NCO-GC) and 7 CO-GC (in LYP9 lines, 3 CO-GC; in other 4 crosses, 4 CO-GC) events had occurred in 48 tall individuals. According to the patterns of genotype switches, here we classified outcomes detected in our assessment into 9 types. Type1, Type2, Type4, and Type5 match the canonical pattern of mitotic recombination (Section A in S1 Text and S1 Fig), and the remaining types with complex genotypes are caused by multiple events. In simplest terms, Type3 and Type6 are caused respectively by 2 adjacent NCO-GC events and 2 adjacent CO events, and Type7 to Type9 seem to come from a conversion event accompanied by a CO event. Detailed patterns of recombinant individuals from LYP9 lines and other hybrid varieties are summarized in S8 Table. Two markers, M_8_ and M_9_, are in the *SD1* gene, which are indicated by the grey box. Numbers under tract bars indicate the tract lengths. Number of individuals of each type are shown in parentheses, and numbers under coordinates show distances between 2 adjacent markers. *, the fine-scale dissection of recombination events of Type3 is shown in S8 Fig. CO, crossover; GC, gene conversion; NCO, noncrossover.

In 24 tall LYP9 (F_1_) individuals, we identified a total of 3 NCO-GC events, 18 CO events, and 3 CO-associated GC (CO-GC) events at the *SD1* locus (Fig 2 and S2 Table). In H17 and H18 (Type 8 in Fig 2), we observe a GC event associated with a CO event out of the *SD1* region. Similarly, H5 (Type 9 in Fig 2) is likely to have resulted from a relatively long tract conversion (93.3 kb) associated with a CO event. Collectively, with respect to the rate of GC per marker, we estimated that GC involving the *SD1* locus per marker per haplotype occurs at a rate of 1.36 × 10^−6^ per marker per haplotype per plant (3 NCO-GCs and 3 CO-GCs for 2 haplotypes and 2 markers, (3+3)/1,100,000/2/2 = 1.36 × 10^−6^), which is lower by 2 orders of magnitude than our estimate (3.3 × 10^−4^) of the meiotic rate of GC at the same locus in rice [62].

### No evidence for seed contamination

To have confidence in our rate estimates, especially for an event this rare, we need to be confident that the 24 individuals are true mitotic recombinants and not owing to other causes such as seed contamination. We approach this by 2 routes: looking for intraplant mosaicism and for high heterozygosity.

LYP9 plants should have heterozygosity throughout the genome, while seed contaminants (e.g., possibly LYP9 progeny) would not. Of the 24 tall individuals that passed the false positive test, eight were selected randomly for whole genome sequencing (WGS). We confirmed that all of them are LYP9 lines with a range of 93.68% to 98.42% heterozygous markers from 93–11 and PA64s detected (S3 Table). The remaining 16 tall individuals were also confirmed for the LYP9 background by 13 flanking markers (S4 and S5 Tables), which cover an approximately 445 kb span around the *SD1* locus (S2 Table).

Mitotic recombination, unless in the zygote, predicts intraplant mosaicism, while seed contamination does not. Curiously, in our data, no matter by WGS or marker-based genotyping (S2 and S3 Tables), only homozygous recombinant cells were found in (late stage) flag leaves arguing against mosaicism. To examine further, we randomly selected 9 recombinant lines to evaluate proportions of recombinant cells in roots, basal leaves, and flag leaves (S5 Fig, details in Materials and methods). All basal leaves retained a small proportion of nonrecombinant cells, and all samples, except H17, retained nonrecombinant cells in the roots of main culms (proportions from 0.01% to 13.38%) (S6 Fig and S6 Table).

While the result thus supports the hypothesis that mitotic recombination is the cause of the growth phenotype, it leaves an enigma: Why do the late stage leaves uniformly contain the growth promoting gene but early leaves and roots not? This might be owing to our selection system or intraorganismic between-cell competition. That the proportion of recombinants is significantly higher in flag leaf than in roots in the same plant is indicative of some form selection (Wilcoxon test, *n* = 9, *P* = 0.0065) and bears resemblance to proliferation of *Apc*−/− cells generated by mitotic recombination in *Apc*+/− mice [31,64]. This result deserves further scrutiny. Stimulation of early recombination by site-specific meganucleases [51–53], so obviating a between-plant selection explanation/ascertainment bias, would be informative.

### Mean length of mitotic conversion tracts is 30 to 50 kb

From the analysis of the 24 LYP9 recombination events, it is clear that there is more than one sort of event happening. To provide a fuller catalog of the spectrum of mitotic recombination events at the locus, in particular, to estimate the length of conversion tracts, we expanded our efforts to 4 additional semidwarf hybrid rice varieties (F_1_ progeny) grown in expansive natural farmland planted by farmers around Nanjing in 2017 (S2 Fig). Given that we do not know the size of the population from which these were sampled, they cannot provide rates per plant. To efficiently screen the recombinant lines, a more stringent standard (plant height ≥ 150 cm) for collection of tall individuals was used here.

In separated fields of these 4 hybrid rice varieties, a total of 24 tall F_1_ individuals were collected along with 3 semidwarf individuals adjacent to each tall individual as controls. After genotyping the *SD1* gene and its flanking region of these tall individuals and their controls using the same 15 markers as used for detection of 93–11 and PA64s, we found that all samples from the 4 crosses could be identified by the same nucleotide polymorphisms in the *SD1* region. All of the tall individuals harbored at least 1 wild-type *SD1* gene, whereas the controls with semidwarf plant height harbored 2 defective *sd1* alleles (S7 Table). Additionally, for each of these additional crosses, genotyping by these 15 markers in the *SD1* locus also confirmed that these tall F_1_s and their surrounding semidwarf individuals shared the same parents, confirming that they come from same cross. Taking all tall individuals from 2 datasets together, these tall individuals were then studied to characterize mitotic recombination events at the *SD1* locus.

After genotyping the *SD1* gene and its flanking regions, we classified the changes from the parental genotypes in relation to possible recombination types (S1 and S3D Figs). Specifically, in mitosis, outcomes of CO events present as heterozygous genotypes on one side of a breakpoint and homozygous genotypes on the other side, whereas noncrossover-associated gene conversion (NCO-GC) events resolve with heterozygous genotypes on both sides of the tract but homozygous genotypes within the tract (S1 Fig). In addition, given at least the theoretical possibility of CO interference [65], meaning that the probability of 2 CO events occurring in a short region (<100 kb) is extremely low, we allowed that outcomes with homozygous genotypes on both sides of the long tracts (>100 kb) may come from 2 adjacent CO events, whereas those of the short tracts may come from a CO event out of the *SD1* locus with a conversion event in the *SD1* gene (S4 Fig). Additional complex patterns could not be resolved in a small subset of individuals, including combinations of multiple genotype switches that could be caused by long-tract conversion, CO-associated conversion, or repetitive CO events.

As noted above, in the 24 LYP9 (F_1_) individuals, we observe 3 NCO-GC events, 18 CO events, and 3 CO-associated GC (CO-GC) events (Fig 2 and S3 Table). A GC event associated with a CO event out of the *SD1* region is observed in H17 and H18 (both Type8 in Fig 2). H5 (Type9 in Fig 2) may reflect a relatively long CO-associated tract conversion (93.3 kb). In tall individuals of the other 4 crosses, after identifying genotypes of the *SD1* region by the same strategy as with LYP9 individuals (details in Materials and methods), we identified a total of 14 NCO-GC events in 10 individuals, 11 CO events in 10 individuals, and 4 CO-associated GC (CO-GC) events in 4 individuals (S7 Table). Noticeably, for LLY1 to LLY4 (Type3 in Fig 2), which are independent from each other according to WGS data judged by the existence of strain-specific marker combinations (S1 Data and S3 Table), all harbor both a relatively short NCO-GC event and an extremely long NCO-GC event, these being defined as 7,850 bp and 250.5 kb by the midpoint method.

Employing all GC events, with the midpoint method, we estimate an average tract length of 50.3 kb in rice mitosis, this being somewhat comparable to that seen in yeast, estimated to be 30 to 40 kb [27,46].

We are cognizant of limitations in our methodology that may have important consequences for this 50 kb estimate. Central to these is usage of the midpoint method as a means to call track sizes when marker density is low or variable. This is especially acute when a span is called based on the conversion of 1 marker as the size of such a conversion tract will be called dependent on the intermarker distance of flanking markers. For example, 1 long Type 9 tract (>90 kb) is associated with only 1 marker and called this long owing to low marker density in this region. We note too that the 250 kb tracts are without precedent. We do not therefore suggest that the above estimates are robust as estimates of absolute sizes.

In principle, estimation error need not be a serious issue for our mode of analysis as we seek to compare mitotic and meiotic events, using the same set of markers. Usage of the same set of markers for meiotic and mitotic estimation should tend to make errors cancel when considering the ratio of the two. However, this need not hold (or be safe) if there is variation in intermarker interval associated with meiotic and mitotic events (e.g., owing to regionalization of events and highly variable marker density). To overcome this, we performed whole gene sequencing on the longer somatic recombination events to attempt a finer-scale resolution of tract lengths. We focused on the putatively extremely long tract sizes as estimation error when intermarker distances are small is by necessity also small, even if only 1 marker is converted.

In the previously defined 250 kb region covering M11, M12, and M13, we found at least 25 reliable markers (with high depth and not in repeat regions) (listed in S4 Table). This region is then split into 2 conversion tracts (red tract in S8 Fig): One is about 98 kb, the other one is about 49.9 kb to 77.9 kb. For LLY2, which had poorly covered reads in this region, the result was validated by PCR results. In view of the low marker density defining the 90 kb tract in Type9, which we could not resolve via WGS, we removed this from mean tract size calculation. Under these conditions, the average length of a mitotic conversion tract is approximately 28.5 kb (this is close to possibly the best resolved mitotic tract length estimate in yeast [27], 30.79 kb). Note that all these Type 3 samples have different conversion tract sizes in this finer scale analysis.

### Mitotic tracts are longer than meiotic tracts in rice and yeast

Above, we generated 2 estimates for mean mitotic tract length (around 50 kb from low-resolution markers and 28.5 kb from WGS data). Both estimates differ markedly from what is seen in meiosis. Using the same low-resolution markers, we estimate a mean meiotic tract size of 9.8 kb (length range: 0.4 kb to 16.4 kb) using the same detection system at the same locus in rice [62]. From our prior analysis [7], we can also determine a higher-resolution WGS-based estimate, this being 3.121 kb. The mitotic estimates are also different from the distribution of observed tract lengths in meiosis in another 4 species [8]. In yeast, the mean meiotic tract size is about 2 kb [8], while in the 3 other species (*Neurospora*, *Chlamydomonas*, and *Arabidopsis*), mean tract sizes in meiosis are under 1 kb [8].

In rice, with an average tract length of 50.3 kb or 28.5 kb in mitosis and of 9.8 kb or 3.12 kb in meiosis, we see a ratio of 5:1 (50.3:9.8) in lengths (mitotic:meiotic) for low-resolution estimation and 9:1 (28.5:3.12) for the higher-resolution data. The most conservative estimate would be to use the smaller WGS mitotic span and the longer low-resolution meiotic span, giving a 3:1 (28.5:9.8) length ratio. The ratio is probably more extreme in yeast as there the meiotic tracts are considerably smaller than in rice, while mitotic events are not greatly dissimilar. From 58 mitotic tracts in yeast (from Table S4 of [27]), the mean length of mitotic tracts is 30.79 kb, while that of the meiotic tracts in 2 kb derived from the studies of Mancera and colleagues [5] and Liu and colleagues [8], giving a ratio of 15:1.

### Relative rates of different event types are similar in meiosis and mitosis

While tract lengths differ, it is also of value to ask whether, for mitotic and meiotic recombination events, the relative proportion of CO and GC events differ between the 2 processes and differ from what we see in yeast. Consistency between what we see in rice mitosis and yeast mitosis provides a form of “sanity check.” Any difference between meiosis and mitosis in the same species would need to be factored into consideration of the net effects of the two.

Combining the 2 datasets (from the LYP9 1.1 million plants and the 4 other crosses), we found 29 CO (in LYP9 lines, 18 CO; in the other 4 crosses, 11 CO), 17 NCO-GC (in LYP9 lines, 3 NCO-GC; in the other 4 crosses, 14 NCO-GC), and 7 CO-GC (in LYP9 lines, 3 CO-GC; in the other 4 crosses, 4 CO-GC) events had occurred in 48 tall individuals (summary in S8 Table). With respect to the relative frequencies of CO to NCO-GC events in mitosis, these data therefore indicate a ratio of 36 CO events (29 CO and 7 CO-GC) to 17 NCO-GC events, or 2.11:1 at the *SD1* locus. This is highly similar to the previous estimate (34 CO versus 16 NCO-GC, 2.12:1) at the *SD1* locus in meiosis [62]. Furthermore, 29.2% (here a total of 17 NCO-GC and 7 CO-GC, 7/(7+14) = 29.2%) of the conversion events in spontaneous mitotic recombination in our data are associated with COs, which is close to estimates of mitotic recombination events in yeast (27%, χ^2^ = 0.086, *P* = 0.77) [27,46] or meiotic recombination at the *SD1* locus in rice (33.3%, χ^2^ = 0.27, *P* = 0.604) [62].

### Mitotic and meiotic conversion may convert approximately the same number of markers per generation

Our observed event rate of mitotic conversion is lower by nearly 2 orders of magnitude than the comparable event rate of meiotic GC at the same locus using the same methods in rice [62]. However, to compare between the two, we need to allow for the fact that developmentally early events can affect all or nearly all subsequent meioses, that mitotic events in plants are multiple, and that tract lengths differ between mitotic and meiotic events.

To evaluate the relative impact on progeny of mitotic conversion and meiotic conversion, we model conversion events that occur in mitosis and that could be inherited in progeny (in effect, we model ontogeny of the stalk) (S7 Fig). We assume that there is no cell lineage competition between recombinant cells and nonrecombinant cells. Employing our assessment of meiotic conversion features [62], we considered the relative contribution from zygote to gamete of allelic change owing to mitotic and meiotic conversion (details in Materials and methods).

Core to the mitosis/meiosis comparative analysis are assumptions made about the per mitosis event rates. Information on the frequency of the recombination event in roots, aligned with assumptions about the timing of the root–shoot ontogenetic split and the number of stem cells involved, allow us to place reasonable estimates on per mitosis rates that can be compared to estimates from meiosis. We consider 4 viable ontogenetic models to derive per mitosis rates (details in Section C, S1 Text). With these, we estimate rates in the range 4.63 × 10^−6^ − 1.68 × 10^−5^ per marker pair per mitosis. We estimate the comparable figure for meiosis to be *R*_*me(GC)*_ = ~2.66 × 10^−3^. We employ as a rough guide 40 mitotic divisions per generation [35] as this is derived from a physically small short-lived annual (i.e., *Arabidopsis*) and thus most likely to be a conservative estimate.

**Fig 3:**
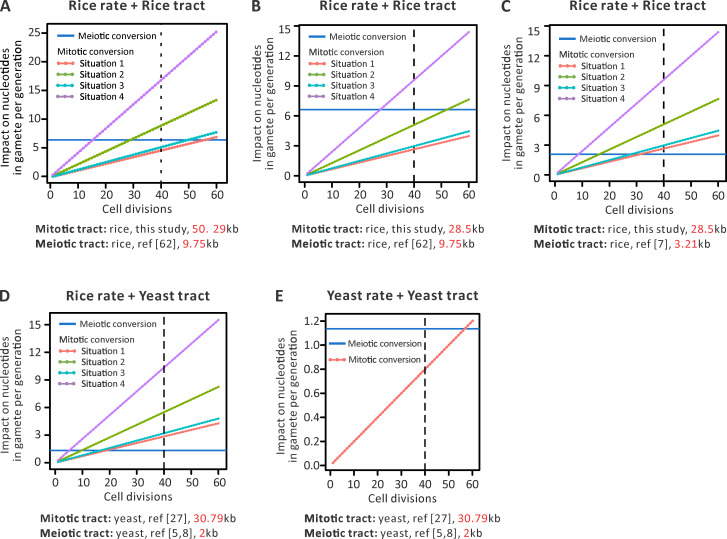
Impact on nucleotides in a gamete per generation for meiotic and mitotic conversions, as a function of the number of cell generations from zygote to gamete. To investigate the relative impact on progeny of mitotic conversions and meiotic conversions, we considered 5 analysis (details in Materials and methods): (A) *N* divisions in rice employing rice-specific rates of events as well as tracts sizes from Fig 2 and Jia and colleagues [62], (B) *N* divisions in rice employing rice-specific rates of events and rice tracts sizes from adjusted size and Jia and colleagues [62], (C) *N* divisions in rice employing rice-specific rates of events but rice tracts sizes from adjusted size and Si and colleagues [7], (D) *N* divisions in rice employing rice-specific rates of events but yeast tracts sizes, and (E) *N* divisions in a diploid yeast but employing yeast-specific rates of events as well as tracts sizes. Data of meiotic conversion rate and tract length in yeast are from Mancera and colleagues [5] and Liu and colleagues [8]. Data of mitotic conversion rate and tract length in yeast are from Yim and colleagues [27]. The line at 40 divisions indicates a conservative benchmark of the number of zygote to meiosis cell divisions for rice, this being derived from rice. Underlying numerical values are presented in S2 Data.

We can then employ our various estimates of the relative meiotic to mitotic tract lengths (3:1, 5:1, 9:1) to determine the proportional effect of meiotic and meiotic conversion, assayed as the relative number of alleles converted, going from any gamete and backtracking to its ancestral zygote. Employing the 5:1 low-resolution marker-derived ratio, we find that the expected effect on progeny of mitotic conversion will be 71% or more of the meiotic conversion effect (Fig 3A) per generation assuming 40 cell divisions. Under one model of early development, parity is reached where there were only 10 to 20 divisions (Fig 3A), while for other models, parity is reached at approximately 50 divisions. Using our most conservative ratio estimate (3:1), congruence takes more cell divisions (Fig 3B), while with the WGS estimate, giving a ratio of about 9:1, there are fewer cell divisions to meiotic–mitotic equivalence than estimated above (Fig 3C). Importantly, all estimates then agree that a combination of multiple mitoses and longer tract lengths can lead to either equivalence after about 40 cell divisions or, at a minimum, a substantial contribution from mitotic conversions.

We note too that in the above, we have erred on the side of caution when calculating mean tract sizes. If we have *M* conversion tracts associated with *N* tall plants (*M*>*N* as some plants have multiple mitotic conversion tracts), giving a total span *S*, then we have assumed a mean tract length of *S*/*M* and *N* events. For the simulations above, we then assume this mean per event. However, one could more reasonably suppose that the mean sum tract length per event is *S/N*; it is just happening to be the case that some events are associated with GC in multiple blocks, not one. The estimate of the mean per event net tract size (in 1 block or many) is then not approximately 28 kb but 40.5 kb (again, we exclude the 90 kb unresolved block). As no correction is required for the meiotic estimate (all *SD1*-associated events were single tracts), no correction is needed for meiotic estimation. Simulating these per event mean sum tract sizes, assuming either 9.75 kb (4:1 ratio) or 3.12 kb (13:1) as the meiotic sizes, again suggests parity between meiosis and mitosis is easily achieved with realistic numbers of cell divisions (S9 Fig).

To be confident that marker density estimation is also not skewing results, we can consider the case that all mitotic conversions associated with a single marker are given the same mean size as meiotic conversions associated with a single marker, this being 6.4 kb. In this case, the mean mitotic tract length (per tract) is 26.7 kb (including Type 9) and 28.9 kb (excluding Type 9), which compares with the 28.5 kb estimate above (that excludes Type 9). The mean per mitotic conversion event sum tract length resolves to 37.9 kb (including Type 9) or 41.0 kb (excluding Type 9) which compares to 40.5 kb (excluding Type 9). This correction thus makes no important modification to our estimates of mitotic to meiotic tract length calculations and hence no meaningful difference to simulation results (S9 Fig).

### Analysis using yeast parameters support a mitotic/meiotic equivalence

Despite the above, it could be argued that all our estimates are rather inexact. Indeed, it can be considered a concern that our estimates, while all qualitatively agreeing, are quantitatively rather divergent. It is then valuable to supplement our results with analysis of the best-resolved system, namely yeast that has, we estimate, a 15:1 mitotic meiotic tract length ratio (see above).

First, we consider our model of plant development with *N* cell divisions between zygote and gamete (*N*~40 for *Arabidopsis*) but using the yeast mitotic and meiotic tract sizes, while assuming rice’s rates of the various processes. Considering rice’s meiotic GC event rate of *R*_*me(GC)*_ = ~2.66 × 10^−3^ and a mitotic event rate estimates derived from our 4 models of early development, we can then estimate the span of tracks converted by the 2 processes (Fig 3D). We find that parity in terms of the numbers of markers converted would be reached between 5 and 20 mitoses.

We can also model yeast specifically. Given that yeast has one of the highest known meiotic recombination rates, we do not necessarily expect the same parity, at any given number of cell divisions. Indeed, from tetrad analysis, we estimated that in any meiosis, 2% of markers are converted in yeast [5,8] which compares to just 0.004% in *Arabidopsis*, 0.007% in *Chlamydomonas*, and 0.03% in *Neurospora* [8]. Not then unexpectedly, the disparity in meiotic and mitotic event rates is higher in yeast than in plants [47]. However, yeast also has a 15:1 mitotic:meiotic tract size ratio, more discordant than we estimate in rice. To investigate the relative roles of meiotic and mitotic conversion, we again considered N divisions in a diploid yeast, but this time, employing yeast-specific rates of events as well as tracts sizes. The analysis in this instance is slightly different as we do not need to assume complex early development, hence yeast present a single estimation of the percentage of markers converted after a given number of cell divisions (details in Materials and methods). We assume an event rate of 1.3 × 10^−6^ per mitotic division [27] and approximately 2% of markers converted per meiosis in yeast (= CMme) and a marker distance of approximately 227 bp (= MDme) [5,8]. We find that expected impact of mitotic conversion will be higher than that of meiotic conversion after 56 divisions (Fig 3E).

Molecular studies suggest that in wild yeasts, sex is exceedingly rare with an outcrossing event once per 50,000 asexual divisions in *Saccharomyces cerevisiae* [66] or sex once in every 1,000 generations in *Saccharomyces paradoxus* [67]. Less clear is what proportion of the time is spent as a heterozygous diploid in which mitotic GC would be most potent (we note however that the population estimates make no allowance for mitotic recombination, assuming that all recombinational diversity is owing to meiosis [67]).

### Mitotic crossing over is unlikely to be influential in sexual species

To compare transmissive probability of somatic and meiotic CO events, we performed a simulation based on a similar model as employed above (details in Materials and methods). After analysis of the same 4 viable models, we find that the expected inheritable probability of somatic CO events could be approximately 10% of COs detected in F_2_ individuals per generation (S10 Fig) (again assuming 40 divisions per generation in plants [35]). The difference between the GC and CO result relates to the fact that CO comparison is dependent on event rates alone, while the GC comparison factors in the proportional difference in GC tract lengths.

### Evidence for transmissibility of mitotic recombination events

The above models presume that the mitotic events are transmissible. Genotyping of 43 F_2_ progeny from selfing crosses of 3 randomly selected tall F_1_ individuals (*SD1* genotype: *P*^*del*^*P*^*wt*^ / *N*^*wt*^*P*^*wt*^) demonstrates that the recombinant wild-type *SD1* genes and tall phenotype were inherited in these progeny (S9 Table). Specifically, in 43 F_2_ individuals, 12 *P*^*del*^*P*^*wt*^ / *P*^*del*^*P*^*wt*^ individuals, 23 *P*^*del*^*P*^*wt*^ / *N*^*wt*^*P*^*wt*^ individuals, and 8 *N*^*wt*^*P*^*wt*^ / *N*^*wt*^*P*^*wt*^ individuals were detected, which consist with the Mendel law of segregation (chi-squared test, *P* = 0.2231). Meanwhile, the allele frequency of *SD1* for progeny of each F_1_ individual also followed the segregation law (S10 Table). This suggests that the early mitotic conversion event affected all or nearly all subsequent meioses of the same plant (consistent with their domination of late stage structures). With lots of subsequent mitoses prior to the gametic tissue, there remains abundant possibility for further such events that can be transmissible by some proportion of the flowers. We, however, found no other evidence for mitotic conversion in the samples we had for WGS.

## Discussion

While variation in rates of meiotic GC is considered core to determining within and between genome variation in G+C content across eukaryotes [10,14,15], and meiotic recombination is considered key to understanding, for example, the efficiency of selection [68], mitotic GC and mitotic recombination are typically overlooked. This is for good reason in many contexts. In mammals, for example, the female germline has very few mitoses, estimated to be possibly as little as 24 [34], may be around 30 [69], and with the event rate being so very low, a priori it appears not to be an especially influential process. There is no mammalian equivalent of heritable bud sports. We argued that in plants, the logic may be somewhat different as regards GC. With long tract lengths and many mitoses that are pregametic, a low event rate per cell division may yet translate to a relevant number of alleles being converted and transmitted to the F_2_. With many mitoses through which recombination can both occur and the products be transmitted to the next generation and with relatively large conversion tracts, the influence of mitotic conversion may be, we estimate, of the order of magnitude of importance as meiotic conversion.

### Caveats and assumptions

The above conclusion, which may seem reasonable in retrospect, requires at least 2 important homogeneity assumptions. First is an assumption that all mitoses have the same recombination rate. We observed that with the root affected in the examined organisms, the mitotic events must have happened very early in development. We use this to estimate per mitosis rates (range 4.63 × 10^−6^ − 1.68 × 10^−5^ per marker pair per mitosis) and assume that through all subsequent mitoses, the same rate applies. This need not be so. Indeed, in humans, it has been considered that the first few postzygotic divisions might be especially mutationally labile, this potentially explaining the high level of chromosomal instability and other errors found in early in vitro fertilization (IVF) embryos as well as the frequency of early pregnancy loss after conception [70–72]. Similarly, mutation rates through spermatogenesis may not be uniform with the earliest stages being more mutagenic [73].

If early cell divisions have higher recombination rates, by the nature of our screen which selects for early events, we will most likely have overestimated the per mitosis CO and GC rates. This would render mitotic CO even less important. A lower bound estimate for the relative importance of mitotic GC would derive from the assumption that our screen detects all conversion events at the *SD1* locus regardless of when in ontogeny they occur. This lower bound would then suggest the net mitotic conversion rate per generation is the observed conversion rate seen in the screen. In this instance, the influence of mitotic GC would be approximately a little over an order of magnitude lower than meiotic conversion (2 orders of magnitude lower mitotic rate, probably under an order of magnitude more markers converted). This is, however, an extreme model as it supposes that our screen might detect a GC that happened, for example, in the cell division immediately prior to the generation of the diploid cell that undergoes meiosis. Given that the screen requires a large change in adult height, this is not realistic. Thus, the 1 to 2 orders of magnitude lower bound on relative importance is an unrealistic lower bound. Where a realistic lower bound might reside is not so clear. However, we note that our estimate of the mitotic recombination rate at 1.5 − 5.3 × 10^−5^ per cell division is similar to the estimate from tobacco at 3 × 10^−5^ using a kanamycin-resistant screen [74], suggesting that our estimate is not exceptional.

Our second homogeneity assumption is that the relative meiotic and mitotic rates that we estimate from *SD1* apply pangenomically. As meiotic rates are somewhat regionalized [7], this assumption may also be problematic. However, for our conclusion to be grossly misleading, the *SD1* locus would have to have an especially high event rate per mitosis and an especially low event rate per meiosis. Under this circumstance, the relative rates would not fairly reflect the rest of the genome in a manner that would render *SD1* too generous to the conclusion of approximate equality. Were *SD1* to have a relatively low mitotic rate and a high meiotic rate, then our assumption of equality would also not hold but would suggest a greater importance of mitotic events above meiotic ones on a genome-wide level. Prior analysis indicated that *SD1* locus has the median number of meiotic recombination events [62]. Whether it is unusual in its mitotic rate is unknown, but we note that our estimate is in line with that in tobacco [74] and not greatly different from that in *Arabidopsis* [47] (S1 Table).

A further caveat considers a limitation of our methodology. By definition, our screen will not resolve very small tract sizes, those that do not enable recombination between our 2 intragenic marker sites (2.3 kb apart). Thus, we likely underestimate the contribution of such small tract conversions. Two factors mitigate this problem. First, these small tracts are also not factored in the meiotic rate estimates as they employ the same 2 markers. In this sense, the comparison is, to a first approximation, “fair.” Second, we note too that tracts that convert no markers are of no population genetical relevance. Thus, “missing” tracts between the 2 markers are in this context irrelevant to the population genetics. However, this same issue could potentially lead to some overestimation of the relative influence of mitotic events. This is because were meiotic events biased to small events (which is known [8]) while mitotic events are not (which is yet to be resolved), then we would be excluding from analysis more small meiotic tracts than mitotic tracts. As some proportion of these meiotic tracts, when considered genome wide, could convert some markers, we would then be disproportionately excluding meiotic conversion events, so resulting in some degree of underestimation of the relative influence of meiotic processes. Given that the “missing” tracts must be small, the net effect of this is unlikely to be of a magnitude to disrupt the conclusion of approximate parity, not least because we chose a reference mitosis number that is itself, at 40 from zygote to gamete, likely to be conservative, so probably underestimating the influence of mitotic events.

### Implications

What implications might our results have? We consider 3 domains: possible impact on interpretation of meiotic crosses, impact on the interpretation of G+C heterogeneity, and possible importance (or lack thereof) of mitotic crossing over.

Regarding the first, imagine that one sequences a parental strain type and then offspring to determine meiotic CO and GC events, assuming that all such events seen are meiotic. In this case, the rates may well be overestimated, as mitotic events not recognised in the strain-type parent, or that are observed in progeny but that occurred in the progeny postfertilization, will be ascribed as being meiotic, when in practice some must be mitotic. A similar problem affects parent offspring estimation of the per generation mutation rate, as de novo somatic mutations in offspring can get ascribed as inherited germline mutations [75]. Similarly, early mutations in the parents are detected in parental soma, so incorrectly considered the unmutated ancestral condition [75].

We suggest there may be one immediate consequence, namely an overestimation of the rate of meiotic double COs in some species. The long GC tracts seen in mitosis can leave the same recombinational footprint as a meiotic double CO (S11 Fig). However, following early yeast studies [5], when considering meiosis, it is common to assume that long tracts (specifically >10 kb) cannot be GCs (nearly all meiotic ones being considered to be small) so must, by assumption, be double COs [5,8]. However, we suggest at some rate they should be unrecognised mitotic conversions. In yeast tetrad data [8], for example, we find at least a total of 105 close “double CO” events (distance between 10 kb and 20 kb) in 29 yeast tetrads (S11 Table). While yeast does have a high CO rate, if a single sequence is considered to represent the parents of such crosses, at some unknown rate, some of these double COs are possibly mitotic conversions.

The importance in plants, however, is less clear as CO interference should prevent the majority of double CO meiotic events in the 10 to 100 kb range, meaning that one should be suspicious of them in the first place. However, we would not rule out a role in plants as class II COs are interference independent [76]. The best-known gene involved in class II COs is MUS81. The null mutation of MUS81 reduces recombination by 10% in wild-type *Arabidopsis* plants and eliminates ca. 1/3 of the residual COs in *zmm* mutants (i.e., those lacking the interference-dependent mechanism) (reviewed in [76]). Thus, there is the possibility of both mitotic GC and meiotic CO creating rare 10 to 100 kb events. As regards long tracts in our data, the effect is not expected to be all that common. We predict that only about approximately 1 long tract should appear in our meiotic data if the mitotic GC rate is near parity (we did not see any).

Similar logic could be extended to explain why in plants the G+C meiotic recombination correlation is not so clear cut. Our sample of mitotic events was not large enough to discern whether mitotic GC is GC:AT biased. However, our much larger sample in meiotic conversion [7] found a significant 1.47:1 ratio of AT→GC conversion, compared with GC→AT events, confirming prior reports [77]. While in many taxa (e.g., mammals) with comparable G+C bias, the G+C–meiotic recombination rate correlation is very clear [15], within plants this is not so, there being considerable heterogeneity of results [78]. Some of this heterogeneity is owing to differences in inbreeding/outbreeding levels [13]. In inbreeders, meiotic heteroduplex formation is rare, given the homozygosity of so many loci (strand invasion may well occur but with no heterozygous sites, a heteroduplex sensu strictu is not formed). However, in addition, we speculate, if mitotic GC is a significant force, but mitotic rates are broadly uncorrelated with meiotic ones, then correlating G+C with meiotic rates alone is likely to dilute any signal, leading to both weaker correlations with meiotic rates and potential heterogeneity between taxa. There may in turn be dependency on longevity and the number of cell divisions prior to meiosis. In addition to inbreeding/outbreeding and the strength of the conversion bias, this provides a further variable to consider when trying to understand variation in the magnitude of the G+C–recombination correlation across taxa.

The role of mitotic CO, as it is conditional on the event rate alone, is likely to contribute very little to overall allelic reassortment, especially given the small effect of more common meiotic CO [23]. It might, however, yet be relevant when considering how obligate asexuals tolerate deleterious mutation, not least because even low levels of CO can have a large genome purging effect [79]. Recent analysis [81] of bdelloid rotifers, a species with no observed males and thought to be anciently asexual [80], indeed shows classical patterns of linkage disequilibrium (LD) decay over genomic distance, consistent with the activity of somatic recombination in an asexual lineage [81]. Similarly, prior analysis indicates that their chromosomal arrangement is incompatible with classical meiosis but, nonetheless, the genome reveals extensive (presumably mitotic) GC [82]. While it is possible that bdelloids may not be truly obligately asexual [81], it is likely, in a rarely sexual organism, that somatic recombination and GC will play a more significant role, as regards protection from deleterious mutations [82], than in regularly sexual species. It also follows that inference of meiotic recombination rates from LD decay data would overestimate the impact of meiotic CO. Theoretical consequences of the generation of buds sports [83,84], and mitotic recombination more generally, in highly heterozygous asexuals is, we suggest, worthy of consideration.

### Segregation patterns provide clues to mechanism

Our experiment was not designed to resolve which possible mechanisms of HR might be involved. Nonetheless, the results may yet speak to this issue. Outcomes of SDSA mechanism are always NCOs, such as Type1, Type2, and Type3 (in Fig 2). CO events in the DSBR model are reciprocal for genotype exchanges (also known as reciprocal crossover (RCO)), while 2 orientations on the mitotic division spindle will generate 2 different outcomes, one of these two will cause loss-of-heterozygosity (LOH) as Type4 and Type5 (as shown in S1 Fig). BIR can also generate LOH events, which is widely reported in mitosis in yeast (reviewed in [85]). In our assay, we cannot clearly distinguish RCO and BIR as causes for Types 4 and 5. In previous studies, LOH events in young cells (cells with only few mitotic divisions) occur by reciprocal recombination, while in old cells, LOH events often involve BIR [86]; meanwhile, most spontaneous LOH events are RCO, and recombination events induced by hydroxyurea are both RCO and BIR [55]. Additionally, DSBR mechanism is more common in plant mitotic recombination, such as in *Arabidopsis* [61] and tomato [52,53]. Thus, in our data, we suggested that these CO-type events are from DSBR.

Many of our events (Type7, Type8, and Type9 in Fig 2) are, by contrast, not explicable by the simplest form of the general DSBR model of mitotic recombination (S1 Fig). These complex mitotic recombination events were also reported in yeast [28]. Both long conversion tracts and this complex switching pattern of genotypes in mitosis, which are unexpected under the canonical DSBR model, suggest some divergence between meiotic and mitotic processes. For those concerned with mechanisms, we suggest this problem is worthy of further scrutiny.

### Final comment

We have argued that mitotic GC may well have either gone ignored or misclassified when considering net rates of GC. But does it really matter if long tracts are owing to mitotic recombination or double meiotic COs or that mitotic conversions are classified as meiotic? The effects, after all, are to a first approximation the same. Comparison with past literature on the origin of mutations suggests we ignore mechanism at our peril. Since Haldane [87–89], we know in humans, for example, that most mutations are derived from the father. One could argue that all that matters is the fact and not the underlying mechanism. Who cares if these were owing to events in male meiosis or in male mitosis? After all, a mutation is a mutation no matter what the origin. Surely, all we need to know is the rate, the mechanism being a dotting of the proverbial i. History would suggest that such a viewpoint is too constraining. The realization of the mitotic dependence of mutation rates has cast focus on the number of germline cell divisions as a means to explain male-biased mutation [34,90] that in turn has stimulated new hypotheses and new appreciations. We now, for example, appreciate that the extent of male-biased mutation is not expected to be constant [91]. As only some mutations are replication dependent, we should also expect between species differences in the relative rates of different classes of mutation as a function of the number of germline cell divisions. Such underlying models also stimulate hypotheses about, for example, a coupling between sperm competition and mutational input [92]. Similarly, understanding that most mutations are mitotic led to study of the mutational risks associated with older fathers [93]. As with male-biased mutation, it is, we suggest, important to distinguish the cause of GC not just for its own sake but also because it enables us to generate similar hypotheses.

## Materials and methods

### The experimental system

The system consists of 2 parental modern semidwarf rice cultivars, 93–11 (N haplotypes) and the thermosensitive male sterile line PA64s (P haplotypes), each homozygous for defective *sd1* alleles. Crucially, the defective alleles are not the same enabling the possibility of recombinant rescue of the wild type. The 2 alleles are here, respectively denoted as *sd1*-*N*^*wt*^*N*^*stop*^ and *sd1*-*P*^*del*^*P*^*wt*^ (Fig 1A). The allele *sd1*-*N*^*wt*^*N*^*stop*^ has intact exons 1 and 2 of the three-exon gene, but a premature stop in exon 3 that negates activity. Conversely, allele *sd1*-*P*^*del*^*P*^*wt*^ has a wild-type third exon but a 383-bp frameshifting deletion trimming part of the 3′ end of exon 1 and part of the 5′ end of exon 2, which also negates activity (281 bp affects coding sequence, 102 bp in intron). The parental strains are homozygous for their respective null alleles and are thus semidwarf (*SD1* being a growth promoting green revolution gene).

The progeny of such a cross, termed LYP9, will initially be *sd1*-*N*^*wt*^*N*^*stop*^ on one chromosome and *sd1*-*P*^*del*^*P*^*wt*^ on the other. As the disabling mutations are in different locations within the gene, if a CO or GC event occurs between these 2 defective alleles, 2 different reconstituted types can be generated in F_1_s (Fig 1A and S1 Fig). If an event occurs early enough through ontogeny, recombinant individuals harboring a wild-type (functional) *SD1* allele are much taller (>>130 cm) than either the parental lines or nonrecombinant F_1_ siblings (range 90 to 120 cm) still harboring the defective *sd1* alleles (Fig 1B), enabling easy visual detection. Note too that plant height in rice is an especially useful phenotype as it is largely unaffected by heterosis (always a possible concern when considering hybrids). Indeed, the mean height of LYP9 is no higher than the mean height of 1 of the 2 parents (strain 93–11) [62]. In rice, heterosis is mainly shown in higher yield and strong stalks [94].

Importantly, we employed the same cutoff (>130 cm) when assaying meiotic conversion rates. As a consequence, we can be confident that the relative rate measure is not skewed by method differences. Our method to determine relative meiotic and mitotic rates is also unbiased by the fact that the screen is directional. Indeed, an individual with a double-defective *sd1* genotype (*P*^*del*^*N*^*stop*^) can also be the product of a CO or GC event but would be expected to become semidwarf or dwarf. However, shorter individuals are both indistinguishable from nonrecombinant F_1_s and more susceptible to the environmental factors. They therefore are not included in our assessment of either meiotic or mitotic rates.

### Rice materials and sampling

Liang-You-Pei-Jiu (LYP9) (F_1_) plants were obtained from the cross of 2 commercially important and well-studied semidwarf rice varieties, namely PA64s (female) and 93–11 (male) [95]. To meet the need for a large number of LYP9 seeds, PA64s, a sterile line of PA64, was used to process a large-scale cross with 93–11, which is photoperiod and thermosensitive genic male sterile. Under high temperature (usually higher than 23.3°C), pollen of PA64s are aborted. By pollinating these flowers of PA64s with pollen collected from 93–11’s flowers, we could conveniently realize the large-scale cross between these 2 parental lines. PA64s is thus the maternal line and 93–11 is the paternal line. The seeds of LYP9 were kindly provided by Jiangsu Academy of Agricultural Sciences (JAAS). We planted over 1,100,000 of the LYP9 (F_1_s) seeds in a 1.53-ha farmland in Nanjing, China, during the normal growing season of rice in 2017. When maturing, nearly all of F_1_s were with semidwarf height (90 to 120 cm, close to their parent 93–11 or PA64s), and 26 individuals with extremely tall stature (taller than 130 cm) were screened out (S2A Fig). Subsequent genotyping of the *SD1* gene of flag leaves in these individuals revealed 24 samples harboring the wild-type *SD1* gene (*N*^*wt*^*P*^*wt*^) (S3 Table). Two samples identified as false positives were excluded from further analysis.

To determine parameters of somatic recombination in other crosses, we expanded our efforts to 4 additional semidwarf hybrid rice varieties (F_1_ progeny), Longliangyou1353 (LLY), C-Liangyou-huazhan (CLYH), Huiliangyou996 (HLY), and Huiliangyou-huazhan (HLYH), grown in expansive natural farmland planted by farmers around Nanjing in 2017. To efficiently screen the recombinant lines, a more stringent standard (plant height ≥ 150 cm) for collection of tall individuals was used here. In separated farmlands of these 4 hybrid rice varieties, a total of 24 tall F_1_ individuals (specifically, 3 individuals of HLYH, 4 individuals of LLY, 7 individuals of CLYH, and 10 individuals of HLY) were collected, and their adjacent individuals with semidwarf plant heights (3 semidwarf individuals of each tall individual) were also sampled as controls (S2B Fig).

### Identification of recombination events

Details on the canonical pattern of mitotic recombination and identification of recombination events are included in Section B, S1 Text. Markers and relevant primers used for genotyping in this study are listed in S4 and S5 Tables, respectively.

### DNA extraction and sequencing

All of the DNA samples were extracted using the cetyl trimethylammonium bromide (CTAB) method for PCR amplification, Sanger sequencing, or the WGS. All the genotyping of markers was carried out by Sanger sequencing. High-depth amplicon sequencing was performed by BGI-Shenzhen: Nonbreaking and paired-end sequencing libraries were constructed, and 2 × 150 bp reads with depth of coverage of 26,212 to 893,802 at M_9_ site were generated by Illumina Hi-seq 2500 platform (S6 Table). WGS was performed as: paired-end sequencing libraries constructed with insert size of 450 bp, and 2 × 100 bp paired-end reads were generated on Illumina Hi-Seq 4000 platform. Raw data processing, mapping, and marker identification were according to standard methods described in our previous study [7].

### Detection of the proportion of recombinant cells and nonrecombinant cells

To evaluate the pattern of emergence and expansion of recombinant cells, we also extracted DNA from different tissues of different tillers (tillers were name as t1, t2, t3, etc., and t1 is the main stem) of randomly selected LYP9 F_1_ recombinant individuals, as roots, basal leaves, and flag leaves (S5A Fig).

Based on the genotypes of 93–11 and PA64s, each marker (Fig 2) was assigned to genotype P (PA64s homozygosity), N (93–11 homozygosity), or H (heterozygosity of PA64s and 93–11), e.g., the genotype “N_8_-P_9_” means genotypes at marker 8 and 9 are, respectively, 93–11 homozygosity and PA64s homozygosity. Other genotypes are annotated in the same manner. Two key markers are markers 8 and 9 within the *SD1* gene (M_8_, M_9_), M_9_ being the C->G mutation generating the premature stop codon seen in strain 93–11, M_8_ being presence/absence of the sequence deleted in PA64. Strategically, to estimate proportions of cells that are recombinant or not in any given sample/tissue, we take advantage of the fact that 24 LYP9 plants have the recombination event at the *SD1* locus resolved as H_8_-P_9_ genotypes (namely *P*^*del*^*P*^*wt*^ / *N*^*wt*^*P*^*wt*^) (S3 Table), no matter how they were created, while nonrecombinant cells (NR-cells) are H_8_-H_9_ (namely *P*^*del*^*P*^*wt*^ / *N*^*wt*^*N*^*stop*^) genotypes (S5B Fig). If then we specifically amplify from marker 8 employing the N_8_ haplotype (namely 93–11 homozygosity at marker 8, *N*^*wt*^*N*^*stop*^ or *N*^*wt*^*P*^*wt*^), the proportion of resulting amplified haplotypes that are *N*^*stop*^ rather than *P*^*wt*^ at the 3′ M_9_ marker within this haplotype will be the proportion of nonrecombinant to recombinant cells. In other words, the ratio of recombinant cells to normal cells is exactly the ratio of PA64s genotype (C base pair) to 93–11 genotype (G base pair) at M_9_ (S5B Fig).

Based on this strategy, we devised a system of haplotype-specific nested PCR. To identify the recombinant cells and wild-type cells, the forward primer of the primerN pair is located in the 383 bp deletion and could specifically and exclusively amplify the N_8_ haplotype (i.e., haplotypes either *N*^*wt*^*N*^*stop*^ or *N*^*wt*^*P*^*wt*^), the downstream partner primer being located beyond, and not affected by, the M_9_ marker. The second round of amplification of this amplicon involved primer5, the sequence for which is contained in the amplified tract of primerN. The primer does not differentiate markers at M_9_, but the resulting amplified sequence includes, between the forward and reverse primer5 partners, the M_9_ marker site (S5B Fig). Therefore, after amplifying by the primerN pair, we perform a second (nested) amplification of the resulting amplicons employing the primer5 pair, and then sequenced the resulting fragments using a high-depth amplicon sequencing strategy. We could then resolve whether the resulting marker in any given amplified fragment at M_9_ is G (nonrecombinant) or C (recombinant). From the numbers of each, we can calculate the proportions of recombinant cells and nonrecombinant cells without recourse to error-prone single-cell analysis. Correction of sequencing error was performed by Lighter with default parameters [96]. Sequences mapping and genotype identification were according to standard methods described in our previous study [7].

### Analysis of the impact on progeny of mitotic and meiotic conversions

As we have an estimate of the event rate at this locus through meiosis [62], and can generate a comparable event rate for a given mitosis, we can compare the ratio of markers converted in and around this locus from zygote to gamete. From this, we deduce the relative impact of meiotic and mitotic conversion. We thus consider a single gamete of a plant and ask about the number of markers that have changed owing to mitotic GC since the zygote prior to the diploid meiotic cell. We compare this to the number of markers that have changed in the gamete owing to meiotic conversion.

According to the canonical pattern of mitotic recombination (S1 Fig), a conversion event can only be inherited in 1 of 2 daughter cells and 1/2 gametes, namely each mitotic conversion event has 50:50 possibility of being inherited in gametes (S7 Fig). We assigned *R*_*mi(GC)*_ as mitotic conversion rate per cell division, *L*_*mi(GC)*_ as tract length of mitotic conversion event, and *n* for number of cell divisions per generation. If there is no cell to cell competition, each division will have a 1/2*R*_*mi(GC)*_ heritable conversion rate to the next cell generation (1/2, 1/2 inheritable possibility) (S7B Fig). Thus, the expected impact on nucleotides in a pregametic cell per generation (*E*_*mi(GC)*_) could be calculated as:

$$E_{mi(GC)}=\sum_{i=1}^{n}\frac{1}{2}R_{mi(GC)}L_{mi(GC)}$$

We assigned *R*_*me(GC)*_ as meiotic conversion rate per meiosis and *L*_*me(GC)*_ as tract length of meiotic conversion event. There is only 1 meiosis per generation and a 1/4 chance that each conversion event is present in any gamete (S7 Fig). Thus, the expected impact of a meiotic conversion in a parent on nucleotides in the same randomly selected F_1_’s gamete (as considered above) per generation (*E*_*me(GC)*_) can be calculated as:

$$E_{me(GC)}=\frac{1}{4}R_{me(GC)}L_{me(GC)}$$

In our previous meiotic conversion rate estimate [62], there were 24 conversion events in 18,000 individuals, thus giving a rate as: *R*_*me(GC)*_ = ~2.66 × 10^−3^ (each individual is from 2 gametes, each gamete is from 1 meiosis, each meiotic conversion event has a 1/4 probability of being inherited in 1 gamete, 24/18000/2/(1/4) = ~2.66 × 10^−3^). For tract length of conversion (*L*_*mi(GC)*_ and *L*_*me(GC)*_), we take the average lengths of all conversion events detected in mitosis and meiosis in our data, 50297.08 bp and 9755.5 bp, respectively.

Then, the key is the evaluation of the mitotic conversion event rate per cell division (*R*_*mi(GC)*_). Based on our data giving the proportion of heterozygous cells in roots and basal leaves, we suggest that the original stem cells generating tissues (both roots and above ground parts) (namely number of original cells of root meristem (RM) and shoot apical meristem (SAM)) are at least 2 cells and that conversion events must happen before differentiation between roots and the above ground parts. Then, we simulated 4 situations as regards the original stem cells (numbers of recombinant cells are in parentheses): (1) 2 (≥1) original stem cells in RM and 2 (≥1) original stem cells in SAM; (2) 3 (≥2) original stem cells in RM and 2 (≥1) original stem cells in SAM; (3) 4 (≥2) original stem cells in RM and 4 (≥1) original stem cells in SAM; (4) 4 (≥3) original stem cells in RM and 4 (≥1) original stem cells in SAM. After simulation (details in Section C, S1 Text), *R*_*mi(GC)*_ for these 4 situations is, in order: 4.63 × 10^−6^, 8.92 × 10^−6^, 5.20 × 10^−6^, and 1.68 × 10^−5^.

To compare transmitted probability of somatic and meiotic CO events, we assigned the expected inheritable probability of somatic and meiotic CO events in a pregametic cell per generation as *P*_*mi(CO)*_ and *P*_*me(CO)*_, respectively. In view of a somatic CO event can only be inherited in 1 of 2 daughter cells and 1/2 gametes (a parallel of calculation of somatic conversion, S7 Fig), then *P*_*mi(CO)*_ could be calculated as:

$$P_{mi(CO)}=\sum_{i=1}^{n}\frac{1}{2}R_{mi(CO)}$$

In our previous study [62], we totally screened out 26 CO events related to *SD1* recovery in meiosis, then expected inheritable probability of CO events detected in F_2_s per generation, *P*_*me(CO)*_ = 26/18000/2/(1/4) = ~0.0029.

In the current study, we found a total of 19 CO events related to *SD1* recovery (18 CO events and 1 CO-GC events (Type9 in Fig 2)) in 1,100,000 LYP9 F_1_ individuals. Next, we perform a similar calculation to the above but to calculate a somatic conversion rate per cell division for 4 situations (details in Section C, S1 Text). *R*_*mi(CO)*_ for these 4 situations is 1.46 × 10^−5^, 2.82 × 10^−5^, 1.64 × 10^−5^, and 5.32 × 10^−5^. Note that in our models of early embryogenesis, there are mitotic events that happen but that we never observe so the per mitosis rates can exceed the per generation observed rates. These numbers accord with estimates from *Arabidopsis* [47] and are nearly identical to those estimated from tobacco [74].

For mitosis of diploid yeast, the expected impact on nucleotides in a pregametic cell per generation is same as that in the rice:

$$E_{mi(GC)}=\sum_{i=1}^{n}\frac{1}{2}R_{mi(GC)}L_{mi(GC)}$$

For meiosis of diploid yeast, the expected impact on nucleotides is exactly the product from number of converted markers (termed as *CM*_*me*_) and marker distance (termed as *MD*_*me*_); meanwhile, there is only 1 meiosis per generation and a 1/4 chance that each conversion event is present in any tetrad, then:

$$E_{me(GC)}=\frac{1}{4}{CM}_{me}{MD}_{me}$$

Based on previous estimations, we assume an event rate of 1.3 × 10^−6^ per mitotic division [27] and approximately 2% of markers converted per meiosis in yeast (= *CM*_*me*_) and a marker distance of approximately 227 bp (= *MD*_*me*_) [5,8].

## Supporting information

S1 Fig Processes and genetic outcomes of homologous recombination in mitosis. (A) In general, mitosis starts with replication of the parental chromosomes, but the 2 homologues do not normally associate with one another. Subsequently, after segregation and cell division, 2 daughter diploid nuclei have the same genotype as the parent. Red lines and blue lines show chromatids from 93–11 haplotypes and PA64s haplotypes, respectively. Defective alleles are marked in red. (B) In rare cases, the replicated chromosomes do associate with one another sufficiently closely for a CO or NCO-GC to occur. For CO lines, there are 2 possible orientations on the mitotic division spindle. One produces 2 heterozygous diploid daughter nuclei, but one contains the 2 parental chromatids, whereas the other receives the 2 recombinant chromatids. The alternative orientation produces 2 diploid daughter nuclei that are homozygous from the point of the CO to the end of the chromosome arm. In NCO-GC lines, there are 2 different genotypes based on the alterable conversion tract position in recombinant daughter cells. In summary, outcomes of CO events present heterozygous genotypes on one side and homozygous genotypes on another side separated by breakpoint, whereas outcomes of NCO-GC events show heterozygous genotypes on both sides of the tract but homozygous genotypes on tract. Recombinant cells with the wild-type *SD1* gene are shown with grey backgrounds, and corresponding plants are expected to present taller statures. CO, crossover; GC, gene conversion; NCO, noncrossover.
(PDF)

S2 Fig Summary of sampling of all hybrid varieties. (A) Field display of differences in plant height among hybrid individuals. A few LYP9 F_1_s were significantly taller than others. Two middle photos are taken by Prof. Dacheng Tian with tall progeny of high recombinant LYP9 individuals (left one) and normal (nonrecombinant) LYP9 F_1_ individuals (right one). (B) A total of 50 F_1_ individuals, which presented exceptionally tall statures, were sampled in summer of 2017. Specifically, 26 tall individuals from approximately 1,100,000 LYP9 individuals with plant height >130 cm were collected, and among them, 24 samples harboring the wild-type *SD1* gene (*N*^*wt*^*P*^*wt*^) (S2 Table) and 2 samples were determined to be false positives. In the other 4 crosses, a total of 24 tall individuals with plant height >150 cm were sampled, and all of them were confirmed to be recombinant lines (S6 Table). *: Lowercase “s” means sterile line, similarly for other parental lines. CLYH, C-Liangyou-huazhan; HLY, Huiliangyou996; HLYH, Huiliangyou-huazhan; LLY, Longliangyou1353; LYP9, Liang-You-Pei-Jiu.
(PDF)

S3 Fig Strategies and processes of recombination detection at the *SD1* locus. **(A)** With benefits of application of sterile line PA64s, it is easy to obtain massive numbers of hybrid progeny in cross of 2 semidwarf varieties, PA64s and 93–11. After large-scale screening of F_1_ progeny, tall individuals with plant height >130 cm were collected, together with parental lines (height: 90–110 cm) and normal F_1_s (height: 90–110 cm) as controls. **(B)** To validate that the changes of plant height derive from recombination events of *SD1* gene, 2 markers (M_8_ and M_9_) were genotyped and assigned to genotype P (PA64s homozygosity), N (93–11 homozygosity), or H (heterozygosity of PA64s and 93–11). primer4 and primer5 are used to genotype M_8_ and M_9_, respectively. As we described in the introduction, 2 semidwarf varieties, PA64s and 93–11, harbor 2 different defective alleles, here respectively denoted as P_8_-P_9_ and N_8_-N_9_. If a recombination event occurred between these 2 alleles, progeny with wild-type *SD1* gene will be generated and present tall statures, namely harboring 3 switched genotypes of M_8_ and M_9_ (N_8_-P_9_, N_8_-H_9_, H_8_-P_9_). **(C)** While the genotype of H_8_-H_9_ could be phased as P_8_-P_9_/N_8_-N_9_ or P_8_-N_9_/N_8_-P_9_, given that normal F_1_ harbors the P_8_-P_9_/N_8_-N_9_ haplotypes, but P_8_-N_9_/N_8_-P_9_ comes from a CO event, and offspring with these 2 different haplotypes are respectively named as NF_1_ (nonrecombinant F_1_) and RF_1_ (recombinant F_1_) here. To distinguish these 2 types, we employed 2 haplotype-specific primers primerP and primerN, which could specifically amplify the PA64s haplotype (P) and 93–11 haplotype (N), respectively. In detail, the forward primer of the primerP pair covered the break site of 383 bp deletion on PA64 haplotype, whereas the forward primer of primerN pair is located in the 383 bp deletion and the amplified sequence of primerN covers the M_9_ site. Expected PCR amplification results are shown in the middle, specifically, individuals with both 93–11 and PA64 haplotypes could present the combined electrophoretic bands of parental lines. After sequencing the amplification products of primer5 and primerN, NF_1_ and RF_1_ individuals show the heterozygous pattern at the M_9_ site (C/G nucleotide base, PA64/93-11 type) from amplification products of primer5 but respectively present homozygous G nucleotide base (93–11 type) and a homozygous C nucleotide base (PA64 type) at M_9_ site from amplification products of N-haplotype-specific primer primerN. **(D)** To identify the recombination types of recombinant lines, additional 13 polymorphic loci were selected as markers covering approximately 445 kb of the flanking regions around the *SD1* gene. According to broadly accepted patterns of mitotic recombination (S1), outcomes of CO events present heterozygous genotypes on one side and homozygous genotypes on the other side separated by breakpoint, whereas outcomes of NCO-GC events show heterozygous genotypes on both sides of the tract but homozygous genotypes on tract. In addition, outcomes with homozygous genotypes on both sides of the tract but homozygous genotypes on tract may come from 2 adjacent CO events or a CO event out of the *SD1* locus with a conversion event in the *SD1* gene. More complex combinations of multiple genotype switches may be caused by long-tract conversion, CO-associated conversion, or repetitive CO events. Finally, according to the different outcomes listed in right, we could identify the recombination types for tall individuals. CO, crossover; GC, gene conversion; NCO, noncrossover.
(PDF)

S4 Fig Schematic diagram for judgment of double COs and conversion associated with CO events. In mitosis, double COs and conversion associated with CO events will occur in parental cell at an extremely low frequency. To identify the recombination types, given at least the theoretical possibility of CO interference, we allowed that outcomes with homozygous genotypes on both sides of the long tracts (>100 kb) in *SD1* region may come from 2 adjacent CO events, whereas those of the short tracts may come from a CO event out of the *SD1* locus with a conversion event in the *SD1* gene. CO, crossover; GC, gene conversion.
(PDF)

S5 Fig Sample collection and haplotype-specific nested PCR assay for assessing proportions of nonrecombinant cells and recombinant cells. **(A)** We separated all tillers from each LYP9 recombinant individual and nonrecombinant individual (as control) making sure that all roots for each tiller are directly linked with their corresponding stem. Tillers were name as t1, t2, t3, etc., and t1 is the main stem which is the first stem and a little stronger and taller than other tillers. After separated tillers, for main stem, flag leaf, basal leaf, and roots were collected. Particularly, for roots, to make the collected samples were as representative as possible of the whole roots, we gathered all roots of 1 tiller into a bundle and cut a short length off them. For coleoptile tillers (t2, t3, etc.) which come out from leaf sheathes of the main stem’s basal leaves, we only collected the flag leaves and roots. **(B)** At the *SD1* locus, even harboring different kinds of recombination events, all LYP9 R-cells carry H_8_-P_9_ (namely *P*^*del*^*P*^*wt*^ / *N*^*wt*^*P*^*wt*^) genotypes (S2 Table), while NR-cells harbor H_8_-H_9_ (namely *P*^*del*^*P*^*wt*^ / *N*^*wt*^*N*^*stop*^) genotypes. So, if we specifically amplify the N_8_ haplotype (namely *N*^*wt*^*P*^*wt*^ or *N*^*wt*^*N*^*stop*^), proportions of genotypes, *N*^*stop*^ and *P*^*wt*^, on M_9_ marker of N_8_ haplotype could respectively present the proportions of NR-cells and R-cells. In other words, the ratio of R-cells to NR-cells exactly is the ratio of PA64s genotype (C base pair) to 93–11 genotype (G base pair) at M_9_. Based on this strategy (here we called it as haplotype-specific nested PCR), to identify the R-cells and NR-cells, forward primer of primerN pair is located in the 383 bp deletion, therefore it could specifically amplify the N_8_ haplotype (namely *N*^*wt*^*N*^*stop*^ or *N*^*wt*^*P*^*wt*^), and primer5 is contained in the amplified tract of primerN and covers the M_9_ marker, which can be used to identify the genotypes of M_9_ marker. Therefore, after amplifying by primerN pair and following with nested amplification by primer5 pair, using the high-depth amplicon sequencing strategy, we could calculate the proportions of R-cells and NR-cells by the numbers of reads which harbor “C” or “G” base pair at M_9_ site. LYP9, Liang-You-Pei-Jiu; NR-cells, nonrecombinant cells; R-cells, recombinant cells.
(PDF)

S6 Fig Proportions of recombinant cells and nonrecombinant cells in different tissues of LYP9 (F_1_) individuals. To evaluate the mosaic status of somatic cells, FL and R in 13 different tillers (main culm of each individual is named as “t1” and coleoptile tillers as t2, etc.) came from 9 tall individuals, randomly selected from the 24, were used to detect proportions of R-cells and NR-cells, and 3 semidwarf individuals (C1, C2, and C3) were randomly selected as controls. Based on amplicon sequencing after haplotype-specific nested PCR, we could calculate proportions of these 2 type cells (S6 Table, details in Materials and methods). In all 9 plants, all FL presented 100% R-cells and a total of 8 R of different individuals show cell heterogeneity, specifically a range of 0.01% to 13.38% of NR-cells were detected in these root tissues (data of reads number are listed in S9 Table). In addition, 5 BL from different individuals were randomly selected and detected. And all of these BL carry high ratio of R-cells and range of 0.01% to 23.90% of NR-cells, namely BL harbor mosaic somatic cells. Noticeably, a small proportion of NR-cells were detected in main culms of all these tall individuals except H17, but not in any coleoptile tillers (t2, t3, etc.), suggesting that these recombination events may occurred in early stage in SAM before engendering of LM. Tillers from each individual are shown at the bottom of figure. Blue and red bars show the proportion of R-cells and NR-cells, respectively. Underlying numerical values are presented in S2 Data. BL, basal leaf; FL, flag leaf; LM, lateral meristem; LYP9, Liang-You-Pei-Jiu; NR-cells, nonrecombinant cells; R, roots; R-cells, recombinant cells; SAM, shoot apical meristem.
(PDF)

S7 Fig Cell division and inherited pattern in gametes of mitotic and meiotic conversion. **(A)** During mitosis, a diploid cell containing a pair of homologous chromosomes (one red and one blue) undergoes DNA replication to generate sister chromatids. In the mitotic division, sister chromatids are segregated to separate daughter cells. As in mitosis, in meiosis, diploid cells containing 2 homologous chromosomes undergo DNA replication to generate sister chromatids, but with 2 divisions. In the first meiotic division, homologous chromosomes are segregated, followed by sister chromatids in the second division. If a conversion event occurs in mitosis (marked as orange star, left part of diagram), then the conversion event will be inherited in 2 of 4 gametes in the following meiosis. Whereas, if a conversion event occurs in meiosis (marked as orange star, right part of diagram), then the conversion event will be inherited in only 1 of 4 gametes. Meiosis processes and mitosis processes are showing in dotted trapezoid and dotted square, respectively. **(B)** If we only focus on the vertical line from zygote to gamete (seed to seed) and there is no cell competition between recombinant cells and nonrecombinant cells, each division will generate 1/2*R*_*mi*_ of conversion rate (1/2, 50:50 inheritable possibility; *R*_*mi*_, mitotic conversion rate per cell division).
(PDF)

S8 Fig Fine-scale dissection of recombination events in *SD1* region by WGS data of Type3 samples. Red tracts present the homozygous genotypes of pseudo parent A, yellow tracts present the heterozygous genotypes of pseudo parent A and B. The PCR marker channel shows the 15 markers used in Fig 2, and the WGS marker channel presents the markers identified by WGS data. One of them (LLY2) had poorly covered reads in this region and so was validated by PCR results of new WGS markers (S4 and S5 Tables). LLY, Longliangyou1353; WGS, whole genome sequencing.
(PDF)

S9 Fig Further consideration of relative transmission (parent to progeny) probability of meiotic and mitotic conversion events per generation, as a function of the number of cell generations from zygote to gamete. We consider a series of alternative models. First (method 1), we consider what happens if all meiotic tracts associated with 1 conversion are given the same tract length as meiotic events converting 1 marker. These can include Type 9, the event we previously excluded as it is called large but is in a region of low marker density. Second (method 2), we consider mean sum tract length per mitotic event rather than mean tract length. That is, if the sum tract length across all mitotic conversions is S, if there are N plants with identified mitotic conversion and M tracts (M>N as some plants had more than 1 conversion tract in the vicinity of SD1), then here we assume a mean conversion length (in 1 block or many) per event of S/N, rather than S/M considered previously. We adopted a similar simulation as for somatic conversion impact simulation for those 4 situations (details in Materials and methods). Methods 1 and 2 can be combined. **A**. Method 2, excluding Tract 9, low marker density estimate of meiotic tract size (equivalent to Fig 3B); **B**. Method 2, excluding Tract 9, high (WGS) marker density estimate of meiotic tract size (equivalent to Fig 3C); **C**. Method 1, including Tract 9, low marker density estimate of meiotic tract size; **D**. Method 1, including Tract 9, high marker density estimate of meiotic tract size; **E**. Method 1+2, including Tract 9, low marker density estimate of meiotic tract size; **F**. Method 1+2, including Tract 9, high marker density estimate of meiotic tract size. Underlying numerical values are presented in S2 Data. WGS, whole genome sequencing.
(PDF)

S10 Fig Relative transmission (parent to progeny) probability of meiotic and mitotic CO events per generation, as a function of the number of cell generations from zygote to gamete. To compare the transmissive probability of mitotic and meiotic CO events, we took out a similar simulation as somatic conversion impact simulation for those 4 situations (details in Materials and methods). After simulation, we find that the expected inheritable probability of somatic CO events will be at least 10% of COs detected in F_2_ individuals per generation (assuming 40 divisions per generation in plants [35]). Underlying numerical values are presented in S2 Data. CO, crossover.
(PDF)

S11 Fig Schematic of outcomes of double crossover and long noncrossover in meiosis. In meiosis, a single CO will generate 2 reciprocal rearrangement gametes (genotypes on right and left sides of the break point are from different parents), while double CO and long NCO will generate similar gametes (Gamete2 and 3 for double CO, Gamete2 for long NCO), in which genotypes of flanking sequences of the tract are from the same parents. CO, crossover; NCO, noncrossover.
(PDF)

S1 Table Summary of recombination rate and conversion tract length in prior studies in yeast, *Arabidopsis*, and tobacco. (DOCX)

S2 Table Genotypes of M_1_ to M_15_ in 24 LYP9 (F_1_) individuals. (DOCX)

S3 Table Summary statistics for whole-genome sequencing data of 12 recombinant individuals. The whole-genome sequencing data of 2 parents (93–11 and PA64s) and normal LYP9 (F_1_) are from Si and colleagues in 2015 [7]. Eight LYP9 individuals were randomly selected from 24 tall F_1_ individuals. Using the same marker (polymorphism loci) set as previous research (a total of 871,863 markers) [7], 93.68% to 98.42% heterozygous markers from 93–11 and PA64s were detected in 8 tall F_1_ individuals. Meanwhile, 90.67% to 92.85% heterozygous markers were detected in 4 recombinants from LLY cross. All these data indicate that these tall recombinants are real F_1_ generation and independent to each other. Recall rate of markers column shows the ratio of heterozygous markers from parental lines detected in F_1_ individuals. The coverage depth is calculated as the number of bases of all reads that match a genome divided by the length of the rice genome. Genome coverage shows the proportion of covered region in the whole genome. Read numbers (M) show the total numbers of each library, and mapping rate column shows percent of mapped reads out of total reads.
(DOCX)

S4 Table Markers used in identification of recombination events. (DOCX)

S5 Table Primers used in *SD1* region for genotype and recombination identification. (DOCX)

S6 Table Proportions of recombinant cells and nonrecombinant cells in different tissues of LYP9 (F_1_) individuals. (DOCX)

S7 Table Genotypes of M_1_ to M_15_ in 24 F_1_ individuals from the 4 additional crosses. (DOCX)

S8 Table Summary of recombinant individuals of LYP9 and other hybrid varieties. In summary of 2 datasets, at least 29 CO (in LYP9 lines, 18 CO; in other 4 crosses, 11 CO), 17 NCO-GC (in LYP9 lines, 3 NCO-GC; in other 4 crosses, 14 NCO-GC), and 7 CO-GC (in LYP9 lines, 3 CO-GC; in other 4 crosses, 4 CO-GC) events had occurred in 48 tall individuals. There are 2 adjacent NCO-GC events and 2 adjacent CO events that occurred in recombination Type3 and Type6, respectively.
(DOCX)

S9 Table Inheritance of tall phenotype and wild-type *SD1* gene of F_2_ progeny of three randomly selected tall LYP9 individuals. To evaluate heritability of these recombinant cells, 43 F_2_ progeny of 3 randomly selected tall individuals (H1, H8, and H14) were genotyped at the *SD1* locus. Part of the progeny was tall with at least 130 cm (H_8_-P_9_) to 170 cm (N_8_-P_9_), and other progeny which present the same *SD1* genotypes as their parents (N_8_-P_9_) were around 90–110 cm.
(DOCX)

S10 Table Individual distribution of *SD1* alleles for progeny of H1, H8, and H14. All these 3 F_1_ individuals have the same genotype (H_8_-P_9_) at markers 8 and 9, i.e., *P*^*del*^*P*^*wt*^ / *N*^*wt*^*P*^*wt*^, thus their progeny are expected to harbor *N*^*wt*^*P*^*wt*^ / *N*^*wt*^*P*^*wt*^, *P*^*del*^*P*^*wt*^ / *N*^*wt*^*P*^*wt*^, and *P*^*del*^*P*^*wt*^ / *P*^*del*^*P*^*wt*^, 3 kinds of alleles. After *χ*^*2*^ test, we found that the allele frequency for progeny of each F_1_ individual followed the segregation law, also indicating that all mitotic recombination events are well transmitted into their progeny.
(DOCX)

S11 Table List of close double CO events (distance between 10 kb and 20 kb) identified in yeast tetrads. A total of 105 close double CO events (distance between 10 kb and 20 kb) were identified in 29 yeast tetrads. Among them, distances of 24 close double CO events are shorter than 12 kb. Break points of CO events are judged by midpoint method. Prebreak line and postbreak line are break points of front CO and break points of subsequent CO. Data from Liu and colleagues [8].
(DOCX)

S1 Text Supporting information about identification of recombination events and simulation of mitotic recombination rate. (A). The canonical pattern of mitotic recombination. (B). Identification of recombination events. (C). Simulation of conversion and crossover rate per mitosis in different situations.
(DOCX)

S1 Data Specific markers detected in 4 LLY F_1_ individuals. (XLSX)

S2 Data All underlying raw data used to generate manuscript figures. (XLSX)

## Abbreviations

BIR: break-induced replication
CLYH: C-Liangyou-huazhan
CO: crossover
CTAB: cetyl trimethylammonium bromide
DSB: double-strand break
DSBR: DSB repair
GC: gene conversion
GUS: β‐glucuronidase
HLY: Huiliangyou996
HLYH: Huiliangyou-huazhan
HR: homologous recombination
IVF: in vitro fertilization
JAAS: Jiangsu Academy of Agricultural Sciences
LD: linkage disequilibrium
LLY: Longliangyou1353
LOH: loss-of-heterozygosity
LUC: luciferase
LYP9: Liang-You-Pei-Jiu
NCO: noncrossover
RCO: reciprocal crossover
RM: root meristem
SAM: shoot apical meristem
SDSA: synthesis-dependent strand annealing
WGS: whole genome sequencing
