# Supplementary material for: Mitotic gene conversion can be as important as meiotic conversion in driving genetic variability in plants and other species without early germline segregation
Source: PLoS Biol. 2021 Mar 22;19(3):e3001164. doi: 10.1371/journal.pbio.3001164 (PMC8016264; doi:10.1371/journal.pbio.3001164)
Supplement: S1 Fig — (A) In general, mitosis starts with replication of the parental chromosomes, but the 2 homologues do not normally associate with one another. Subsequently, after segregation and cell division, 2 daughter diploid nuclei have the same genotype as the parent. Red lines and blue lines show chromatids from 93–11 haplotypes and PA64s haplotypes, respectively. Defective alleles are marked in red. (B) In rare cases, the replicated chromosomes do associate with one another sufficiently closely for a CO or NCO-GC to occur. For CO lines, there are 2 possible orientations on the mitotic division spindle. One produces 2 heterozygous diploid daughter nuclei, but one contains the 2 parental chromatids, whereas the other receives the 2 recombinant chromatids. The alternative orientation produces 2 diploid daughter nuclei that are homozygous from the point of the CO to the end of the chromosome arm. In NCO-GC lines, there are 2 different genotypes based on the alterable conversion tract position in recombinant daughter cells. In summary, outcomes of CO events present heterozygous genotypes on one side and homozygous genotypes on another side separated by breakpoint, whereas outcomes of NCO-GC events show heterozygous genotypes on both sides of the tract but homozygous genotypes on tract. Recombinant cells with the wild-type SD1 gene are shown with grey backgrounds, and corresponding plants are expected to present taller statures. CO, crossover; GC, gene conversion; NCO, noncrossover. (PDF) [file pbio.3001164.s001.pdf]

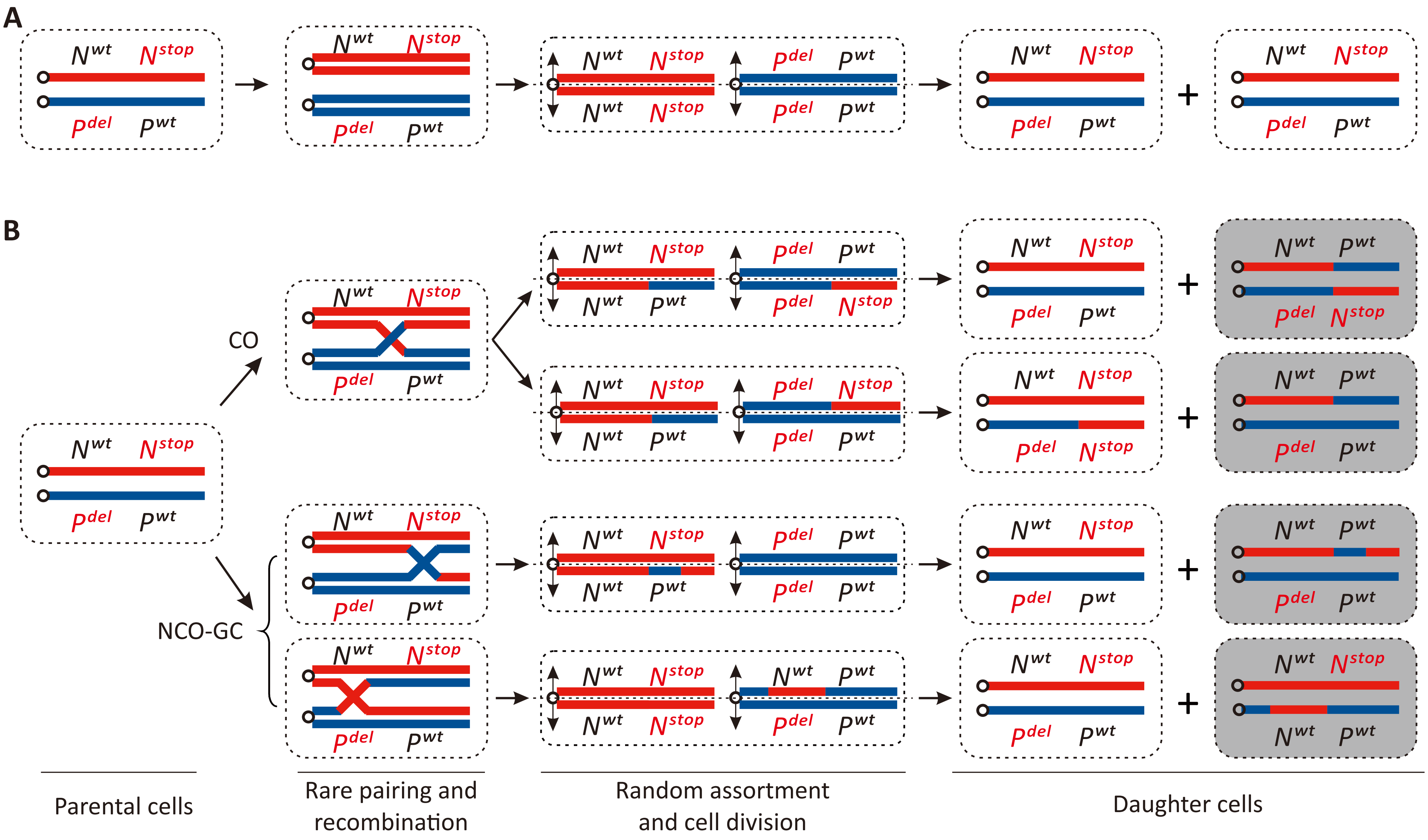

S1 Fig. Processes and genetic outcomes of homologous recombination in mitosis.

(A) In general, mitosis starts with replication of the parental chromosomes, but the two homologues do not normally associate with one another. Subsequently, after segregation and cell division, two daughter diploid nuclei have the same genotype as the parent. Red lines and blue lines show chromatids from 93-11 haplotypes and PA64s haplotypes, respectively. Defective alleles are marked in red.

(B) In rare cases, the replicated chromosomes do associate with one another sufficiently closely for a crossover (CO) or non-crossover associated conversion (NCO-GC) to occur. For CO lines, there are two possible orientations on the mitotic division spindle. One produces two heterozygous diploid daughter nuclei, but one contains the two parental chromatids, whereas the other receives the two recombinant chromatids. The alternative orientation produces two diploid daughter nuclei that are homozygous from the point of the crossover to the end of the chromosome arm. In NCO-GC lines, there are two different genotypes based on the alterable conversion tract position in recombinant daughter cells. In summary, outcomes of CO events present heterozygous genotypes on one side and homozygous genotypes on another side separated by breakpoint, whereas outcomes of NCO-GC events show heterozygous genotypes on both sides of the tract but homozygous genotypes on tract. Recombinant cells with the wild-type *SD1* gene are shown with grey backgrounds, and corresponding plants are expected to present taller statures.
