# Supplementary material for: Mitotic gene conversion can be as important as meiotic conversion in driving genetic variability in plants and other species without early germline segregation
Source: PLoS Biol. 2021 Mar 22;19(3):e3001164. doi: 10.1371/journal.pbio.3001164 (PMC8016264; doi:10.1371/journal.pbio.3001164)
Supplement: S2 Fig — (A) Field display of differences in plant height among hybrid individuals. A few LYP9 F1s were significantly taller than others. Two middle photos are taken by Prof. Dacheng Tian with tall progeny of high recombinant LYP9 individuals (left one) and normal (nonrecombinant) LYP9 F1 individuals (right one). (B) A total of 50 F1 individuals, which presented exceptionally tall statures, were sampled in summer of 2017. Specifically, 26 tall individuals from approximately 1,100,000 LYP9 individuals with plant height >130 cm were collected, and among them, 24 samples harboring the wild-type SD1 gene (NwtPwt) (S2 Table) and 2 samples were determined to be false positives. In the other 4 crosses, a total of 24 tall individuals with plant height >150 cm were sampled, and all of them were confirmed to be recombinant lines (S6 Table). *: Lowercase “s” means sterile line, similarly for other parental lines. CLYH, C-Liangyou-huazhan; HLY, Huiliangyou996; HLYH, Huiliangyou-huazhan; LLY, Longliangyou1353; LYP9, Liang-You-Pei-Jiu. (PDF) [file pbio.3001164.s002.pdf]

A

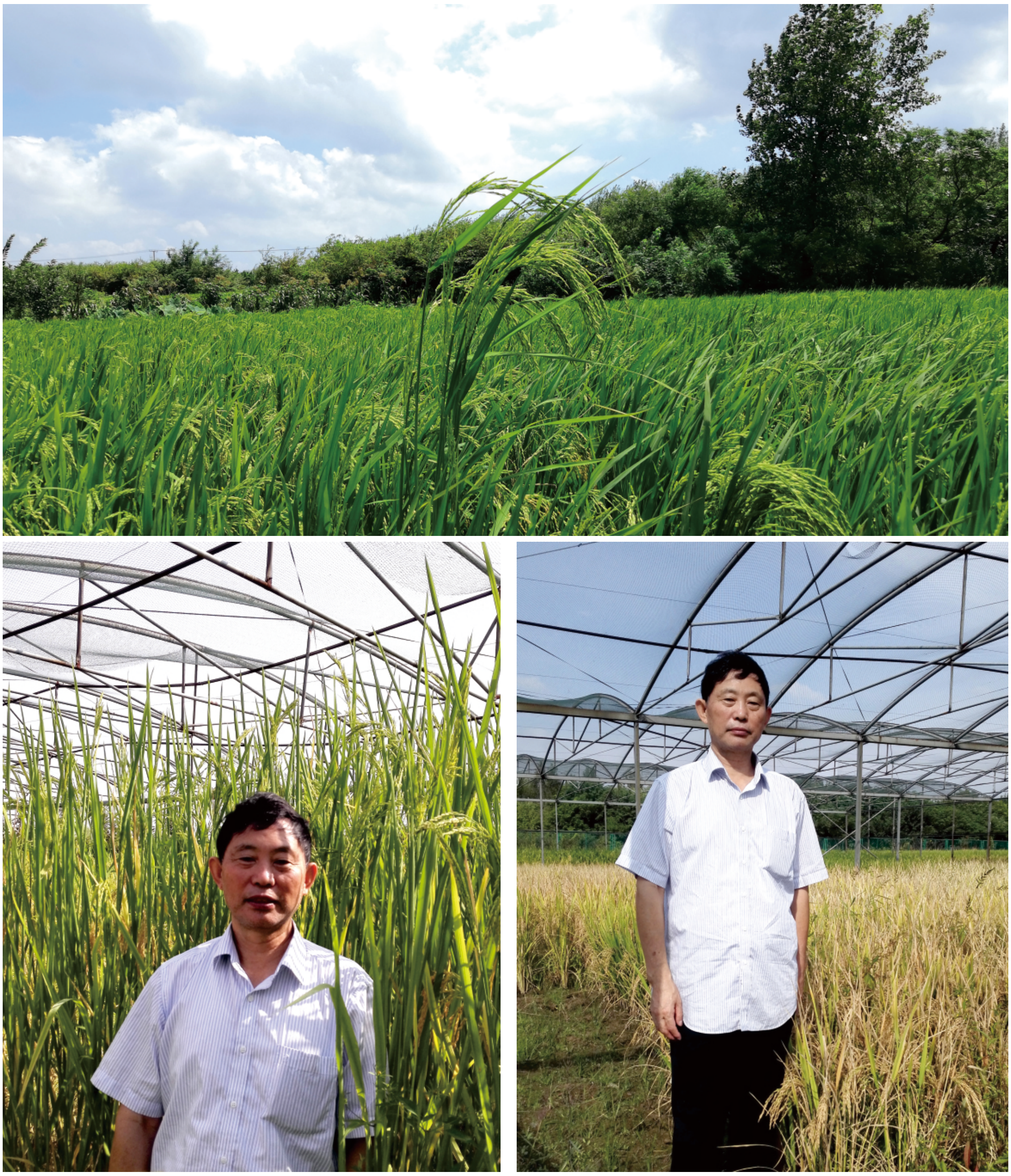

B

Hybrid variety	Symbol	Female parent	*SD1* allele	Male parent	*SD1* allele	Samples
Liangyoupei9	LYP9	PA64s*	$P^{del}P^{wt}$	93-11	$N^{wt}N^{stop}$	26
Longliangyou1353	LLY	Longke638s	$P^{del}P^{wt}$	R1353	$N^{wt}N^{stop}$	4
C-Liangyou-huazhan	CLYH	C815s	$P^{del}P^{wt}$	Huazhan	$N^{wt}N^{stop}$	7
Huiliangyou996	HLY	1892s	$P^{del}P^{wt}$	R996	$N^{wt}N^{stop}$	10
Huiliangyou-huazhan	HLYH	Longke638s	$P^{del}P^{wt}$	Huazhan	$N^{wt}N^{stop}$	3

S2 Fig. Summary of sampling of all hybrid varieties.

(A) Field display of differences in plant height among hybrid individuals. A few LYP9 F_1s were significantly taller than others. Two middle photos are taken by Prof. Dacheng Tian with tall progeny

of high recombinant LYP9 individuals (left one) and normal (non-recombinant) LYP9 F_1 individuals (right one).

(B) A total of 50 F_1 individuals which presented exceptionally tall statures, were sampled in summer of 2017. Specifically, 26 tall individuals from ~1,100,000 LYP9 individuals with plant height > 130 cm were collected, and among them, 24 samples harboring the wild-type *SD1* gene ($N^{wt}P^{wt}$) (Supplementary table S2) and two samples were determined to be false positives. In the other four crosses, a total of 24 tall individuals with plant height > 150 cm were sampled, and all of them were confirmed to be recombinant lines (Supplementary table S6). *: Lowercase "s" means sterile line, similarly for other parental lines.
