# Supplementary material for: Mitotic gene conversion can be as important as meiotic conversion in driving genetic variability in plants and other species without early germline segregation
Source: PLoS Biol. 2021 Mar 22;19(3):e3001164. doi: 10.1371/journal.pbio.3001164 (PMC8016264; doi:10.1371/journal.pbio.3001164)
Supplement: S3 Fig — (A) With benefits of application of sterile line PA64s, it is easy to obtain massive numbers of hybrid progeny in cross of 2 semidwarf varieties, PA64s and 93–11. After large-scale screening of F1 progeny, tall individuals with plant height >130 cm were collected, together with parental lines (height: 90–110 cm) and normal F1s (height: 90–110 cm) as controls. (B) To validate that the changes of plant height derive from recombination events of SD1 gene, 2 markers (M8 and M9) were genotyped and assigned to genotype P (PA64s homozygosity), N (93–11 homozygosity), or H (heterozygosity of PA64s and 93–11). primer4 and primer5 are used to genotype M8 and M9, respectively. As we described in the introduction, 2 semidwarf varieties, PA64s and 93–11, harbor 2 different defective alleles, here respectively denoted as P8-P9 and N8-N9. If a recombination event occurred between these 2 alleles, progeny with wild-type SD1 gene will be generated and present tall statures, namely harboring 3 switched genotypes of M8 and M9 (N8-P9, N8-H9, H8-P9). (C) While the genotype of H8-H9 could be phased as P8-P9/N8-N9 or P8-N9/N8-P9, given that normal F1 harbors the P8-P9/N8-N9 haplotypes, but P8-N9/N8-P9 comes from a CO event, and offspring with these 2 different haplotypes are respectively named as NF1 (nonrecombinant F1) and RF1 (recombinant F1) here. To distinguish these 2 types, we employed 2 haplotype-specific primers primerP and primerN, which could specifically amplify the PA64s haplotype (P) and 93–11 haplotype (N), respectively. In detail, the forward primer of the primerP pair covered the break site of 383 bp deletion on PA64 haplotype, whereas the forward primer of primerN pair is located in the 383 bp deletion and the amplified sequence of primerN covers the M9 site. Expected PCR amplification results are shown in the middle, specifically, individuals with both 93–11 and PA64 haplotypes could present the combined electrophoretic bands of parental lines. After sequencing the ampli [file pbio.3001164.s003.pdf]

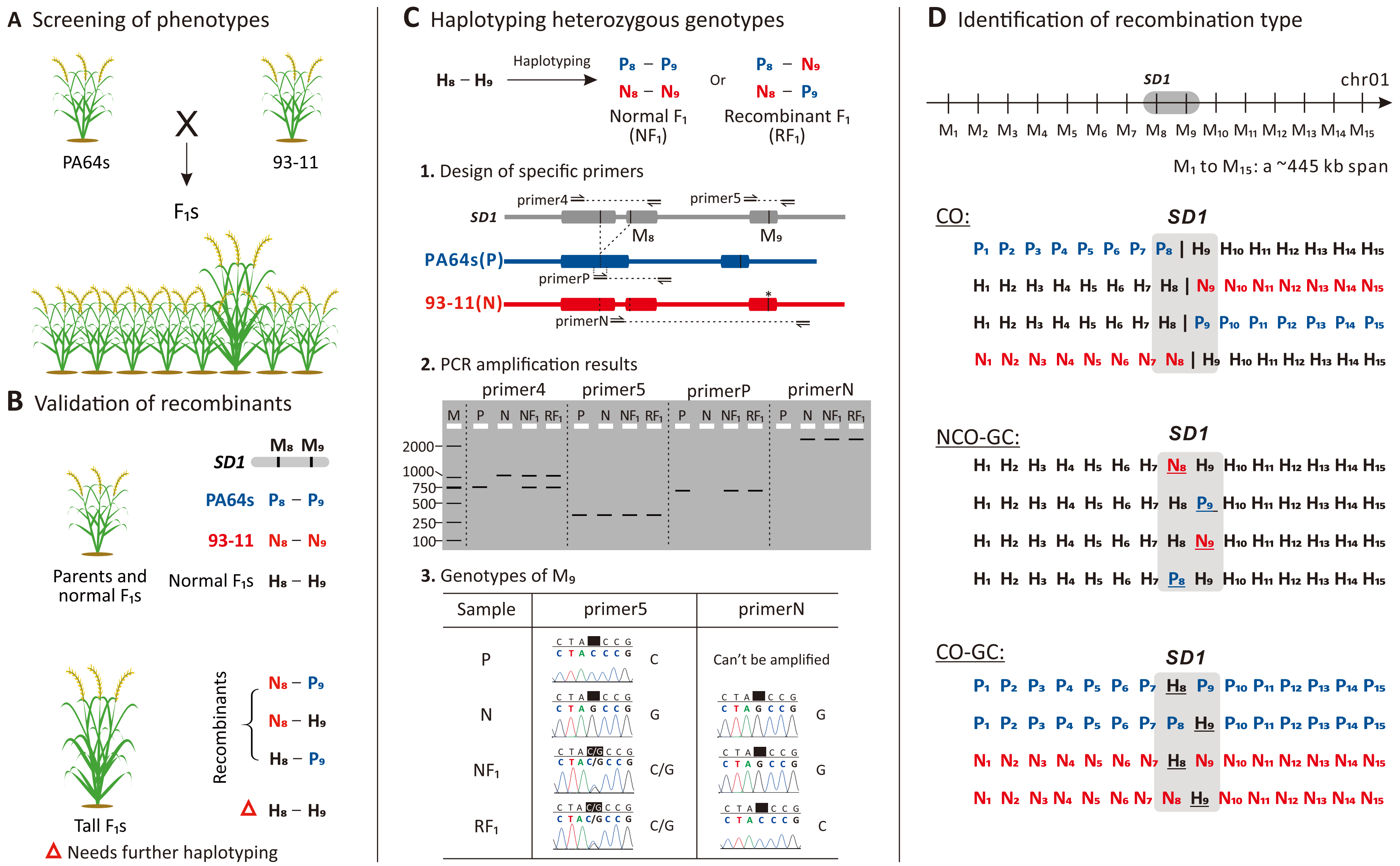
A Screening of phenotypes
PA64s
93-11
X
F1s
B Validation of recombinants
SD1
M8 M9
PA64s P8 – P9
93-11 N8 – N9
Parents and normal F1s
Normal F1s H8 – H9
Recombinants
N8 – P9
N8 – H9
H8 – P9
H8 – H9
Tall F1s
Needs further haplotyping
C Haplotyping heterozygous genotypes
H8 – H9
Haplotyping
P8 – P9
N8 – N9
Normal F1 (NF1)
Or
P8 – N9
N8 – P9
Recombinant F1 (RF1)
1. Design of specific primers
SD1
primer4
primer5
M8
M9
PA64s(P)
primerP
93-11(N)
primerN
2. PCR amplification results
primer4
primer5
primerP
primerN
M P N NF1 RF1 P N NF1 RF1 P N NF1 RF1 P N NF1 RF1
2000
1000
750
500
250
100
3. Genotypes of M9
Sample
primer5
primerN
P
N
NF1
RF1
C
G
C/G
C/G
Can't be amplified
G
G
C
D Identification of recombination type
SD1
chr01
M1 M2 M3 M4 M5 M6 M7 M8 M9 M10 M11 M12 M13 M14 M15
M1 to M15: a ~445 kb span
CO:
SD1
P1 P2 P3 P4 P5 P6 P7 P8 | H9 H10 H11 H12 H13 H14 H15
H1 H2 H3 H4 H5 H6 H7 H8 | N9 N10 N11 N12 N13 N14 N15
H1 H2 H3 H4 H5 H6 H7 H8 | P9 P10 P11 P12 P13 P14 P15
N1 N2 N3 N4 N5 N6 N7 N8 | H9 H10 H11 H12 H13 H14 H15
NCO-GC:
SD1
H1 H2 H3 H4 H5 H6 H7 N8 H9 H10 H11 H12 H13 H14 H15
H1 H2 H3 H4 H5 H6 H7 H8 P9 H10 H11 H12 H13 H14 H15
H1 H2 H3 H4 H5 H6 H7 H8 N9 H10 H11 H12 H13 H14 H15
H1 H2 H3 H4 H5 H6 H7 P8 H9 H10 H11 H12 H13 H14 H15
CO-GC:
SD1
P1 P2 P3 P4 P5 P6 P7 H8 P9 P10 P11 P12 P13 P14 P15
P1 P2 P3 P4 P5 P6 P7 P8 H9 P10 P11 P12 P13 P14 P15
N1 N2 N3 N4 N5 N6 N7 H8 N9 N10 N11 N12 N13 N14 N15
N1 N2 N3 N4 N5 N6 N7 N8 H9 N10 N11 N12 N13 N14 N15

S3 Fig. Strategies and processes of recombination detection at the *SD1* locus.

(A) With benefits of application of sterile line PA64s, it is easy to obtain massive numbers of hybrid progeny in cross of two semi-dwarf varieties, PA64s and 93-11. After large-scale screening of F_1 progeny, tall individuals with plant height > 130cm were collected, together with parental lines (height: 90-110cm) and normal F_1s (height: 90-110cm) as controls.

(B) To validate that the changes of plant height derive from recombination events of *SD1* gene, two markers (M_8 and M_9) were genotyped and assigned to genotype P (PA64s homozygosity), N (93-11 homozygosity) or H (heterozygosity of PA64s and 93-11). primer4 and primer5 are used to genotype M_8 and M_9, respectively. As we described in the introduction, two semi-dwarf varieties, PA64s and 93-11, harbor two different defective alleles, here respectively denoted as P_8-P_9 and N_8-N_9. If a recombination event occurred between these two alleles, progeny with wild-type *SD1* gene will be generated and present tall statures, namely harboring three switched genotypes of M_8 and M_9 (N_8-P_9, N_8-H_9, H_8-P_9).

(C) While the genotype of H_8-H_9 could be phased as P_8-P_9/N_8-N_9 or P_8-N_9/N_8-P_9, given that normal F_1 harbors the P_8-P_9/N_8-N_9 haplotypes, but P_8-N_9/N_8-P_9 comes from a crossover event, and offspring with these two different haplotypes are respectively named as NF_1 (non-recombinant F_1) and RF_1 (recombinant F_1) here. To distinguish these two types, we employed two haplotype-specific primers primerP and primerN, which could specifically amplify the PA64s haplotype (P) and 93-11 haplotype (N), respectively. In detail, the forward primer of the primerP pair covered the break site of 383bp deletion on PA64 haplotype, whereas the forward primer of primerN pair is located in the 383 bp deletion and the amplified sequence of primerN covers the M_9 site. Expected PCR amplification results are shown in the middle, specifically, individuals with both 93-11 and PA64 haplotypes could present the combined electrophoretic bands of parental lines. After sequencing the amplification products of primer5 and primerN, NF_1 and RF_1 individuals show the heterozygous pattern at the M_9 site (C/G nucleotide base, PA64/93-11 type) from amplification products of primer5, but respectively present homozygous G nucleotide base (93-11 type) and a homozygous C nucleotide base (PA64 type) at M_9 site from amplification products of N-haplotype-specific primer primerN.

(D) To identify the recombination types of recombinant lines, additional 13 polymorphic loci were selected as markers covering approximately 445 kb of the flanking regions around the *SD1* gene. According to broadly accepted patterns of mitotic recombination (S1 Fig.), outcomes of CO events present heterozygous genotypes on one side and homozygous genotypes on the other side separated by breakpoint, whereas outcomes of NCO-GC events show heterozygous genotypes on both sides of the tract but homozygous genotypes on tract. In addition, outcomes with homozygous genotypes on both sides of the tract but homozygous genotypes on tract may come from two adjacent CO events or a CO event out of the *SD1* locus with a conversion event in the *SD1* gene. More complex combinations of multiple genotype switches may be caused by long-tract conversion, CO-associated conversion or repetitive CO events. Finally, according to the different outcomes listed in right, we could identify the recombination types for tall individuals.
