# Supplementary material for: Mitotic gene conversion can be as important as meiotic conversion in driving genetic variability in plants and other species without early germline segregation
Source: PLoS Biol. 2021 Mar 22;19(3):e3001164. doi: 10.1371/journal.pbio.3001164 (PMC8016264; doi:10.1371/journal.pbio.3001164)
Supplement: S4 Fig — In mitosis, double COs and conversion associated with CO events will occur in parental cell at an extremely low frequency. To identify the recombination types, given at least the theoretical possibility of CO interference, we allowed that outcomes with homozygous genotypes on both sides of the long tracts (>100 kb) in SD1 region may come from 2 adjacent CO events, whereas those of the short tracts may come from a CO event out of the SD1 locus with a conversion event in the SD1 gene. CO, crossover; GC, gene conversion. (PDF) [file pbio.3001164.s004.pdf]

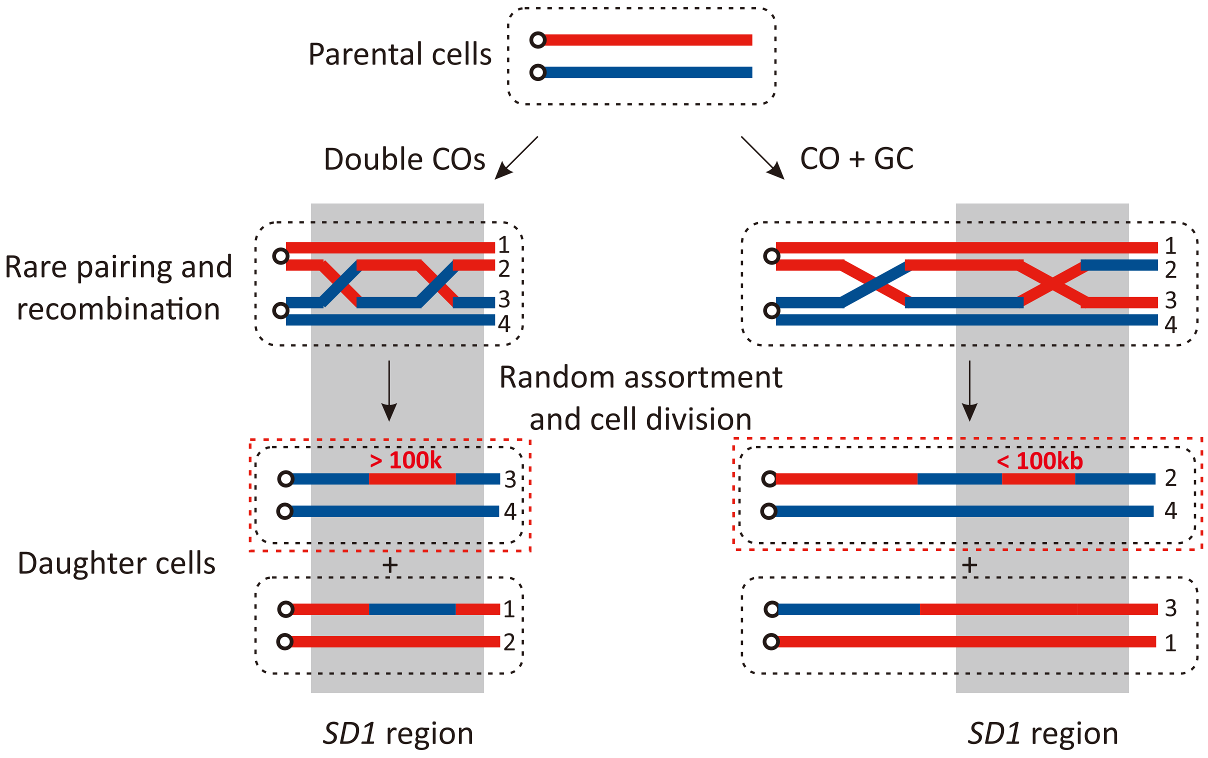

S4 Fig. Schematic diagram for judgment of double-COs and conversion associated with CO events.

In mitosis, double COs and conversion associated with CO events will occur in parental cell at a extremely low frequency. To identify the recombination types, given at least the theoretical possibility of crossover interference, we allowed that outcomes with homozygous genotypes on both sides of the long tracts (>100 kb) in *SD1* region may come from two adjacent CO events, whereas those of the short tracts may come from a CO event out of the *SD1* locus with a conversion event in the *SD1* gene.
