# Supplementary material for: Mitotic gene conversion can be as important as meiotic conversion in driving genetic variability in plants and other species without early germline segregation
Source: PLoS Biol. 2021 Mar 22;19(3):e3001164. doi: 10.1371/journal.pbio.3001164 (PMC8016264; doi:10.1371/journal.pbio.3001164)
Supplement: S5 Fig — (A) We separated all tillers from each LYP9 recombinant individual and nonrecombinant individual (as control) making sure that all roots for each tiller are directly linked with their corresponding stem. Tillers were name as t1, t2, t3, etc., and t1 is the main stem which is the first stem and a little stronger and taller than other tillers. After separated tillers, for main stem, flag leaf, basal leaf, and roots were collected. Particularly, for roots, to make the collected samples were as representative as possible of the whole roots, we gathered all roots of 1 tiller into a bundle and cut a short length off them. For coleoptile tillers (t2, t3, etc.) which come out from leaf sheathes of the main stem’s basal leaves, we only collected the flag leaves and roots. (B) At the SD1 locus, even harboring different kinds of recombination events, all LYP9 R-cells carry H8-P9 (namely PdelPwt / NwtPwt) genotypes (S2 Table), while NR-cells harbor H8-H9 (namely PdelPwt / NwtNstop) genotypes. So, if we specifically amplify the N8 haplotype (namely NwtPwt or NwtNstop), proportions of genotypes, Nstop and Pwt, on M9 marker of N8 haplotype could respectively present the proportions of NR-cells and R-cells. In other words, the ratio of R-cells to NR-cells exactly is the ratio of PA64s genotype (C base pair) to 93–11 genotype (G base pair) at M9. Based on this strategy (here we called it as haplotype-specific nested PCR), to identify the R-cells and NR-cells, forward primer of primerN pair is located in the 383 bp deletion, therefore it could specifically amplify the N8 haplotype (namely NwtNstop or NwtPwt), and primer5 is contained in the amplified tract of primerN and covers the M9 marker, which can be used to identify the genotypes of M9 marker. Therefore, after amplifying by primerN pair and following with nested amplification by primer5 pair, using the high-depth amplicon sequencing strategy, we could calculate the proportions of R-cells and NR-cells by the numbers of reads whi [file pbio.3001164.s005.pdf]

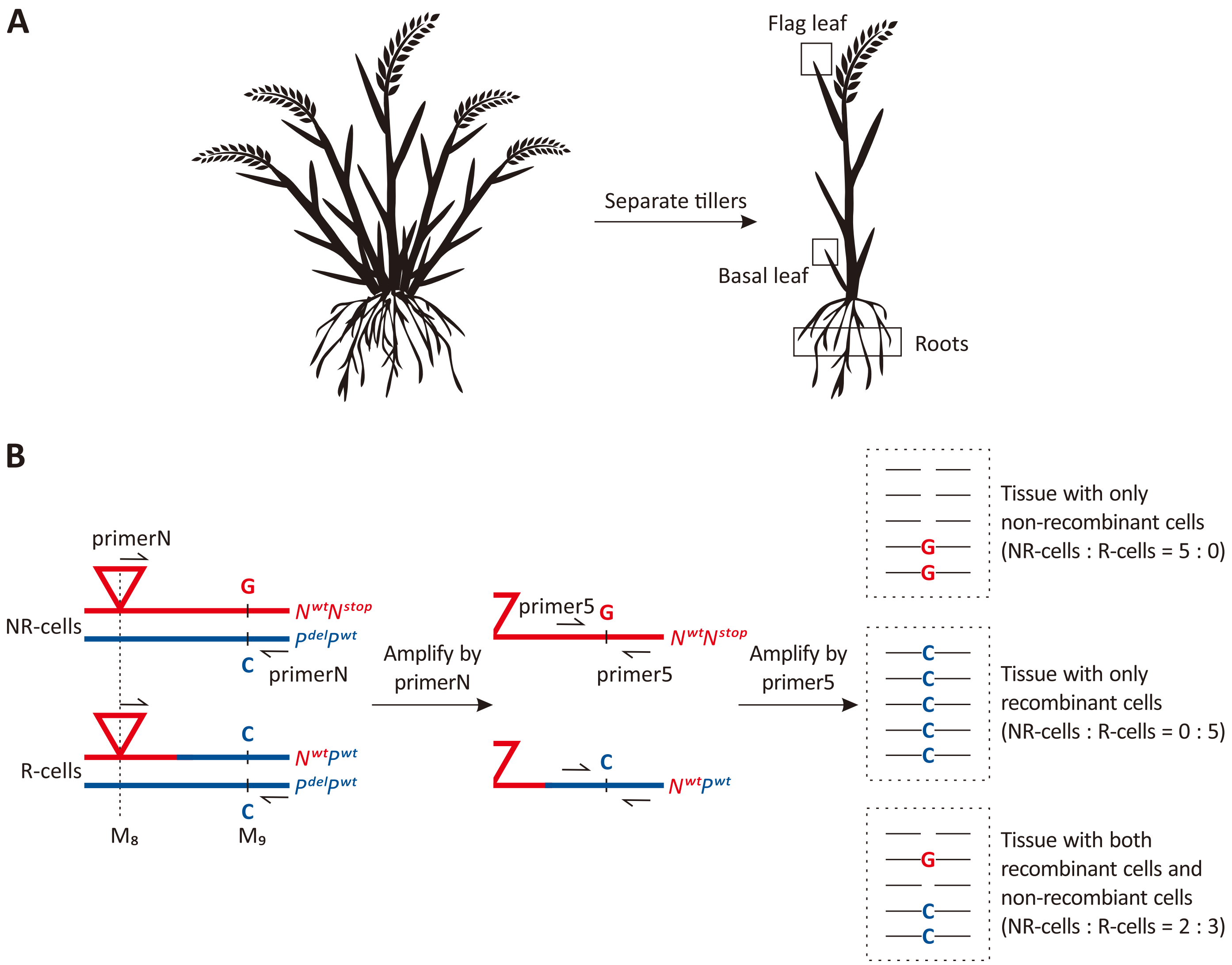

S5 Fig. Sample collection and haplotype-specific nested PCR assay for assessing proportions of non-recombinant cells and recombinant cells.

(A) We separated all tillers from each LYP9 recombinant individual and non-recombinant individual (as control) making sure that all roots for each tiller are directly linked with their corresponding stem. Tillers were name as t1, t2, t3, etc. and t1 is the main stem which is the first stem and a little stronger and taller than other tillers. After separated tillers, for main stem, flag leaf, basal leaf and roots were collected. Particularly, for roots, to make the collected samples were as representative as possible of the whole roots, we gathered all roots of one tiller into a bundle and cut a short length off them. For coleoptile tillers (t2, t3, etc.) which come out from leaf sheathes of the main stem's basal leaves, we only collected the flag leaves and roots.

(B) At the *SD1* locus, even harboring different kinds of recombination events, all LYP9 recombinant cells (R-cells) carry H_8-P_9 (namely $P^{del}P^{wt}$ / $N^{wt}P^{wt}$) genotypes (Supplementary table S2), while non-recombinant cells (NR-cells) harbor H_8-H_9 (namely $P^{del}P^{wt}$ / $N^{wt}N^{stop}$) genotypes. So, if we specifically amplify the N_8 haplotype (namely $N^{wt}P^{wt}$ or $N^{wt}N^{stop}$), proportions of genotypes, N^{stop} and P^{wt}, on M_9 marker of N_8 haplotype could respectively present the proportions of non-recombinant cells (NR-cells) and recombinant cells (R-cells). In other words, the ratio of recombinant cells to non-recombinant cells exactly is the ratio of PA64s genotype (C base pair) to 93-11 genotype (G base pair) at M_9. Based on this strategy (here we called it as haplotype-specific

nested PCR), to identify the recombinant cells and non-recombinant cells, forward primer of primerN pair is located in the 383 bp deletion, therefore it could specifically amplify the N_8 haplotype (namely $N^{wt}N^{stop}$ or $N^{wt}P^{wt}$), and primer5 is contained in the amplified tract of primerN and covers the M_9 marker, which can be used to identify the genotypes of M_9 marker. Therefore, after amplifying by primerN pair and following with nested amplification by primer5 pair, using the high-depth amplicon sequencing strategy, we could calculate the proportions of recombinant cells and non-recombinant cells by the numbers of reads which harbor “C” or “G” base pair at M_9 site.
