# Supplementary material for: Mitotic gene conversion can be as important as meiotic conversion in driving genetic variability in plants and other species without early germline segregation
Source: PLoS Biol. 2021 Mar 22;19(3):e3001164. doi: 10.1371/journal.pbio.3001164 (PMC8016264; doi:10.1371/journal.pbio.3001164)
Supplement: S6 Fig — To evaluate the mosaic status of somatic cells, FL and R in 13 different tillers (main culm of each individual is named as “t1” and coleoptile tillers as t2, etc.) came from 9 tall individuals, randomly selected from the 24, were used to detect proportions of R-cells and NR-cells, and 3 semidwarf individuals (C1, C2, and C3) were randomly selected as controls. Based on amplicon sequencing after haplotype-specific nested PCR, we could calculate proportions of these 2 type cells (S6 Table, details in Materials and methods). In all 9 plants, all FL presented 100% R-cells and a total of 8 R of different individuals show cell heterogeneity, specifically a range of 0.01% to 13.38% of NR-cells were detected in these root tissues (data of reads number are listed in S9 Table). In addition, 5 BL from different individuals were randomly selected and detected. And all of these BL carry high ratio of R-cells and range of 0.01% to 23.90% of NR-cells, namely BL harbor mosaic somatic cells. Noticeably, a small proportion of NR-cells were detected in main culms of all these tall individuals except H17, but not in any coleoptile tillers (t2, t3, etc.), suggesting that these recombination events may occurred in early stage in SAM before engendering of LM. Tillers from each individual are shown at the bottom of figure. Blue and red bars show the proportion of R-cells and NR-cells, respectively. Underlying numerical values are presented in S2 Data. BL, basal leaf; FL, flag leaf; LM, lateral meristem; LYP9, Liang-You-Pei-Jiu; NR-cells, nonrecombinant cells; R, roots; R-cells, recombinant cells; SAM, shoot apical meristem. (PDF) [file pbio.3001164.s006.pdf]

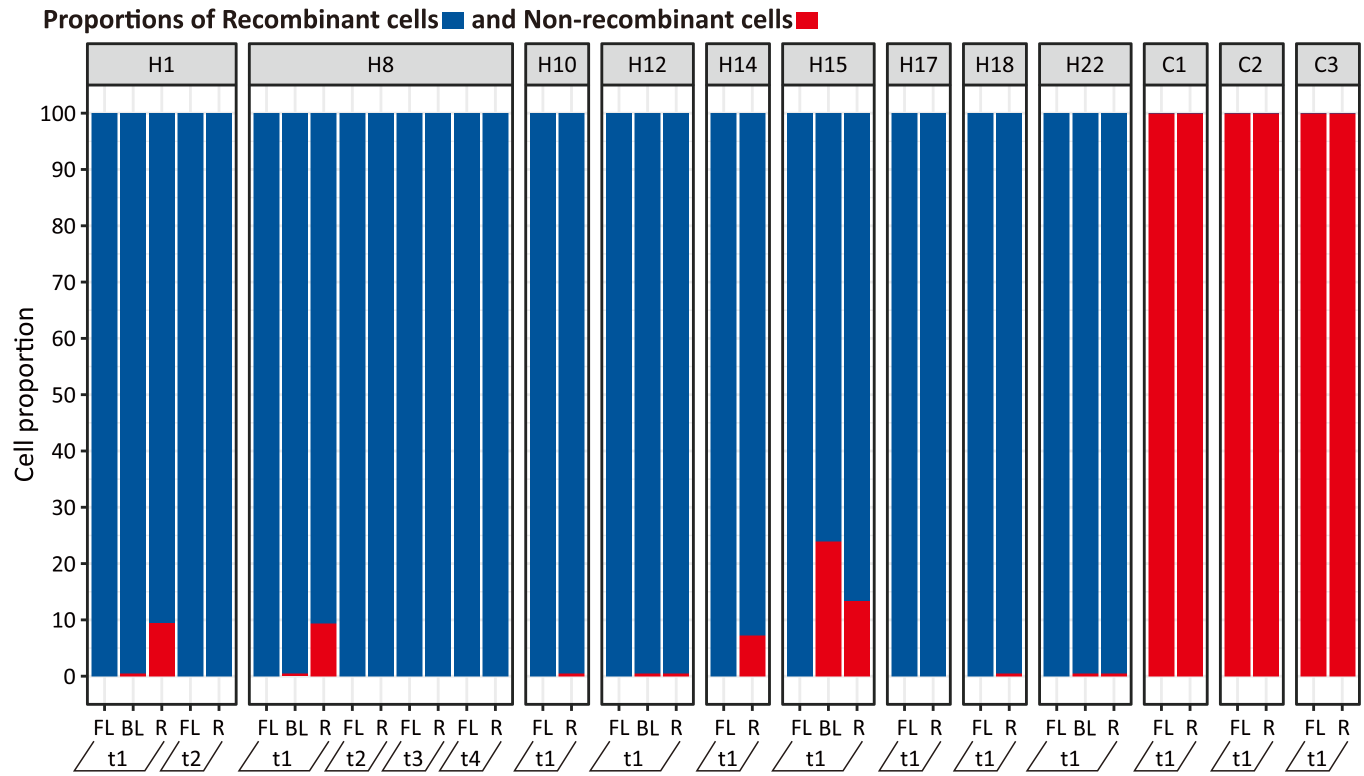

S6 Fig. Proportions of recombinant cells and non-recombinant cells in different tissues of LYP9 (F_1) individuals.

To evaluate the mosaic status of somatic cells, flag leaves (FL) and roots (R) in 13 different tillers (main culm of each individual is named as "t1" and coleoptile tillers as t2, etc.) came from nine tall individuals, randomly selected from the 24, were used to detect proportions of recombinant cells (R-cells) and non-recombinant cells (NR-cells), and three semi-dwarf individuals (C1, C2 and C3) were randomly selected as controls. Based on amplicon sequencing after haplotype-specific nested PCR, we could calculate proportions of these two type cells (S5, details in Materials and Methods). In all nine plants, all flag leaves presented 100% R-cells and a total of 8 roots of different individuals show cell heterogeneity, specifically a range of 0.01% to 13.38% of NR-cells were detected in these root tissues (data of reads number are listed in Supplementary table S9). In addition, five basal leaves (BL) from different individuals were randomly selected and detected. And all of these basal leaves carry high ratio of R-cells and range of 0.01% to 23.90% of NR-cells, namely basal leaves harbor mosaic somatic cells. Noticeably, a small proportion of non-recombinant cells were detected in main culms of all these tall individuals except H17, but not in any coleoptile tillers (t2, t3, etc.), suggesting that these recombination events may occurred in early stage in shoot apical meristem (SAM) before engendering of lateral meristem (LM). Tillers from each individual are shown at the bottom of figure. FL, flag leaf; BL, basal leaf; R, roots. Blue and red bars show the proportion of recombinant cells and non-recombinant cells, respectively.
