# Supplementary material for: Mitotic gene conversion can be as important as meiotic conversion in driving genetic variability in plants and other species without early germline segregation
Source: PLoS Biol. 2021 Mar 22;19(3):e3001164. doi: 10.1371/journal.pbio.3001164 (PMC8016264; doi:10.1371/journal.pbio.3001164)
Supplement: S7 Fig — (A) During mitosis, a diploid cell containing a pair of homologous chromosomes (one red and one blue) undergoes DNA replication to generate sister chromatids. In the mitotic division, sister chromatids are segregated to separate daughter cells. As in mitosis, in meiosis, diploid cells containing 2 homologous chromosomes undergo DNA replication to generate sister chromatids, but with 2 divisions. In the first meiotic division, homologous chromosomes are segregated, followed by sister chromatids in the second division. If a conversion event occurs in mitosis (marked as orange star, left part of diagram), then the conversion event will be inherited in 2 of 4 gametes in the following meiosis. Whereas, if a conversion event occurs in meiosis (marked as orange star, right part of diagram), then the conversion event will be inherited in only 1 of 4 gametes. Meiosis processes and mitosis processes are showing in dotted trapezoid and dotted square, respectively. (B) If we only focus on the vertical line from zygote to gamete (seed to seed) and there is no cell competition between recombinant cells and nonrecombinant cells, each division will generate 1/2Rmi of conversion rate (1/2, 50:50 inheritable possibility; Rmi, mitotic conversion rate per cell division). (PDF) [file pbio.3001164.s007.pdf]

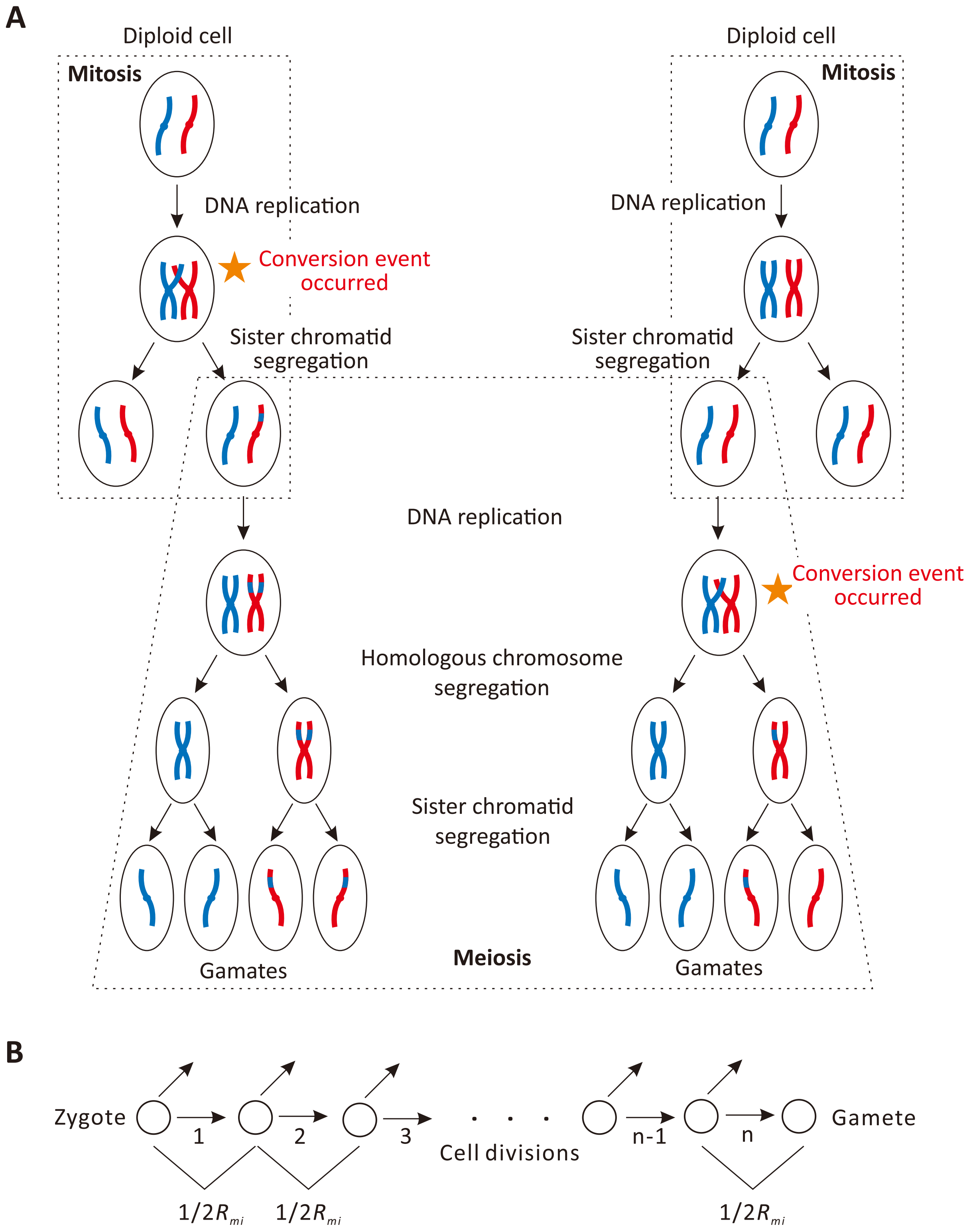

S7 Fig. Cell division and inherited pattern in gametes of mitotic and meiotic conversion.

(A) During mitosis, a diploid cell containing a pair of homologous chromosomes (one red and one blue) undergoes DNA replication to generate sister chromatids. In the mitotic division, sister chromatids are segregated to separate daughter cells. As in mitosis, in meiosis, diploid cells containing two homologous chromosomes undergo DNA replication to generate sister chromatids, but with two divisions. In the first meiotic division, homologous chromosomes are segregated, followed by sister chromatids in the second division. If a conversion event occurs in mitosis (marked as orange star, left part of diagram), then the conversion event will be inherited in two of four gametes

in the following meiosis. Whereas, if a conversion event occurs in meiosis (marked as orange star, right part of diagram), then the conversion event will be inherited in only one of four gametes. Meiosis processes and mitosis processes are showing in dotted trapezoid and dotted square, respectively.

(B) If we only focus on the vertical line from zygote to gamete (seed to seed) and there is no cell competition between recombinant cells and non-recombinant cells, each division will generate $1/2R_{mi}$ of conversion rate (1/2, 50:50 inheritable possibility; R_{mi}, mitotic conversion rate per cell division).
