# Supplementary material for: Mitotic gene conversion can be as important as meiotic conversion in driving genetic variability in plants and other species without early germline segregation
Source: PLoS Biol. 2021 Mar 22;19(3):e3001164. doi: 10.1371/journal.pbio.3001164 (PMC8016264; doi:10.1371/journal.pbio.3001164)
Supplement: S8 Fig — Red tracts present the homozygous genotypes of pseudo parent A, yellow tracts present the heterozygous genotypes of pseudo parent A and B. The PCR marker channel shows the 15 markers used in Fig 2, and the WGS marker channel presents the markers identified by WGS data. One of them (LLY2) had poorly covered reads in this region and so was validated by PCR results of new WGS markers (S4 and S5 Tables). LLY, Longliangyou1353; WGS, whole genome sequencing. (PDF) [file pbio.3001164.s008.pdf]

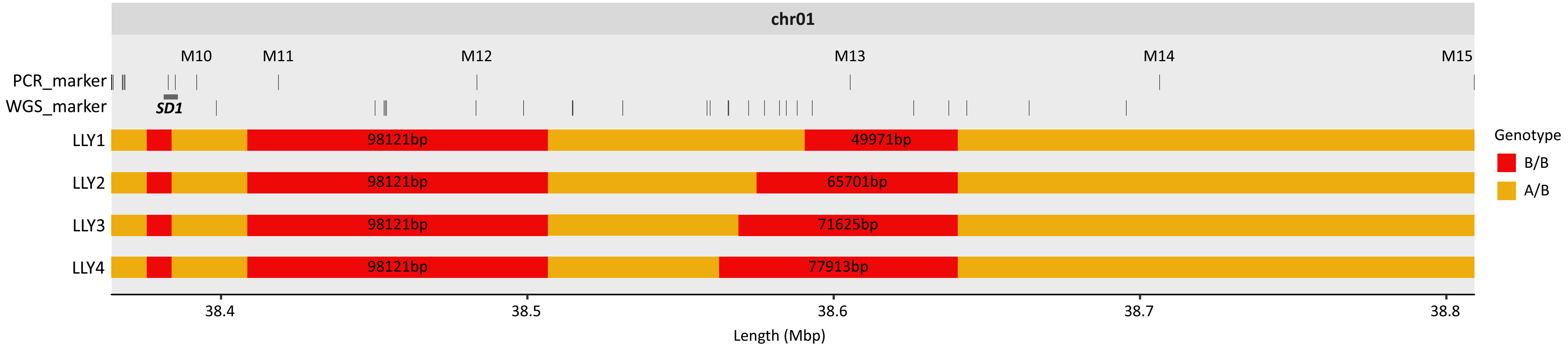

S8 Fig. Fine-scale dissection of recombination events in *SD1* region by WGS data of Type3 samples. Red tracts present the homozygous genotypes of pseudo parent A, yellow tracts present the heterozygous genotypes of pseudo parent A and B. The PCR marker channel shows the 15 markers used in Fig. 2 and the WGS marker channel presents the markers identified by WGS data. One of them (LLY2) had poorly-covered reads in this region and so was validated by PCR results of new WGS markers (S4 Table and S5 Table).
