# Supplementary material for: Mitotic gene conversion can be as important as meiotic conversion in driving genetic variability in plants and other species without early germline segregation
Source: PLoS Biol. 2021 Mar 22;19(3):e3001164. doi: 10.1371/journal.pbio.3001164 (PMC8016264; doi:10.1371/journal.pbio.3001164)
Supplement: S9 Fig — We consider a series of alternative models. First (method 1), we consider what happens if all meiotic tracts associated with 1 conversion are given the same tract length as meiotic events converting 1 marker. These can include Type 9, the event we previously excluded as it is called large but is in a region of low marker density. Second (method 2), we consider mean sum tract length per mitotic event rather than mean tract length. That is, if the sum tract length across all mitotic conversions is S, if there are N plants with identified mitotic conversion and M tracts (M>N as some plants had more than 1 conversion tract in the vicinity of SD1), then here we assume a mean conversion length (in 1 block or many) per event of S/N, rather than S/M considered previously. We adopted a similar simulation as for somatic conversion impact simulation for those 4 situations (details in Materials and methods). Methods 1 and 2 can be combined. A. Method 2, excluding Tract 9, low marker density estimate of meiotic tract size (equivalent to Fig 3B); B. Method 2, excluding Tract 9, high (WGS) marker density estimate of meiotic tract size (equivalent to Fig 3C); C. Method 1, including Tract 9, low marker density estimate of meiotic tract size; D. Method 1, including Tract 9, high marker density estimate of meiotic tract size; E. Method 1+2, including Tract 9, low marker density estimate of meiotic tract size; F. Method 1+2, including Tract 9, high marker density estimate of meiotic tract size. Underlying numerical values are presented in S2 Data. WGS, whole genome sequencing. (PDF) [file pbio.3001164.s009.pdf]

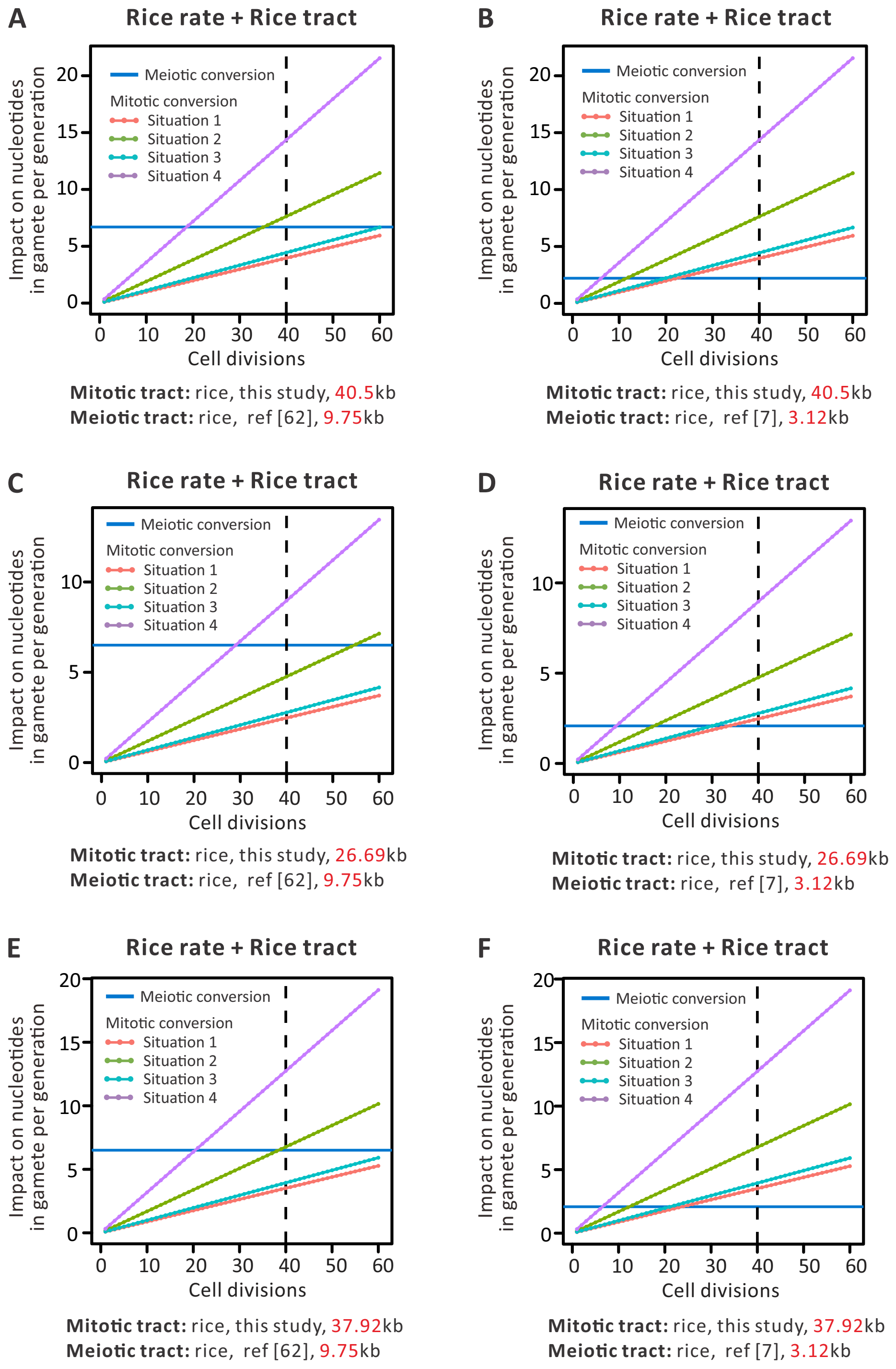

S9 Fig. Further consideration of relative transmission (parent to progeny) probability of meiotic and mitotic conversion events per generation, as a function of the number of cell generations from zygote to gamete. We consider a series of alternative models. First (method 1) we consider what happens if all meiotic tracts associated with one conversion are given the same tract length as meiotic events converting one marker. These can include Type 9, the event we previously excluded as it is called large but is in a region of low marker density. Second (method 2), we consider mean sum tract length per mitotic event rather than mean tract length. That is, if the sum tract length across all mitotic conversions is S, if there are N plants with identified mitotic conversion and M tracts (M>N as some plants had more than one conversion tract in the vicinity of SD1), then here we assume a mean conversion length (in one block or many) per event of S/N, rather than S/M considered previously. We adopted a similar simulation as for somatic conversion impact simulation for those four situations (details in Materials and Methods). Methods 1 and 2 can be combined. **A**. Method 2, excluding Tract 9, low marker density estimate of meiotic tract size (equivalent to Fig 3B); **B**. Method 2, excluding Tract 9, high (WGS) marker density estimate of meiotic tract size (equivalent to Fig 3C); **C**. Method 1, including Tract 9, low marker density estimate of meiotic tract size; **D**. Method 1, including Tract 9, high marker density estimate of meiotic tract size; **E**. Method 1+2, including Tract 9, low marker density estimate of meiotic tract size; **F**. Method 1+2, including Tract 9, high marker density estimate of meiotic tract size.
