# Supplementary material for: Mitotic gene conversion can be as important as meiotic conversion in driving genetic variability in plants and other species without early germline segregation
Source: PLoS Biol. 2021 Mar 22;19(3):e3001164. doi: 10.1371/journal.pbio.3001164 (PMC8016264; doi:10.1371/journal.pbio.3001164)
Supplement: S10 Fig — To compare the transmissive probability of mitotic and meiotic CO events, we took out a similar simulation as somatic conversion impact simulation for those 4 situations (details in Materials and methods). After simulation, we find that the expected inheritable probability of somatic CO events will be at least 10% of COs detected in F2 individuals per generation (assuming 40 divisions per generation in plants [35]). Underlying numerical values are presented in S2 Data. CO, crossover. (PDF) [file pbio.3001164.s010.pdf]

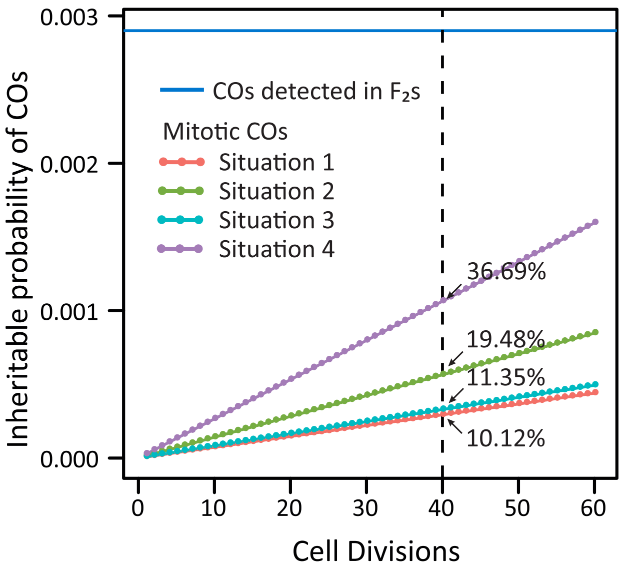

S10 Fig. Relative transmission (parent to progeny) probability of meiotic and mitotic CO events per generation, as a function of the number of cell generations from zygote to gamete.

To compare the transmissive probability of mitotic and meiotic CO events, we took out a similar simulation as somatic conversion impact simulation for those four situations (details in Materials and Methods). After simulation, we find that the expected inheritable probability of somatic CO events will be at least 10% of COs detected in F_2 individuals per generation (assuming 40 divisions per generation in plants (Hoffman et al., 2004)).
