# Supplementary material for: Mitotic gene conversion can be as important as meiotic conversion in driving genetic variability in plants and other species without early germline segregation
Source: PLoS Biol. 2021 Mar 22;19(3):e3001164. doi: 10.1371/journal.pbio.3001164 (PMC8016264; doi:10.1371/journal.pbio.3001164)
Supplement: S11 Fig — In meiosis, a single CO will generate 2 reciprocal rearrangement gametes (genotypes on right and left sides of the break point are from different parents), while double CO and long NCO will generate similar gametes (Gamete2 and 3 for double CO, Gamete2 for long NCO), in which genotypes of flanking sequences of the tract are from the same parents. CO, crossover; NCO, noncrossover. (PDF) [file pbio.3001164.s011.pdf]

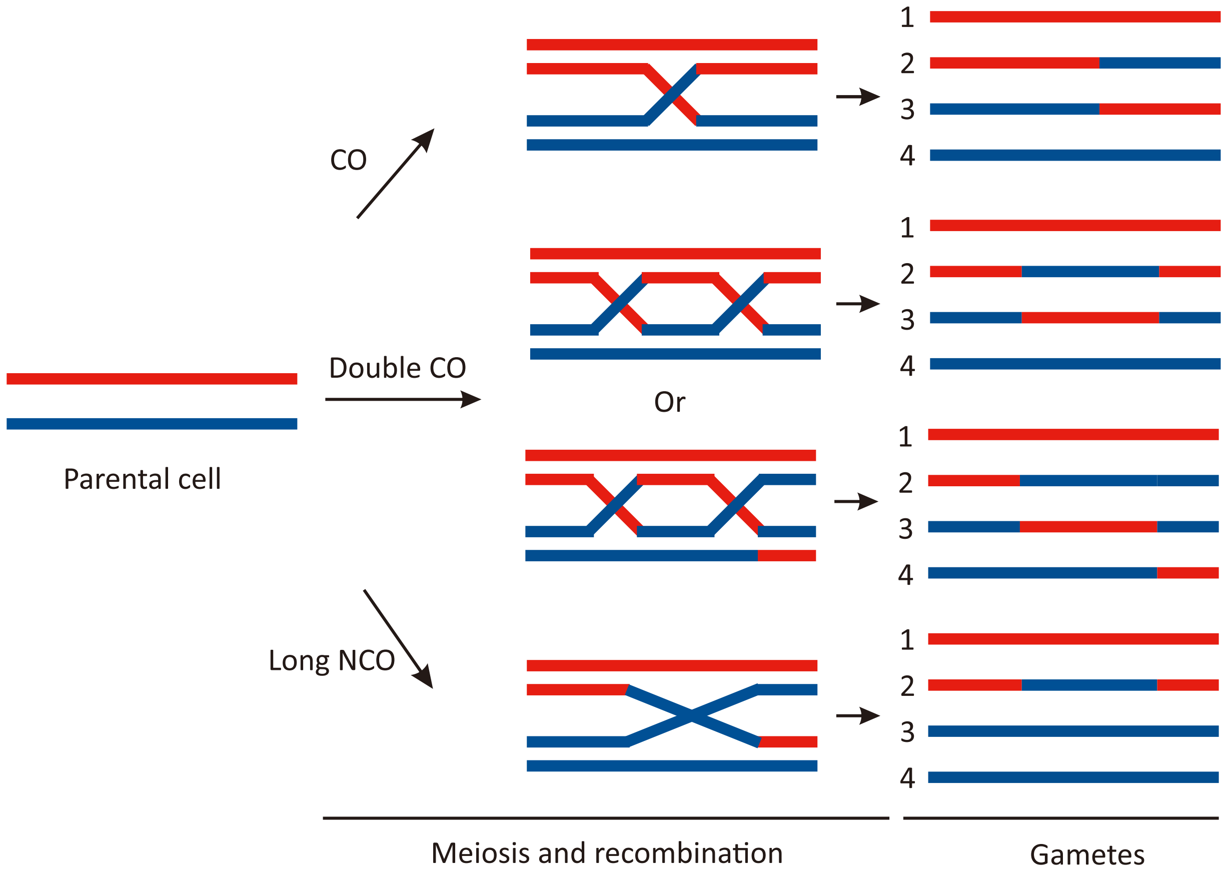

S11 Fig. Schematic of outcomes of double crossover and long non-crossover in meiosis.

In meiosis, a single crossover (CO) will generate two reciprocal rearrangement gametes (genotypes on right and left sides of the break point are from different parents), while double CO and long non-crossover (NCO) will generate similar gametes (Gamete3 for double CO, Gamete2 for long NCO), in which genotypes of flanking sequences of the tract are from the same parents.
