# Supplementary material for: Mitotic gene conversion can be as important as meiotic conversion in driving genetic variability in plants and other species without early germline segregation
Source: PLoS Biol. 2021 Mar 22;19(3):e3001164. doi: 10.1371/journal.pbio.3001164 (PMC8016264; doi:10.1371/journal.pbio.3001164)
Supplement: S1 Table — (DOCX) [file pbio.3001164.s012.docx]

**S1 Table. Summary of recombination rate and conversion tract length in prior studies in yeast, Arabidopsis and tobacco.**

| Species | Strategy | Recombination rate | Conversion tract length | References |
| --- | --- | --- | --- | --- |
| Yeast | Marker-based (URA3) | 1.7 – 8.1 × 10^-7^ events per division | - | Jinks-Robertson and Petes 1986 [1] |
| Yeast | Marker-based (LEU2) | ~10^-6^ to 10^-8^ events per division | 2.7 kb to 20 kb | Lichten and Haber 1989 [2] |
| Yeast | Marker-based (URA3) | 1.5 to 4.9 × 10^-6^ events per division | - | Steele et al. 1991 [3] |
| Yeast | Marker-based (URA3) | 2.4 to 9.9 × 10^-7^ events per division | - | Nickoloff et al. 1999 [4] |
| Yeast | Marker-based (URA3) | ~1.3 × 10^-6^ events per division | 1-21 kb and 36-121 kb | Yim et al. 2014 [5] |
| Yeast | Marker-based (FS2) | - | 3.9-16.8 kb and 29.2-78.8 kb | Chumki et al. 2016 [6] |
| Yeast | Marker-based (SUP4) | - | 3.6-17 kb | Yin et al. 2017 [7] |
| Tobacco | Resistance selection | ~3 × 10^-5^ to ~10^-6^ events per division | - | Tovar and Lichtenstein 1992 [8] |
| Arabidopsis | Resistance selection | ~10^-6^ events per division | - | Assaad and Signer 1992 [9] |
| Arabidopsis | GUS | ~10^-6^ to 10^-7^ events per plant | - | Swoboda et al. 1994 [10] |
| Arabidopsis | GUS | 0.2 to 2 sectors per plant ^*^ | - | Molinier et al. 2006 [11] |
| Arabidopsis | GUS | 0.5 ± 0.1 sectors per plant ^*^ | - | Nibau et al. 2020 [12] |

“-” means no data. ^*^In these studies, researchers counted the recombination sectors but can’t calculate recombination rates, owing to lack of knowledge of the total number of cells.

**References:**

1. Jinks-Robertson S, Petes TD. Chromosomal Translocations Generated by High-Frequency Meiotic Recombination between Repeated Yeast Genes. Genetics. 1986;114: 731–752.

2. Lichten M, Haber JE. Position effects in ectopic and allelic mitotic recombination in Saccharomyces cerevisiae. Genetics. 1989;123: 261–268.

3. Steele DF, Morris ME, Jinks-Robertson S. Allelic and Ectopic Interactions in Recombination-Defective Yeast Strains. Genetics. 1991;127: 53–60.

4. Nickoloff JA, Sweetser DB, Clikeman JA, Khalsa GJ, Wheeler SL. Multiple Heterologies Increase Mitotic Double-Strand Break-Induced Allelic Gene Conversion Tract Lengths in Yeast. Genetics. 1999;153: 665–679.

5. Yim E, O’Connell KE, Charles JS, Petes TD. High-Resolution Mapping of Two Types of Spontaneous Mitotic Gene Conversion Events in Saccharomyces cerevisiae. Genetics. 2014;198: 181–192. doi:10.1534/genetics.114.167395

6. Chumki SA, Dunn MK, Coates TF, Mishler JD, Younkin EM, Casper AM. Remarkably Long-Tract Gene Conversion Induced by Fragile Site Instability in Saccharomyces cerevisiae. Genetics. 2016;204: 115–128. doi:10.1534/genetics.116.191205

7. Yin Y, Dominska M, Yim E, Petes TD. High-resolution mapping of heteroduplex DNA formed during UV-induced and spontaneous mitotic recombination events in yeast. de Massy B, editor. eLife. 2017;6: e28069. doi:10.7554/eLife.28069

8. Tovar J, Lichtenstein C. Somatic and Meiotic Chromosomal Recombination between Inverted Duplications in Transgenic Tobacco Plants. Plant Cell. 1992;4: 319–332.

9. Assaad FF, Signer ER. Somatic and Germinal Recombination of a Direct Repeat in Arabidopsis. Genetics. 1992;132: 553–566.

10. Swoboda P, Gal S, Hohn B, Puchta H. Intrachromosomal homologous recombination in whole plants. EMBO J. 1994;13: 484–489.

11. Molinier J, Ries G, Zipfel C, Hohn B. Transgeneration memory of stress in plants. Nature. 2006;442: 1046–1049. doi:10.1038/nature05022

12. Nibau C, Lloyd A, Dadarou D, Betekhtin A, Tsilimigka F, Phillips DW, et al. CDKG1 Is Required for Meiotic and Somatic Recombination Intermediate Processing in Arabidopsis. Plant Cell. 2020;32: 1308–1322. doi:10.1105/tpc.19.00942
