# Supplementary material for: Mitotic gene conversion can be as important as meiotic conversion in driving genetic variability in plants and other species without early germline segregation
Source: PLoS Biol. 2021 Mar 22;19(3):e3001164. doi: 10.1371/journal.pbio.3001164 (PMC8016264; doi:10.1371/journal.pbio.3001164)
Supplement: S2 Table — (DOCX) [file pbio.3001164.s013.docx]

**S2 Table. Genotypes of M_1_ to M_15_ in 24 LYP9 (F_1_) individuals.**

| Sample | PH(cm) | M_1_^*^ | M_2_ | M_3_ | M_4_ | M_5_ | M_6_ | M_7_ | M_8_ | M_9_ | M_10_ | M_11_ | M_12_ | M_13_ | M_14_ | M_15_ | Type |
| --- | --- | --- | --- | --- | --- | --- | --- | --- | --- | --- | --- | --- | --- | --- | --- | --- | --- |
| H12 | 146 | H | H | H | H | H | H | H | H | P | H | H | H | H | H | H | Type1 (NCO-GC) |
| H15 | 161 | H | H | H | H | H | H | H | H | P | H | H | H | H | H | H | Type1 (NCO-GC) |
| H22 | 165 | H | H | H | H | H | H | H | H | P | H | H | H | H | H | H | Type1 (NCO-GC) |
| H1 | 132 | H | H | H | H | H | H | H | H | P | P | P | P | P | P | P | Type4 (CO) |
| H2 | 135 | H | H | H | H | H | H | H | H | P | P | P | P | P | P | P | Type4 (CO) |
| H3 | 139 | H | H | H | H | H | H | H | H | P | P | P | P | P | P | P | Type4 (CO) |
| H4 | 156 | H | H | H | H | H | H | H | H | P | P | P | P | P | P | P | Type4 (CO) |
| H6 | 161 | H | H | H | H | H | H | H | H | P | P | P | P | P | P | P | Type4 (CO) |
| H7 | 163 | H | H | H | H | H | H | H | H | P | P | P | P | P | P | P | Type4 (CO) |
| H8 | 151 | H | H | H | H | H | H | H | H | P | P | P | P | P | P | P | Type4 (CO) |
| H9 | 157 | H | H | H | H | H | H | H | H | P | P | P | P | P | P | P | Type4 (CO) |
| H10 | 136 | H | H | H | H | H | H | H | H | P | P | P | P | P | P | P | Type4 (CO) |
| H11 | 136 | H | H | H | H | H | H | H | H | P | P | P | P | P | P | P | Type4 (CO) |
| H13 | 133 | H | H | H | H | H | H | H | H | P | P | P | P | P | P | P | Type4 (CO) |
| H14 | 155 | H | H | H | H | H | H | H | H | P | P | P | P | P | P | P | Type4 (CO) |
| H16 | 154 | H | H | H | H | H | H | H | H | P | P | P | P | P | P | P | Type4 (CO) |
| H19 | 155 | H | H | H | H | H | H | H | H | P | P | P | P | P | P | P | Type4 (CO) |
| H20 | 144 | H | H | H | H | H | H | H | H | P | P | P | P | P | P | P | Type4 (CO) |
| H21 | 151 | H | H | H | H | H | H | H | H | P | P | P | P | P | P | P | Type4 (CO) |
| H23 | 149 | H | H | H | H | H | H | H | H | P | P | P | P | P | P | P | Type4 (CO) |
| H24 | 160 | H | H | H | H | H | H | H | H | P | P | P | P | P | P | P | Type4 (CO) |
| H17 | 144 | P | P | P | P | P | P | P | H | P | P | P | P | P | P | P | Type8 (CO-GC) |
| H18 | 138 | P | P | P | P | P | P | P | H | P | P | P | P | P | P | P | Type8 (CO-GC) |
| H5 | 156 | H | H | H | H | H | H | H | H | P | P | P | H | P | P | P | Type8 (CO-GC) |
| C1 | 118 | H | H | H | H | H | H | H | H | H | H | H | H | H | H | H | Non-recombinant |
| C2 | 106 | H | H | H | H | H | H | H | H | H | H | H | H | H | H | H | Non-recombinant |
| C3 | 112 | H | H | H | H | H | H | H | H | H | H | H | H | H | H | H | Non-recombinant |

Lines H1 to H24 are 24 tall LYP9 F_1_ individuals, which were screened from ~1,100,000 LYP9 individuals, and C1 to C3 are three randomly selected F_1_s with semi-dwarf statures. We detected a total of 3 NCO-GCs, 18 CO s, and 3 CO-GCs in 24 tall LYP9 F_1_ individuals. DNA was extracted from the flag leaf of each individual, and amplification was carried out by primers listed in S5, then genotypes were identified by Sanger sequencing. P, N and H stand for genotypes of homozygous PA64s, homozygous 93-11 and heterozygous PA64s/93-11, respectively. Two markers (M_8_ and M_9_) on the *SD1* gene are marked by grey background. PH, plant height. CO, crossover. NCO-GC, non-crossover gene conversion. CO-GC, crossover associated gene conversion. ^*^ M_1_ to M_15_ indicate the 15 markers described in Fig. 2.
