# Supplementary material for: Mitotic gene conversion can be as important as meiotic conversion in driving genetic variability in plants and other species without early germline segregation
Source: PLoS Biol. 2021 Mar 22;19(3):e3001164. doi: 10.1371/journal.pbio.3001164 (PMC8016264; doi:10.1371/journal.pbio.3001164)
Supplement: S3 Table — The whole-genome sequencing data of 2 parents (93–11 and PA64s) and normal LYP9 (F1) are from Si and colleagues in 2015 [7]. Eight LYP9 individuals were randomly selected from 24 tall F1 individuals. Using the same marker (polymorphism loci) set as previous research (a total of 871,863 markers) [7], 93.68% to 98.42% heterozygous markers from 93–11 and PA64s were detected in 8 tall F1 individuals. Meanwhile, 90.67% to 92.85% heterozygous markers were detected in 4 recombinants from LLY cross. All these data indicate that these tall recombinants are real F1 generation and independent to each other. Recall rate of markers column shows the ratio of heterozygous markers from parental lines detected in F1 individuals. The coverage depth is calculated as the number of bases of all reads that match a genome divided by the length of the rice genome. Genome coverage shows the proportion of covered region in the whole genome. Read numbers (M) show the total numbers of each library, and mapping rate column shows percent of mapped reads out of total reads. (DOCX) [file pbio.3001164.s014.docx]

**S3 Table.** **Summary statistics for whole-genome sequencing data of 12 recombinant individuals.** The whole-genome sequencing data of two parents (93-11 and PA64s) and normal LYP9 (F_1_) are from Si *et al.* in 2015 (Si et al. 2015). Eight LYP9 individuals were randomly selected from 24 tall F_1_ individuals. Using the same marker (polymorphism loci) set as in Si et al’s research (a total of 871,863 markers) (Si et al. 2015), 93.68% to 98.42% heterozygous markers from 9311 and PA64s were detected in eight tall F_1_ individuals. Meanwhile, 90.67% to 92.85% heterozygous markers were detected in four recombinants from LLY cross. All these data indicate that these tall recombinants are real F_1_ generation and independent to each other. Recall rate of markers column shows the ratio of heterozygous markers from parental lines detected in F_1_ individuals. The coverage depth is calculated as the number of bases of all reads that match a genome divided by the length of the rice genome; Genome coverage shows the proportion of covered region in the whole genome. Read numbers (M) show the total numbers of each library, and mapping rate column shows percent of mapped reads out of total reads.

| Cross | Samples | Recall rate of markers | Depth of coverage | Genome coverage | Read numbers (M) | Mapping rate |
| --- | --- | --- | --- | --- | --- | --- |
| LYP9 | LYP9_F1^*^ | 100.00% | 71.34 | 91.50% | 275.70 | 97.92% |
|  | H1 | 97.82% | 26.01 | 95.21% | 70.08 | 94.17% |
|  | H3 | 98.89% | 30.66 | 94.72% | 80.42 | 96.75% |
|  | H8 | 93.68% | 30.69 | 95.16% | 81.30 | 95.85% |
|  | H10 | 94.04% | 26.32 | 94.78% | 71.24 | 93.84% |
|  | H12 | 94.45% | 26.14 | 94.74% | 71.23 | 93.31% |
|  | H14 | 94.65% | 30.22 | 95.83% | 80.54 | 95.37% |
|  | H17 | 96.19% | 26.49 | 94.78% | 68.54 | 97.78% |
|  | H18 | 98.42% | 26.30 | 94.77% | 68.51 | 97.31% |
| LLY | LLY-C^**^ | 100.00% | 21.75 | 93.06% | 56.09 | 98.60% |
|  | LLY1 | 92.85% | 24.82 | 93.86% | 63.99 | 98.42% |
|  | LLY2 | 90.67% | 20.86 | 93.69% | 56.75 | 98.33% |
|  | LLY3 | 91.95% | 24.38 | 93.84% | 62.86 | 98.43% |
|  | LLY4 | 92.06% | 23.89 | 93.96% | 61.64 | 98.37% |

^*^ data from Si *et al.*, 2015. ^**^control sample (with semi-dwarf plant height, neighbor to these four tall individuals) for LLY lines.
