# Supplementary material for: Mitotic gene conversion can be as important as meiotic conversion in driving genetic variability in plants and other species without early germline segregation
Source: PLoS Biol. 2021 Mar 22;19(3):e3001164. doi: 10.1371/journal.pbio.3001164 (PMC8016264; doi:10.1371/journal.pbio.3001164)
Supplement: S4 Table — (DOCX) [file pbio.3001164.s015.docx]

**S4 Table. Markers used in identification of recombination events.**

| **PCR markers:** | | | | | |
| --- | --- | --- | --- | --- | --- |
| Marker | Chr | Position | Primer | Genotype | |
|  |  |  |  | PA64s (P) | 93-11 (N) |
| M_1_ | chr01 | 38364231 | primer1 | T | C |
| M_2_ | chr01 | 38364351 |  | Ins (8bp^*^) | T |
| M_3_ | chr01 | 38364774 |  | ---- | GT |
| M_4_ | chr01 | 38367784 | primer2 | A | C |
| M_5_ | chr01 | 38367866 |  | ---- | GATC |
| M_6_ | chr01 | 38368329 | primer3 | C | A |
| M_7_ | chr01 | 38368650 |  | T | A |
| M_8_ | chr01 | 38382761 | primer4 | Del (383bp^*^) | 383bp |
| M_9_ | chr01 | 38385064 | primer5 | C | G |
| M_10_ | chr01 | 38392050 | primer6 | TTAAACG | C------ |
| M_11_ | chr01 | 38418739 | primer7 | G | A |
| M_12_ | chr01 | 38483533 | primer8 | T | C |
| M_13_ | chr01 | 38605389 | primer9 | T | G |
| M_14_ | chr01 | 38706387 | primer10 | T | C |
| M_15_ | chr01 | 38809118 | primer11 | T | G |
|  | | | | | |
| **WGS markers for Type3:** | | | | | |
| Marker | Chr | Position | Primer | Genotype | |
|  |  |  |  | Pseudo-parent A (A) | Pseudo-parent B (B) |
| M_n1 | chr01 | 38398467 |  | A | G |
| M_n2 | chr01 | 38450279 |  | G | A |
| M_n3 | chr01 | 38453214 | primer12 | C | T |
| M_n4 | chr01 | 38453615 |  | C | T |
| M_n5 | chr01 | 38454000 |  | G | A |
| M_n6 | chr01 | 38483233 |  | G | A |
| M_n7 | chr01 | 38498773 | primer13 | G | C |
| M_n8 | chr01 | 38514678 | primer14 | C | A |
| M_n9 | chr01 | 38514877 |  | G | C |
| M_n10 | chr01 | 38531141 | primer15 | C | CATAT |
| M_n11 | chr01 | 38558649 |  | AAATTCGGTCATC | A |
| M_n12 | chr01 | 38559668 | primer16 | A | AG |
| M_n13 | chr01 | 38565528 | primer17 | AT | A |
| M_n14 | chr01 | 38565549 |  | T | C |
| M_n15 | chr01 | 38572224 | primer18 | A | G |
| M_n16 | chr01 | 38577397 |  | T | TC |
| M_n17 | chr01 | 38582309 | primer19 | C | G |
| M_n18 | chr01 | 38584523 |  | G | A |
| M_n19 | chr01 | 38588079 | primer20 | A | T |
| M_n20 | chr01 | 38593002 |  | C | T |
| M_n21 | chr01 | 38626113 | primer21 | C | T |
| M_n22 | chr01 | 38637581 | primer22 | G | A |
| M_n23 | chr01 | 38643444 | primer23 | C | T |
| M_n24 | chr01 | 38663801 |  | T | TGCAGACAACC |
| M_n25 | chr01 | 38695517 |  | G | A |

^*^ The sequence was not shown in the table. Two markers (M_8_ and M_9_) on the *SD1* gene are marked by grey background.
