# Supplementary material for: Mitotic gene conversion can be as important as meiotic conversion in driving genetic variability in plants and other species without early germline segregation
Source: PLoS Biol. 2021 Mar 22;19(3):e3001164. doi: 10.1371/journal.pbio.3001164 (PMC8016264; doi:10.1371/journal.pbio.3001164)
Supplement: S5 Table — (DOCX) [file pbio.3001164.s016.docx]

**S5 Table. Primers used in *SD1* region for genotype and recombination identification.**

| Primer | | Sequence | Amplicon size (bp) |
| --- | --- | --- | --- |
| primer1 | forward | CCCAGACTCCAGTACACCAC | 785 |
|  | reverse | TTTAGAACCACATGAGACACCA |  |
| primer2 | forward | TACTACGCCTCGATTTATCCT | 594 |
|  | reverse | GATGTCTGTATTCTCACTTTGG |  |
| primer3 | forward | GCCTCCAAAGTGAGAATACAG | 817 |
|  | reverse | GCAAATTGACTTTCCCAGAG |  |
| primer4 | forward | GAACGGGAGGTATAAGAGCTG | 1066 |
|  | reverse | GACAAAGCGGATTCATCGAG |  |
| primer5 | forward | GAACGGGAGGTATAAGAGCTG | 314 |
|  | reverse | TTCTGTTCGTTCCGTTTCGT |  |
| primer6 | forward | GAACGGGAGGTATAAGAGCTG | 674 |
|  | reverse | CATTGCTGCCATATACGAGG |  |
| primer7 | forward | CTGAGTGCTGAGTATTGGAG | 1140 |
|  | reverse | GGGATGAATTTGGAGTTGCTG |  |
| primer8 | forward | CGAAGATGCAACAGAGGAGG | 255 |
|  | reverse | TCACCAATCTTCAACCCTTCCC |  |
| primer9 | forward | GCAACGGTTAGACGAGATTA | 1061 |
|  | reverse | ATCAATCCGCTTTCAACAT |  |
| primer10 | forward | AAACCTCCCATTGCAGTG | 610 |
|  | reverse | CCCAATGGTGCTCACTTC |  |
| primer11 | forward | CATCCAGAGGCTTACATATCA | 762 |
|  | reverse | GAACCTTTGGCTTGTATTTG |  |
| primerP | forward | CAGGTGTCAGCGAGGAGAT | 715 |
|  | reverse | GTCGGGGGAGATCCATATTA |  |
| primerN | forward | CAGGAGGGTGTACCAGAAGT | 2127 |
|  | reverse | AAAATCGGCTTCTGTTCGT |  |
| primer12 | forward | CGAGATTCAGAAACAGGACAG | 894 |
|  | reverse | CGCAGAAGGTGATATTTGTG |  |
| primer13 | forward | TAGCAATGGACTCCTTTCTCAG | 922 |
|  | reverse | GTAGACAGACACAAACAGAGGG |  |
| primer14 | forward | TTTGCCCTAGATTACCTGCC | 874 |
|  | reverse | AATGTTGTGCTGTTAGATTGGG |  |
| primer15 | forward | ATATAACCGAGAACTATGCGACCA | 758 |
|  | reverse | TCAACGGGAGAAATCATTCACAC |  |
| primer16 | forward | GACGCGATGGCAATGATGG | 835 |
|  | reverse | TGTAGAATTTGACAAAGCTCTGCC |  |
| primer17 | forward | AATATACACAAACCCACGCA | 1698 |
|  | reverse | ACTGTTGATTAACCTACCTGTG |  |
| primer18 | forward | GTGAGAGTGAGATGGAGTG | 654 |
|  | reverse | CAGATAGAGTAGGGTATAGGGT |  |
| primer19 | forward | TTTGGTACCACATATCCGAC | 1420 |
|  | reverse | AAATGAGGTTTGATGCCCA |  |
| primer20 | forward | CCAGAAGAAACCTTGCCGA | 2777 |
|  | reverse | TGTTAAGCTCCGAAGAACTC |  |
| primer21 | forward | TGAACGAGCTACATAACCTG | 997 |
|  | reverse | CCATTTCAACCTTCCACTTCC |  |
| primer22 | forward | CTCCGTACTCCGAAGATCTCAG | 939 |
|  | reverse | ACCGTCTCTTTACGCTAACC |  |
| primer23 | forward | GCTACGAGAGGTGGAAGAG | 885 |
|  | forward | TAGTCTACTGCTCCCTCTG |  |

To accurately reconstruct the haplotypes of each individual at *SD1* locus, four pairs of PCR primers (primer4, primer5, primerN and primerP) were employed to amplify the targeted regions for sequencing (S2C Fig). Among them, primer4 and primer5, were designed to cover the 383bp deletion (M_8_ site) and the C->G single base substitution (M_9_ site), respectively. The upper primer of primerP was designed around the break point of 383bp deletion, and the upper primer of primerN was among the 383bp deletion region. Therefore, these two primers could specifically amplify the PA64s haplotype (P) and the 93-11 haplotype (N) respectively. To further identify the type of each recombination event, we genotyped other 13 makers surrounding *SD1* locus using nine rest pairs of primers (S3D Fig).
