# Supplementary material for: Mitotic gene conversion can be as important as meiotic conversion in driving genetic variability in plants and other species without early germline segregation
Source: PLoS Biol. 2021 Mar 22;19(3):e3001164. doi: 10.1371/journal.pbio.3001164 (PMC8016264; doi:10.1371/journal.pbio.3001164)
Supplement: S6 Table — (DOCX) [file pbio.3001164.s017.docx]

**S6 Table. Proportions of recombinant cells and non-recombinant cells in different tissues of LYP9 (F_1_) individuals.**

| Samples | Tillers | Tissues | C-reads | G-reads | R-cells | NR-cells |
| --- | --- | --- | --- | --- | --- | --- |
| H1 | H1-t1 | Flag leaf | 34,635 | 0 | 100.00% | 0.00% |
|  |  | Basal leaf | 228,647 | 23 | 99.99% | 0.01% |
|  |  | Roots | 228,732 | 23,917 | 90.53% | 9.47% |
|  | H1-t2 | Flag leaf | 893,781 | 0 | 100.00% | 0.00% |
|  |  | Roots | 763,555 | 0 | 100.00% | 0.00% |
| H8 | H8-t1 | Flag leaf | 138,254 | 0 | 100.00% | 0.00% |
|  |  | Basal leaf | 267,729 | 44 | 99.98% | 0.02% |
|  |  | Roots | 23,741 | 2,443 | 90.67% | 9.33% |
|  | H8-t2 | Flag leaf | 867,310 | 0 | 100.00% | 0.00% |
|  |  | Roots | 798,381 | 0 | 100.00% | 0.00% |
|  | H8-t3 | Flag leaf | 767,883 | 0 | 100.00% | 0.00% |
|  |  | Roots | 813,445 | 0 | 100.00% | 0.00% |
|  | H8-t4 | Flag leaf | 387,049 | 0 | 100.00% | 0.00% |
|  |  | Roots | 335,894 | 0 | 100.00% | 0.00% |
| H10 | H10-t1 | Flag leaf | 459,594 | 0 | 100.00% | 0.00% |
|  |  | Roots | 472,767 | 32 | 99.99% | 0.01% |
| H12 | H12-t1 | Flag leaf | 343,501 | 0 | 100.00% | 0.00% |
|  |  | Basal leaf | 216,968 | 15 | 99.99% | 0.01% |
|  |  | Roots | 457,834 | 48 | 99.99% | 0.01% |
| H14 | H14-t1 | Flag leaf | 32,754 | 0 | 100.00% | 0.00% |
|  |  | Roots | 384,423 | 29,845 | 92.80% | 7.20% |
| H15 | H15-t1 | Flag leaf | 456,878 | 0 | 100.00% | 0.00% |
|  |  | Basal leaf | 351,615 | 110,425 | 76.10% | 23.90% |
|  |  | Roots | 188,268 | 29,076 | 86.62% | 13.38% |
| H17 | H17-t1 | Flag leaf | 306,219 | 0 | 100.00% | 0.00% |
|  |  | Roots | 341,191 | 0 | 100.00% | 0.00% |
| H18 | H18-t1 | Flag leaf | 671,653 | 0 | 100.00% | 0.00% |
|  |  | Roots | 476,579 | 38 | 99.99% | 0.01% |
| H22 | H22-t1 | Flag leaf | 203,283 | 0 | 100.00% | 0.00% |
|  |  | Basal leaf | 283,279 | 32 | 99.99% | 0.01% |
|  |  | Roots | 247,814 | 23 | 99.99% | 0.01% |
| C1 | C1-t1 | Flag leaf | 0 | 279,716 | 0.00% | 100.00% |
|  |  | Roots | 0 | 336,193 | 0.00% | 100.00% |
| C2 | C2-t1 | Flag leaf | 0 | 401,242 | 0.00% | 100.00% |
|  |  | Roots | 0 | 374,827 | 0.00% | 100.00% |
| C3 | C3-t1 | Flag leaf | 0 | 248,764 | 0.00% | 100.00% |
|  |  | Roots | 0 | 495,852 | 0.00% | 100.00% |

To evaluate the pattern of mosaicism of somatic cells, we measured flag leaves, basal leaves and roots in 13 different tillers derived from nine randomly selected individuals. These were employed to detect proportion of recombinant cells (R-cells) and non-recombinant cells (NR-cells). Based on gene panel sequencing after haplotype-specific nested PCR, because all recombinant cells harbor *N^wt^P^wt^/P^del^P^wt^* genotype, the ratio of N-cells to NR-cells provides the ratio of reads of PA64s genotype (marked as C-reads, genotype of PA64s at M_9_ is “C”) to 93-11 genotype (marked as G-reads, genotype of 93-11 at M_9_ is “G”) at the M_9_ site. Among them, all flag leaves presented 100% R-cells and a total of 8 roots of different individuals showed cell heterogeneity, specifically a range of 0.01% to 13.38% of NR-cells were detected in these root tissues. In addition, five basal leaves from different individuals were randomly selected and detected. And all of these basal leaves concurrently carried high ratios of R-cells and a range of 0.01% to 23.90% of NR-cells, showing that basal leaves harbor mosaic somatic cells. We also sampled different tillers from each individual, where the main shoot of each individual was named as “t1” and additional tillers as t2, etc. Data are also shown for representative normal plant height (semi dwarf) adjacent individuals (C1, C2 and C3) controls.
