# Supplementary material for: Mitotic gene conversion can be as important as meiotic conversion in driving genetic variability in plants and other species without early germline segregation
Source: PLoS Biol. 2021 Mar 22;19(3):e3001164. doi: 10.1371/journal.pbio.3001164 (PMC8016264; doi:10.1371/journal.pbio.3001164)
Supplement: S7 Table — (DOCX) [file pbio.3001164.s018.docx]

**S7 Table. Genotypes of M_1_ to M_15_ in 24 F_1_ individuals from the four additional crosses.**

| Sample | PH(cm) | M_1_^*^ | M_2_ | M_3_ | M_4_ | M_5_ | M_6_ | M_7_ | M_8_ | M_9_ | M_10_ | M_11_ | M_12_ | M_13_ | M_14_ | M_15_ | Type |
| --- | --- | --- | --- | --- | --- | --- | --- | --- | --- | --- | --- | --- | --- | --- | --- | --- | --- |
| CLYH4 | 159 | H | H | H | H | H | H | H | H | P | H | H | H | H | H | H | Type1 (NCO-GC) |
| HLY9 | 158 | H | H | H | H | H | H | H | H | P | H | H | H | H | H | H | Type1 (NCO-GC) |
| HLYH1 | 162 | H | H | H | H | H | H | H | H | P | H | H | H | H | H | H | Type1 (NCO-GC) |
| HLYH2 | 151 | H | H | H | H | H | H | H | H | P | H | H | H | H | H | H | Type1 (NCO-GC) |
| HLYH3 | 162 | H | H | H | H | H | H | H | H | P | H | H | H | H | H | H | Type1 (NCO-GC) |
| HLY4 | 169 | H | H | H | H | H | H | H | N | H | H | H | H | H | H | H | Type2 (NCO-GC) |
| LLY1 | 156 | H | H | H | H | H | H | H | N | H | H | N | N | N | H | H | Type3 (2 × NCO-GC) |
| LLY2 | 154 | H | H | H | H | H | H | H | N | H | H | N | N | N | H | H | Type3 (2 × NCO-GC) |
| LLY3 | 158 | H | H | H | H | H | H | H | N | H | H | N | N | N | H | H | Type3 (2 × NCO-GC) |
| LLY4 | 157 | H | H | H | H | H | H | H | N | H | H | N | N | N | H | H | Type3 (2 × NCO-GC) |
| CLYH6 | 170 | H | H | H | H | H | H | H | H | P | P | P | P | P | P | P | Type4 (CO) |
| CLYH7 | 165 | H | H | H | H | H | H | H | H | P | P | P | P | P | P | P | Type4 (CO) |
| HLY6 | 156 | H | H | H | H | H | H | H | H | P | P | P | P | P | P | P | Type4 (CO) |
| CLYH1 | 161 | N | N | N | N | N | N | N | N | H | H | H | H | H | H | H | Type5 (CO) |
| CLYH2 | 164 | N | N | N | N | N | N | N | N | H | H | H | H | H | H | H | Type5 (CO) |
| CLYH3 | 163 | N | N | N | N | N | N | N | N | H | H | H | H | H | H | H | Type5 (CO) |
| HLY3 | 165 | N | N | N | N | N | N | N | N | H | H | H | H | H | H | H | Type5 (CO) |
| HLY5 | 165 | N | N | N | N | N | N | N | N | H | H | H | H | H | H | H | Type5 (CO) |
| HLY10 | 168 | N | N | N | N | N | N | N | N | H | H | H | H | H | H | H | Type5 (CO) |
| HLY7 | 159 | N | N | N | N | N | N | N | N | H | H | H | H | H | N | N | Type6 (2 × CO) |
| HLY1 | 152 | N | N | N | N | N | N | N | N | H | N | N | N | N | N | N | Type7 (CO-GC) |
| HLY2 | 153 | N | N | N | N | N | N | N | N | H | N | N | N | N | N | N | Type7 (CO-GC) |
| HLY8 | 165 | N | N | N | N | N | N | N | N | H | N | N | N | N | N | N | Type7 (CO-GC) |
| CLYH5 | 150 | P | P | P | P | P | P | P | H | P | P | P | P | P | P | P | Type8 (CO-GC) |
| CLYH-c^**^ | 111 | H | H | H | H | H | H | H | H | H | H | H | H | H | H | H | Non-recombinant |
| HLY-c^**^ | 98 | H | H | H | H | H | H | H | H | H | H | H | H | H | H | H | Non-recombinant |
| HLYH-c^**^ | 102 | H | H | H | H | H | H | H | H | H | H | H | H | H | H | H | Non-recombinant |
| LLY-c^**^ | 106 | H | H | H | H | H | H | H | H | H | H | H | H | H | H | H | Non-recombinant |

24 tall individuals were sampled from four additional semi-dwarf hybrid rice varieties (F_1_ progeny) grown in expansive natural farmland planted by farmers around Nanjing in 2017 (S2B Fig.), and their surrounding individuals with normal plant heights were also sampled as controls (named as “-c” here). We detected a total of 14 NCO-GC, 11 CO, and 4 CO-GC events in these 24 tall F_1_ individuals. DNA was extracted from flag leaf of each individual, and amplification was carried out by primers listed in S5, then genotypes were identified by Sanger sequencing. P, N and H stand for genotypes of homozygous PA64s, homozygous 93-11 and heterozygous PA64s/93-11, respectively. Two markers (M_8_ and M_9_) on the *SD1* gene are marked by grey background. PH, plant height. CO, crossover. NCO-GC, non-crossover gene conversion. CO-GC, crossover associated gene conversion. ^*^ M_1_ to M_15_ indicate the 15 markers described in fig. 2. ^**^ Only one of the controls of each cross was listed here, and other controls show the same pattern with listed samples.
