# Supplementary material for: Mitotic gene conversion can be as important as meiotic conversion in driving genetic variability in plants and other species without early germline segregation
Source: PLoS Biol. 2021 Mar 22;19(3):e3001164. doi: 10.1371/journal.pbio.3001164 (PMC8016264; doi:10.1371/journal.pbio.3001164)
Supplement: S8 Table — In summary of 2 datasets, at least 29 CO (in LYP9 lines, 18 CO; in other 4 crosses, 11 CO), 17 NCO-GC (in LYP9 lines, 3 NCO-GC; in other 4 crosses, 14 NCO-GC), and 7 CO-GC (in LYP9 lines, 3 CO-GC; in other 4 crosses, 4 CO-GC) events had occurred in 48 tall individuals. There are 2 adjacent NCO-GC events and 2 adjacent CO events that occurred in recombination Type3 and Type6, respectively. (DOCX) [file pbio.3001164.s019.docx]

**S8 Table. Summary of recombinant individuals of LYP9 and other hybrid varieties.** In summary of two datasets, at least 29 CO (in LYP9 lines, 18 CO; in other four crosses, 11 CO), 17 NCO-GC (in LYP9 lines, 3 NCO-GC; in other four crosses, 14 NCO-GC) and 7 CO-GC (in LYP9 lines, 3 CO-GC; in other four crosses, 4 CO-GC) events had occurred in 48 tall individuals. there are two adjacent NCO-GC events and two adjacent CO events that occurred in recombination Type3 and Type6, respectively.

| No. | Recombination type | Total number | Tall individuals | |
| --- | --- | --- | --- | --- |
|  |  |  | LYP9 lines | Other hybrid varieties |
| Type1 | NCO-GC | 8 | H12, H15, H22 | CLYH4, HLY9, HLYH1, HLYH2, HLYH3 |
| Type2 | NCO-GC | 1 | - | HLY4 |
| Type3 | 2 × NCO-GC | 4 | - | LLY1, LLY2, LLY3, LLY4 |
| Type4 | CO | 21 | H1, H2, H3, H4, H6, H7, H8, H9, H10, H11, H13, H14, H16, H19, H20, H21, H23, H24 | CLYH6, CLYH7, HLY6 |
| Type5 | CO | 6 | - | CLYH1, CLYH2, CLYH3, HLY3, HLY5, HLY10 |
| Type6 | 2 × CO | 1 | - | HLY7 |
| Type7 | CO-GC | 3 | - | HLY1, HLY2, HLY8 |
| Type8 | CO-GC | 3 | H17, H18 | CLYH5 |
| Type9 | CO-GC | 1 | H5 | - |
