# Supplementary material for: Mitotic gene conversion can be as important as meiotic conversion in driving genetic variability in plants and other species without early germline segregation
Source: PLoS Biol. 2021 Mar 22;19(3):e3001164. doi: 10.1371/journal.pbio.3001164 (PMC8016264; doi:10.1371/journal.pbio.3001164)
Supplement: S9 Table — To evaluate heritability of these recombinant cells, 43 F2 progeny of 3 randomly selected tall individuals (H1, H8, and H14) were genotyped at the SD1 locus. Part of the progeny was tall with at least 130 cm (H8-P9) to 170 cm (N8-P9), and other progeny which present the same SD1 genotypes as their parents (N8-P9) were around 90–110 cm. (DOCX) [file pbio.3001164.s020.docx]

**S9 Table. Inheritance of tall phenotype and wildtype *SD1* gene of F_2_ progeny of three randomly selected tall LYP9 individuals.** To evaluate heritability of these recombinant cells, 43 F_2_ progeny of three randomly selected tall individuals (H1, H8 and H14) were genotyped at the *SD1* locus. Part of the progeny was tall with at least 130cm (H_8_-P_9_) to 170cm (N_8_-P_9_), and other progeny which present the same *SD1* genotypes as their parents (N_8_-P_9_), were around 90-110 cm.

| Sample | Tiller | Type | PH (cm) | M_8_ | M_9_ |
| --- | --- | --- | --- | --- | --- |
| H1 | H1-t1 | Progeny1 | 117 | P | P |
|  |  | Progeny2 | 159 | N | P |
| H8 | H8-t1 | Progeny1 | 145 | H | P |
|  |  | Progeny2 | 151 | H | P |
|  |  | Progeny3 | 149 | H | P |
|  |  | Progeny4 | 110 | P | P |
|  |  | Progeny5 | 148 | H | P |
|  |  | Progeny6 | 108 | P | P |
|  | H8-t2 | Progeny1 | 152 | H | P |
|  |  | Progeny2 | 107 | P | P |
|  |  | Progeny3 | 140 | H | P |
|  |  | Progeny4 | 138 | H | P |
|  |  | Progeny5 | 155 | N | P |
|  |  | Progeny6 | 147 | N | P |
|  | H8-t3 | Progeny1 | 140 | H | P |
|  |  | Progeny2 | 155 | N | P |
|  |  | Progeny3 | 98 | P | P |
|  |  | Progeny4 | 149 | H | P |
|  |  | Progeny5 | 142 | H | P |
|  |  | Progeny6 | 140 | H | P |
|  |  | Progeny7 | 165 | N | P |
|  | H8-t4 | Progeny1 | 101 | P | P |
|  |  | Progeny2 | 151 | H | P |
|  |  | Progeny3 | 149 | H | P |
|  |  | Progeny4 | 154 | N | P |
|  |  | Progeny5 | 99 | P | P |
|  |  | Progeny6 | 146 | H | P |
|  |  | Progeny7 | 147 | H | P |
|  |  | Progeny8 | 110 | P | P |
|  |  | Progeny9 | 149 | H | P |
|  |  | Progeny10 | 152 | H | P |
|  |  | Progeny11 | 142 | H | P |
|  |  | Progeny12 | 146 | H | P |
| H14 | H14-t1 | Progeny1 | 147 | H | P |
|  |  | Progeny2 | 161 | N | P |
|  |  | Progeny3 | 104 | P | P |
|  |  | Progeny4 | 146 | H | P |
|  |  | Progeny5 | 100 | P | P |
|  | H14-t2 | Progeny1 | 103 | P | P |
|  |  | Progeny2 | 157 | N | P |
|  |  | Progeny3 | 152 | H | P |
|  |  | Progeny4 | 102 | P | P |
|  |  | Progeny5 | 150 | H | P |

M_8_ and M_9_ indicate the markers described in. P, N and H stand for genotypes of homozygous PA64s, homozygous 93-11 and heterozygous PA64s/93-11, respectively.
