# Supplementary material for: Mitotic gene conversion can be as important as meiotic conversion in driving genetic variability in plants and other species without early germline segregation
Source: PLoS Biol. 2021 Mar 22;19(3):e3001164. doi: 10.1371/journal.pbio.3001164 (PMC8016264; doi:10.1371/journal.pbio.3001164)
Supplement: S10 Table — All these 3 F1 individuals have the same genotype (H8-P9) at markers 8 and 9, i.e., PdelPwt / NwtPwt, thus their progeny are expected to harbor NwtPwt / NwtPwt, PdelPwt / NwtPwt, and PdelPwt / PdelPwt, 3 kinds of alleles. After χ2 test, we found that the allele frequency for progeny of each F1 individual followed the segregation law, also indicating that all mitotic recombination events are well transmitted into their progeny. (DOCX) [file pbio.3001164.s021.docx]

**S10 Table. Individual distribution of *SD1* alleles for progeny of H1, H8 and H14.** All these three F_1_ individuals have the same genotype (H_8_-P_9_) at markers 8 and 9, i.e. *P^del^P^wt^* / *N^wt^P^wt^*, thus their progeny are expected to harbor *N^wt^P^wt^* / *N^wt^P^wt^*, *P^del^P^wt^* / *N^wt^P^wt^* and *P^del^P^wt^* / *P^del^P^wt^* three kinds of alleles. After *χ^2^*test, we found that the allele frequency for progeny of each F_1_ individual is followed the segregation law, also indicating that all mitotic recombination events are well transmitted into their progeny.

| Individual frequency | H1 | | H8 | | H14 | |
| --- | --- | --- | --- | --- | --- | --- |
|  | Obs. | Exp. | Obs. | Exp. | Obs. | Exp. |
| *N^wt^P^wt^* / *N^wt^P^wt^* | 1 | 0.5 | 5 | 7.75 | 2 | 2.5 |
| *P^del^P^wt^* / *N^wt^P^wt^* | 0 | 1 | 19 | 15.5 | 4 | 5 |
| *P^del^P^wt^* / *P^del^P^wt^* | 1 | 0.5 | 7 | 7.75 | 4 | 2.5 |
| *P* value (*χ^2^*test, *df* = 2) | 0.3679 (*χ^2^* = 2) | | 0.3988 (*χ^2^* = 1.8387) | | 0.5488 (*χ^2^* = 1.2) | |

Obs. and Exp. mean observed value and expected value, respectively.
