# Supplementary material for: Mitotic gene conversion can be as important as meiotic conversion in driving genetic variability in plants and other species without early germline segregation
Source: PLoS Biol. 2021 Mar 22;19(3):e3001164. doi: 10.1371/journal.pbio.3001164 (PMC8016264; doi:10.1371/journal.pbio.3001164)
Supplement: S11 Table — A total of 105 close double CO events (distance between 10 kb and 20 kb) were identified in 29 yeast tetrads. Among them, distances of 24 close double CO events are shorter than 12 kb. Break points of CO events are judged by midpoint method. Prebreak line and postbreak line are break points of front CO and break points of subsequent CO. Data from Liu and colleagues [8]. (DOCX) [file pbio.3001164.s022.docx]

**S11 Table. List of close double CO events (distance between 10 kb and 20kb) identified in yeast tetrads (data from Liu et al. 2018).** A total of 105 close double CO events (distance between 10 kb and 20kb) were identified in 29 yeast tetrads. Among them, distances of 24 close double CO events are shorter than 12kb. Break points of CO events are judged by midpoint method. Pre-break line and Post-break line are break points of front CO and break points of subsequent CO. Data from Liu et al. 2018 (Liu et al. 2018).

| Tetrad | Chromosome | Pre-break | Post-break | Distance (bp) |
| --- | --- | --- | --- | --- |
| S1-1 | IV | 1494817 | 1512205 | 17387.5 |
| S1-1 | XV | 270727.5 | 289921.5 | 19194 |
| S1-2 | III | 41711.5 | 56982 | 15270.5 |
| S1-2 | XIV | 391775.5 | 402118.5 | 10343 |
| S1-2 | XVI | 686316.5 | 704755.5 | 18439 |
| S1-4 | IX | 383105 | 399229.5 | 16124.5 |
| S1-5 | II | 535419 | 547929.5 | 12510.5 |
| S1-5 | IV | 506611.5 | 523292.5 | 16681 |
| S1-5 | XII | 527499.5 | 540592 | 13092.5 |
| S1-5 | XIV | 80171.5 | 91957.5 | 11786 |
| S1-6 | IX | 404809.5 | 423499 | 18689.5 |
| S1-6 | VIII | 125159 | 144913 | 19754 |
| S1-6 | X | 43109.5 | 60091.5 | 16982 |
| S1-6 | XVI | 694845 | 713647.5 | 18802.5 |
| S1-7 | I | 171641 | 191234.5 | 19593.5 |
| S1-7 | IV | 1334576 | 1344889 | 10313.5 |
| S1-7 | XI | 372900.5 | 389366.5 | 16466 |
| S1-7 | XII | 707918.5 | 727645 | 19726.5 |
| S1-8 | VIII | 48656 | 66114.5 | 17458.5 |
| S1-8 | XIII | 228778.5 | 242558 | 13779.5 |
| S1-9 | IV | 810760 | 827710.5 | 16950.5 |
| S1-9 | X | 78961 | 90621.5 | 11660.5 |
| S1-9 | X | 372365.5 | 391336.5 | 18971 |
| S1-9 | XI | 498974 | 514242 | 15268 |
| S1-9 | XII | 328754 | 341238.5 | 12484.5 |
| S1-9 | XII | 518273.5 | 536831 | 18557.5 |
| S1-10 | I | 120882.5 | 132385.5 | 11503 |
| S1-10 | VII | 120663.5 | 140169.5 | 19506 |
| S1-10 | X | 568826 | 584777.5 | 15951.5 |
| S1-10 | XIII | 701752 | 715395.5 | 13643.5 |
| S1-10 | XVI | 85273 | 99025 | 13752 |
| S1-11 | V | 173940.5 | 191099 | 17158.5 |
| S1-12 | I | 64378.5 | 80275 | 15896.5 |
| S1-12 | II | 645579.5 | 663684.5 | 18105 |
| S1-12 | XV | 74996 | 94313.5 | 19317.5 |
| S1-12 | XVI | 911139 | 922028 | 10889 |
| S1-13 | I | 41339.5 | 54581 | 13241.5 |
| S1-13 | I | 175520.5 | 191234.5 | 15714 |
| S1-13 | X | 320481 | 332440 | 11959 |
| S1-14 | II | 289028 | 305357.5 | 16329.5 |
| S1-14 | XII | 601482.5 | 616759.5 | 15277 |
| S1-15 | I | 114794 | 132385.5 | 17591.5 |
| S1-15 | VI | 215349.5 | 225499.5 | 10150 |
| S1-15 | XVI | 340017.5 | 357456.5 | 17439 |
| S2-1 | I | 15329.5 | 26528.5 | 11199 |
| S2-1 | III | 211016.5 | 224930 | 13913.5 |
| S2-1 | IV | 1220059 | 1239998 | 19939 |
| S2-1 | VI | 213361 | 229887.5 | 16526.5 |
| S2-1 | VII | 598059 | 615874 | 17815 |
| S2-1 | X | 380356 | 391116.5 | 10760.5 |
| S2-2 | I | 15329.5 | 26394.5 | 11065 |
| S2-2 | I | 26394.5 | 45371.5 | 18977 |
| S2-2 | III | 46358.5 | 59433.5 | 13075 |
| S2-2 | XI | 269188 | 283069 | 13881 |
| S2-2 | XII | 515578 | 534410.5 | 18832.5 |
| S2-2 | XII | 677067.5 | 692836 | 15768.5 |
| S2-2 | XIV | 571845.5 | 591784 | 19938.5 |
| S2-2 | XV | 482760.5 | 495086.5 | 12326 |
| S2-3 | I | 15329.5 | 26528.5 | 11199 |
| S2-3 | II | 356746 | 376334 | 19588 |
| S2-3 | V | 68019.5 | 80523 | 12503.5 |
| S2-3 | XV | 770835.5 | 787009 | 16173.5 |
| S2-4 | II | 128294.5 | 147138.5 | 18844 |
| S2-4 | XIII | 245682 | 257980.5 | 12298.5 |
| S2-4 | XIII | 838480.5 | 854670.5 | 16190 |
| S2-5 | I | 15262.5 | 26528.5 | 11266 |
| S2-5 | XIV | 532608.5 | 548192.5 | 15584 |
| S2-6 | VI | 57891.5 | 73490.5 | 15599 |
| S2-6 | XIII | 845378.5 | 860946 | 15567.5 |
| S2-6 | XIV | 47760.5 | 64888 | 17127.5 |
| S2-7 | I | 15322.5 | 27002.5 | 11680 |
| S2-7 | XV | 405372.5 | 422655.5 | 17283 |
| S2-8 | IV | 1477796 | 1495818 | 18022.5 |
| S2-8 | VIII | 143803.5 | 160454.5 | 16651 |
| S2-8 | VIII | 498538.5 | 512425 | 13886.5 |
| S2-8 | XI | 36737.5 | 54166.5 | 17429 |
| S2-8 | XVI | 85127.5 | 103669.5 | 18542 |
| S2-9 | I | 15329.5 | 27002.5 | 11673 |
| S2-9 | X | 235831.5 | 254053.5 | 18222 |
| S2-9 | XIII | 216188 | 230603.5 | 14415.5 |
| S2-9 | XIV | 521576.5 | 541495.5 | 19919 |
| S2-9 | XIV | 571845.5 | 586861 | 15015.5 |
| S2-9 | XIV | 586861 | 600987.5 | 14126.5 |
| S2-10 | IV | 129581 | 147520.5 | 17939.5 |
| S2-10 | XII | 48722.5 | 65657.5 | 16935 |
| S2-10 | XIV | 571845.5 | 587624 | 15778.5 |
| S2-10 | XIV | 587624 | 600987.5 | 13363.5 |
| S2-11 | I | 15262.5 | 26562.5 | 11300 |
| S2-11 | VII | 468878.5 | 486724 | 17845.5 |
| S2-11 | XIII | 827164.5 | 840012.5 | 12848 |
| S2-12 | I | 15329.5 | 26528.5 | 11199 |
| S2-12 | IV | 1459199 | 1479034 | 19834.5 |
| S2-12 | V | 474253.5 | 494172 | 19918.5 |
| S2-12 | VII | 1011966 | 1022150 | 10184.5 |
| S2-12 | XV | 832289.5 | 849915 | 17625.5 |
| S2-13 | I | 15964.5 | 26528.5 | 10564 |
| S2-13 | V | 46709 | 58667.5 | 11958.5 |
| S2-13 | XIII | 589998.5 | 601486.5 | 11488 |
| S2-14 | I | 15964.5 | 26528.5 | 10564 |
| S2-14 | IX | 69167.5 | 85386.5 | 16219 |
| S2-14 | XIV | 493066 | 503328 | 10262 |
| S2-15 | II | 652129.5 | 670379.5 | 18250 |
| S2-15 | V | 360211.5 | 374687 | 14475.5 |
| S2-15 | X | 672451.5 | 683182.5 | 10731 |
| S2-15 | XII | 955323.5 | 972733 | 17409.5 |
