# Supplementary material for: Mitotic gene conversion can be as important as meiotic conversion in driving genetic variability in plants and other species without early germline segregation
Source: PLoS Biol. 2021 Mar 22;19(3):e3001164. doi: 10.1371/journal.pbio.3001164 (PMC8016264; doi:10.1371/journal.pbio.3001164)
Supplement: S1 Text — (A). The canonical pattern of mitotic recombination. (B). Identification of recombination events. (C). Simulation of conversion and crossover rate per mitosis in different situations. (DOCX) [file pbio.3001164.s023.docx]

**S1 Text**

**A. The canonical pattern of mitotic recombination**

In general, mitosis starts with replication of the parental chromosomes, and the two homologues do not normally associate with one another [1–4]. Subsequently, the replicated chromosomes align on the mitotic division spindle independently, and the rule is that one daughter chromatid of each replicated chromosome segregates into each daughter nucleus, so that the two daughter diploid nuclei have the same genotypes as their parent cell (S1A Fig.). In rare cases, however, the two homolog chromosomes do associate with one another sufficiently closely for a crossover (CO) or non-crossover associated conversion (NCO-GC) to occur (S1B Fig.) [5]. This results in two of the homolog chromatids being recombinant. However, the recombinant replicated chromosomes still align independently on the mitotic division spindle, and still follow the rule that one sister chromatid of each replicated chromosome segregates into each daughter cell. For CO lines, there are two possible orientations on the mitotic division spindle [6]. One orientation produces two heterozygous diploid daughter nuclei, but one contains the two parental chromatids, and is exactly the same as the parental nucleus, whereas the other receives the two recombinant chromatids. The alternative orientation produces two diploid daughter nuclei that are homozygous from the point of the crossover to the end of the chromosome arm. In NCO-GC lines, there are two different genotypes based on the alterable conversion tract position in recombinant daughter cells [6,7]. In summary, outcomes of CO events present heterozygous genotypes on one side and homozygous genotypes on another side separated by a breakpoint, whereas outcomes of NCO-GC events show heterozygous genotypes on both sides of the tract but homozygous genotypes within the tract.

**B. Identification of recombination events**

After large-scale screening of F_1_ progeny a cross between 93-11 and PA64s, tall individuals with plant height > 130cm were collected, together with parental lines (height: 90-110cm) and normal F_1_s (height: 90-110cm) as controls (S3A Fig.). To validate that the changes of plant height derive from recombination events of *SD1* gene, two markers (M_8_ and M_9_) were genotyped and assigned to genotype P (PA64s homozygosity), N (93-11 homozygosity) or H (heterozygosity of PA64s and 93-11) (S3B Fig.). Primer4 and primer5 are used to genotype M_8_ and M_9_, respectively. As we described in the introduction, two semi-dwarf varieties, PA64s and 93-11, harbor two different defective alleles, here respectively denoted as P_8_-P_9_ and N_8_-N_9_. If a recombination event occurred between these two alleles, progeny with wild-type *SD1* gene will be generated and present tall statures, namely harboring three switched genotypes of M_8_ and M_9_ (N_8_-P_9_, N_8_-H_9_, H_8_-P_9_).

While the genotype of H_8_-H_9_ could be phased as P_8_-P_9_/N_8_-N_9_ or P_8_-N_9_/N_8_-P_9_, as we know that a normal F_1_ harbors the P_8_-P_9_/N_8_-N_9_ haplotypes, the P_8_-N_9_/N_8_-P_9_ for must come from a crossover event. Offspring with these two different haplotypes are respectively named as NF_1_ (non-recombinant F_1_) and RF_1_ (recombinant F_1_) here (S3C Fig.). To distinguish these two types, we employed two haplotype-specific primers primerP and primerN, which could specifically amplify the PA64s haplotype (P) and 93-11 haplotype (N), respectively (S3C Fig.). In detail, the forward primer of primerP pair covered the break site of 383bp deletion in the PA64 haplotype, whereas the forward primer of primerN pair is located in the 383 bp deletion and the amplified sequence of primerN covers M_9_ site. Expected PCR amplification results are shown in S3C Fig., specifically, individuals with both 93-11 and PA64s haplotypes could present combined electrophoretic bands of parental lines. After sequencing the amplification products of primer5 and primerN, NF_1_ and RF_1_ individuals showed as heterozygous at the M_9_ site (C/G nucleotide base, PA64/93-11 type) from amplification products of primer5, but respectively presented as homozygous G nucleotide base (93-11 type) and a homozygous C nucleotide base (PA64 type) at the M_9_ site as identified from amplification products of the N-haplotype-specific primer, primerN.

To identify the recombination types of recombinant lines, 13 additional polymorphic loci were selected as markers covering approximately 445 kb of the flanking region around the *SD1* gene (S2D Fig.). Each marker was genotyped through PCR amplification followed by Sanger sequencing (S4 Table and S5 Table). Based on the genotypes of 93-11 and PA64s, each marker was assigned to genotype P (PA64s homozygosity), N (93-11 homozygosity) or H (heterozygosity of PA64s and 93-11). According to broadly accepted patterns of mitotic recombination (S1 Fig.), outcomes of CO events present heterozygous genotypes on one side and homozygous genotypes on another side separated by a breakpoint, whereas outcomes of NCO-GC events show heterozygous genotypes on both flanking regions of the tract but homozygous genotypes in the tract. In addition, in light of widespread crossover interference [8], we arbitrarily assigned that outcomes with homozygous genotypes on both flanking regions of the long tracts (>100 kb) may come from two adjacent CO events, whereas those of the short tracts may come from a CO event out of the *SD1* locus with a conversion event in the *SD1* gene. Other complex combinations of multiple genotype switches may be caused by combinations of long-tract conversions, CO-associated conversions or repetitive CO events. Finally, according to the different outcomes listed in Supplementary fig. S2d, we could identify the recombination types of tall individuals. Tracts of all recombination events were determined by the midpoint method [9,10].

In the other four additional crosses, which included both recombinant individuals and three adjacent individuals with normal plant heights as controls (S2B Fig.), we genotyped the *SD1* gene and its flanking region using the same 15 markers of 93-11 and PA64s in same manner as described above. We found that all samples from these additional four crosses share the same genetic polymorphisms with LYP9 in *SD1* region, so here we employed those 15 markers to all individuals in further analysis. As expected, all tall individuals harbored wild-type *SD1* gene, whereas the controls with normal plant height harbored heterozygous defective *sd1* alleles (S7 Table). Using the same strategy of identification of recombination types described above, we could also clarify the recombination events in 24 tall individuals of these other additional four crosses.

**C. Simulation of conversion and crossover rate per mitosis in different situations**

According to previous studies, roots and above ground parts are differentiated after 25-cells per-embryo [11–13], but there is still no detailed report arbitrating on the number of stem cells giving rise to roots and the above ground parts (namely number of original cells of root meristem (RM) and shoot apical meristem (SAM)). Based on our data giving the proportion of heterozygous cells in roots and basal leaves, we suggest that the original stem cells generating tissues (both roots and above ground parts) are at least two cells and conversion events must happen before differentiation between roots and the above ground parts. Note that *SD1* is highly expressed in stems and leaves, but poorly expressed in roots [14], hence root prevalence is more likely to be independent of any cell-cell competition.

Here we make several assumptions: (1) as the conversion events appear in root and RM and SAM segregated at or after the 5th division (32-cell stage), so conversion events happened in first four cell divisions; (2) there is no cell competition in roots. Based on these, we simulate different numbers of original cells of RM and SAM, as 2, 3, 4 etc., respectively. Meanwhile, as the proportion of recombinant cells in each root samples is larger than 50%, we simulate the situation in which the proportion of original stem cells with recombination events are larger than 50% in root original stem cells (RM). We similarly presume that only recombinant cells belonging to the original stem cells of SAM or RM will affect the daughter cells and phenotypic changes (for example, plant height in this study) in subsequent divisions.

Then we suppose that there are $n$ cells in total in an embryo, with $m$ of them are recombinant cells, when RM and SAM are decided and segregated. Then, we assign that the number of original stem cells in RM and SAM as $c_{1}$ and $c_{2}$ respectively, meaning that all $c_{1}+c_{2}$ cells are coming from these $n$ cells. Then the calculation now comes to: what is the probability that the $c_{1}$ cells contain at least $b_{1}$ recombinant cells, meanwhile the $c_{2}$ cells contain at least $b_{2}$ recombinant cells?

At the beginning, to simplify calculation, let us define the binomial coefficient as follows:

$\binom{n}{m}=[x^{m}](1+x)^{n}=\frac{n!}{m!(n-m)!}$ (1)

We first consider an easier case, where only one group, as example, $c_{1}$ cells in RM, is calculated. We are concerned about the probability that at least $b_{1}$ recombinant cells are in $c_{1}$ cells. Suppose that this value is $f_{1}\left( n,m,c_{1},b_{1} \right)$, then we can simply enumerate over the exact number of recombinant cells contained. Suppose that $i$ recombinant cells are contained, then the probability will be the number of ways to sample $i$ recombinant cells out of $m$, multiplied by the number of ways to sample $c_{1}-i$ cells out of $n-m$. Hence, the result will be:

$f_{1}\left( n,m,c_{1},b_{1} \right)=\frac{1}{\binom{n}{c_{1}}}\sum_{i=b_{1}}^{c_{1}} \binom{m}{i}\binom{n-m}{c_{1}-i}$ (2)

Now we consider the case as two groups ($c_{1}$ and $c_{2}$). We can enumerate over the number of recombinant cells in the first group, and reduce the problem to the one-group case. Thus, the result is:

$f_{2}\left( n,m,c_{1},b_{1},c_{2},b_{2} \right)=\frac{1}{\binom{n}{c_{1}}}\sum_{i=b_{1}}^{c_{1}} \binom{m}{i}\binom{n-m}{c_{1}-i}f_{1}\left( n-c_{1},m-i,c_{2},b_{2} \right)$ (3)

Then, we consider several situations in which numbers of original stem cells of RM and SAM (namely $c_{1}$ and $c_{2}$) range from 2 to 4: **(1)** Both the RM and SAM have **2** original stem cells and at least **1** of these **2** cells is recombinant cell. **(2)** Both the RM and SAM have **3** original stem cells. At least **1** cell is a recombinant cell in SAM. In RM, the proportion of recombinant cell must be more than 50%, so at least **2** cells out of 3 are recombinant cells. **(3)** Both the RM and SAM have **4** original stem cells. At least **1** cell is a recombinant cell in SAM and at least **2** cells are recombinant cells in RM. **(4)** Both the RM and SAM have **4** original stem cells. At least **1** cell is recombinant cell in SAM and at least **3** cells is recombinant cells in RM. The number of cells for these four situations is in the table below:

|  | Original stem cells in RM and SAM (*c_1_* and *c_2_*) | Recombinant original stem cells in RM (≥) (*b_1_*) | Recombinant original stem cells in SAM (≥) (*b_2_*) |
| --- | --- | --- | --- |
| Situation 1 | 2 | 1 | 1 |
| Situation 2 | 3 | 2 | 1 |
| Situation 3 | 4 | 3 | 1 |
| Situation 4 | 4 | 4 | 1 |

We then substitute the values of *c_1_*, *b_1_*, *c_2_*, *b_2_* of these four situations in **Equation (2)** and there are a total of 32 cells after the 5^th^ cell division (*n* = 32). Here *m* is the number of recombinant cells in these 32 cells. Then probabilities for these four situations:

$$\begin{matrix} {P1\left( m \right)=f}_{2}(32,m,2,1,2,1) & =\frac{m(32-m)\left( \binom{30}{2}-\binom{31-m}{2} \right)+\binom{m}{2}\left( \binom{30}{2}-\binom{32-m}{2} \right)}{\binom{32}{2}\binom{30}{2}} \\ P2\left( m \right)=f_{2}(32,m,3,1,3,2) & =\frac{\binom{m}{2}(32-m)\left( \binom{29}{3}-\binom{31-m}{3} \right)+\binom{m}{3}\left( \binom{29}{3}-\binom{32-m}{3} \right)}{\binom{32}{3}\binom{29}{3}} \\ {P3\left( m \right)=f}_{2}(32,m,4,1,4,2) & =\frac{\binom{m}{2}\binom{32-m}{2}\left( \binom{28}{4}-\binom{30-m}{4} \right)+\binom{m}{3}(32-m)\left( \binom{28}{4}-\binom{31-m}{4} \right)+\binom{m}{4}\left( \binom{28}{4}-\binom{32-m}{4} \right)}{\binom{32}{4}\binom{28}{4}} \\ P4\left( m \right)=f_{2}(32,m,4,1,4,3) & =\frac{\binom{m}{3}(32-m)\left( \binom{28}{4}-\binom{31-m}{4} \right)+\binom{m}{4}\left( \binom{28}{4}-\binom{32-m}{4} \right)}{\binom{32}{4}\binom{28}{4}} \end{matrix}$$

For *m*, if event happened at the *i* cell division (1 ≤ *i* ≤ 4), then *m* = 2^5-^*^i^*, then the probability of being apportioned into RM and SAM of recombinant cells in the first four divisions for each situation will be:

$P\left\{ x \right\}=\sum_{i=1}^{4} 2^{i-1}P(m)$ (4)

We assigned *R_mi(GC)_* and *R_mi(CO)_* as the somatic conversion rate per cell division and somatic crossover rate per cell division. In this study, we found a total of six conversion events (3 NCO-GC events and 3 CO-GC events) and 19 CO events related *SD1* recovery (18 CO events and 1 CO-GC events (Type9 in fig. 2)) in 1,100,000 LYP9 F_1_ individuals. then:

$R_{mi(GC)}P\left\{ x \right\}=6/1100000$ (5)

$R_{mi(CO)}P\left\{ x \right\}=19/1100000$ (6)

Then, we can calculate the probability of apportion into RM and SAM of recombinant cells at the *i* division for each situation as table below:

|  | Cell division | Situation 1 | Situation 2 | Situation 3 | Situation 4 |
| --- | --- | --- | --- | --- | --- |
| Probability of apportion into RM and SAM of recombinant cells at the *i* division, *P(m)* | 1st | 0.56675 | 0.434483 | 0.659551 | 0.278675 |
|  | 2nd | 0.182617 | 0.074865 | 0.160192 | 0.022132 |
|  | 3rd | 0.045144 | 0.006834 | 0.017475 | 0.000442 |
|  | 4th | 0.008069 | 0 | 0 | 0 |
| *R_mi(GC)_* | | 4.63×10^-6^ | 8.92×10^-6^ | 5.20×10^-6^ | 1.68×10^-5^ |
| *R_mi(CO)_* | | 1.46×10^-5^ | 2.82×10^-5^ | 1.64×10^-5^ | 5.32×10^-5^ |

In all, *R_mi(GC)_* for these four situations in order as: 4.63 × 10^-6^, 8.92 × 10^-6^, 5.20 × 10^-6^ and 1.68 × 10^-5^, and *R_mi(CO)_* for these four situations in order as: 1.46 × 10^-5^, 2.82 × 10^-5^, 1.64 × 10^-5^and 5.32 × 10^-5^. In addition, recombination events happening in the first two cell divisions contribute the largest part of the probability in each situation.

As shown in below figure, recombinant cells (red cycles) occurred in the first several divisions. When roots and above ground part are beginning to be differentiated, only recombinant cells that belong to the original stem cells of SAM (blue background) or RM (green background) will be calculated and affect the daughter cells (also phenotypic changes) in subsequent divisions. While, other recombinant cells (gray background) which are not in the SAM or RM, will not affect the daughter cells and the phenotypic changes. In other words, our detection system is underestimated, owing to missing the recombinant events which are not in RM or SAM.


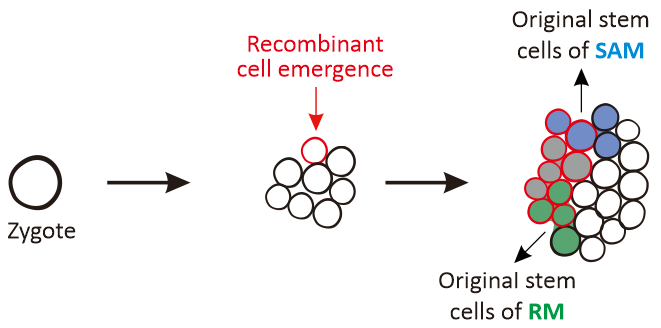


On the other hand, we can calculate the probability that 100% original cells are recombinant cells in RM and at least one cell in SAM. We simulate the original cell number in both RM and SAM as 2, 3 and 4 by the **Equation (2)**. Then for 2,3 and 4 original cells, these probabilities are 12.4%, 7.5% and 2.7%. Actually, in our data, five samples out of six conversion individuals have cell proportion data in roots and one of these five samples are 100% recombinant cells in roots (namely 20% samples harboring 100% recombinant cell in roots), this is close to the situation that two original cells in RM and SAM.

**Supplemental References:**

1. Prado F, Cortés-Ledesma F, Huertas P, Aguilera A. Mitotic recombination in Saccharomyces cerevisiae. Curr Genet. 2003;42: 185–198. doi:10.1007/s00294-002-0346-3

2. Mandegar MA, Otto SP. Mitotic recombination counteracts the benefits of genetic segregation. Proc R Soc Lond B Biol Sci. 2007;274: 1301–1307. doi:10.1098/rspb.2007.0056

3. Yin Y, Petes TD. Genome-Wide High-Resolution Mapping of UV-Induced Mitotic Recombination Events in Saccharomyces cerevisiae. PLoS Genet. 2013;9: e1003894. doi:10.1371/journal.pgen.1003894

4. Mehta A, Haber JE. Sources of DNA Double-Strand Breaks and Models of Recombinational DNA Repair. Cold Spring Harb Perspect Biol. 2014;6: a016428. doi:10.1101/cshperspect.a016428

5. Yin Y, Dominska M, Yim E, Petes TD. High-resolution mapping of heteroduplex DNA formed during UV-induced and spontaneous mitotic recombination events in yeast. de Massy B, editor. eLife. 2017;6: e28069. doi:10.7554/eLife.28069

6. Lisby M, Rothstein R. Cell Biology of Mitotic Recombination. Cold Spring Harb Perspect Biol. 2015;7: a016535. doi:10.1101/cshperspect.a016535

7. Syeda AH, Hawkins M, McGlynn P. Recombination and Replication. Cold Spring Harb Perspect Biol. 2014; a016550. doi:10.1101/cshperspect.a016550

8. Hillers KJ. Crossover interference. Curr Biol. 2004;14: R1036–R1037. doi:10.1016/j.cub.2004.11.038

9. Mancera E, Bourgon R, Brozzi A, Huber W, Steinmetz LM. High-resolution mapping of meiotic crossovers and non-crossovers in yeast. Nature. 2008;454: 479–485. doi:10.1038/nature07135

10. Liu H, Huang J, Sun X, Li J, Hu Y, Yu L, et al. Tetrad analysis in plants and fungi finds large differences in gene conversion rates but no GC bias. Nat Ecol Evol. 2018;2: 164–173. doi:10.1038/s41559-017-0372-7

11. Goldberg RB, Paiva G de, Yadegari R. Plant Embryogenesis: Zygote to Seed. Science. 1994;266: 605–614. doi:10.1126/science.266.5185.605

12. Agarwal P, Kapoor S, Tyagi AK. Transcription factors regulating the progression of monocot and dicot seed development. BioEssays. 2011;33: 189–202. doi:10.1002/bies.201000107

13. Zhao T, Holmer R, Bruijn S de, Angenent GC, Burg HA van den, Schranz ME. Phylogenomic Synteny Network Analysis of MADS-Box Transcription Factor Genes Reveals Lineage-Specific Transpositions, Ancient Tandem Duplications, and Deep Positional Conservation. Plant Cell. 2017;29: 1278–1292. doi:10.1105/tpc.17.00312

14. Sasaki A, Ashikari M, Ueguchi-Tanaka M, Itoh H, Nishimura A, Swapan D, et al. Green revolution: A mutant gibberellin-synthesis gene in rice. Nature. 2002;416: 701–702. doi:10.1038/416701a
